# Analysis of Sequence and Copy Number Variants in Canadian Patient Cohort With Familial Cancer Syndromes Using a Unique Next Generation Sequencing Based Approach

**DOI:** 10.3389/fgene.2021.698595

**Published:** 2021-07-13

**Authors:** Pratibha Bhai, Michael A. Levy, Kathleen Rooney, Deanna Alexis Carere, Jack Reilly, Jennifer Kerkhof, Michael Volodarsky, Alan Stuart, Mike Kadour, Karen Panabaker, Laila C. Schenkel, Hanxin Lin, Peter Ainsworth, Bekim Sadikovic

**Affiliations:** ^1^Molecular Genetics Laboratory, Molecular Diagnostics Division, London Health Sciences Centre, London, ON, Canada; ^2^Department of Pathology and Laboratory Medicine, Western University, London, ON, Canada; ^3^Department of Pathology and Laboratory Medicine, London Health Sciences Centre, London, ON, Canada; ^4^Medical Genetics Program of Southwestern Ontario, London Health Sciences Centre, London, ON, Canada

**Keywords:** next generation sequencing, copy number variants, familial cancer syndromes, breast cancer, colorectal cancer

## Abstract

**Background:**

Hereditary cancer predisposition syndromes account for approximately 10% of cancer cases. Next generation sequencing (NGS) based multi-gene targeted panels is now a frontline approach to identify pathogenic mutations in cancer predisposition genes in high-risk families. Recent evolvement of NGS technologies have allowed simultaneous detection of sequence and copy number variants (CNVs) using a single platform. In this study, we have analyzed frequency and nature of sequence variants and CNVs, in a Canadian cohort of patients, suspected with hereditary cancer syndrome, referred for genetic testing following specific genetic testing guidelines based on patient’s personal and/or family history of cancer.

**Methods:**

A 2870 patients were subjected to a single NGS based multi-gene targeted hereditary cancer panel testing algorithm to identify sequence variants and CNVs in cancer predisposition genes at our reference laboratory in Southwestern Ontario. CNVs identified by NGS were confirmed by alternative techniques like Multiplex ligation-dependent probe amplification (MLPA).

**Results:**

A 15% (431/2870) patients had a pathogenic variant and 36% (1032/2870) had a variant of unknown significance (VUS), in a cancer susceptibility gene. A total of 287 unique pathogenic variant were identified, out of which 23 (8%) were novel. CNVs identified by NGS based approach accounted for 9.5% (27/287) of pathogenic variants, confirmed by alternate techniques with high accuracy.

**Conclusion:**

This study emphasizes the utility of NGS based targeted testing approach to identify both sequence and CNVs in patients suspected with hereditary cancer syndromes in clinical setting and expands the mutational spectrum of high and moderate penetrance cancer predisposition genes.

## Introduction

Hereditary cancer predisposition syndromes account for up to 10% of all diagnosed cancer cases. Initially, primary genetic testing was limited to the high penetrance genes *BRCA1* and/or *BRCA2*, which account for approximately 3–5% of breast cancers and 12–15% of ovarian cancers in most populations (Balmaña et al., 2011; Lang et al., 2017; Li et al., 2019). However, it is now established that additional hereditary cancer predisposition syndromes are linked to an ever increasing number of genes, including but not limited to *TP53*, *CDH1*, *SKT11*, *PTEN*, *PALB2*, *MLH1*, *MSH2*, *PMS1*, *PMS2*, and *MSH6*, which have also been associated with increased risk of breast, ovarian, and other cancers, often as part of more complex family histories including colon, endometrial, gastric, brain, and/or other cancers (Rahman et al., 2007; Roberts et al., 2018; Schon and Tischkowitz, 2018). Advances in next generation sequencing (NGS) technologies have uncovered a variety of new genes conferring variable levels of cancer risk. Ease of testing of multiple genes simultaneously, along with reduced cost and rapid turnaround times has enabled implementation of this technique as a frontline clinical test for individuals suspected to have a familial cancer syndrome (Okur and Chung, 2017). NGS technologies have also been implemented in rapid, cost-effective and high-throughput identification of copy number variants (CNVs) along with sequence variants. CNVs constitute a class of structural genetic variant involving increase or decrease in the number of copies of specific regions of DNA. CNVs in cancer susceptibility genes constitute for a significant number of pathogenic variants (PVs) (approximately 7%) and can be identified using NGS based algorithms with significant reduction in turnaround times and cost (Mancini-DiNardo et al., 2019).

Our laboratory has previously validated a unique NGS-based pipeline for detection of sequence and copy number variants (CNVs), in a panel of known cancer susceptibility genes and have been serving as a reference laboratory for genetic susceptibility testing for cancer within the province of Ontario, Canada for the past two decades (Schenkel et al., 2016; Kerkhof et al., 2017). In the present study we have analyzed genetic test results in 2870 patients analyzed using this NGS pipeline for hereditary cancer gene predisposition, referred for genetic testing to Molecular Diagnostics Laboratory the London Health Sciences Centre (LHSC), a provincial testing center in Southwestern Ontario. This testing is publicly funded for patients who meet eligibility criteria specified by the Ministry of Health and Long-term Care (MOHLTC).

The largest subset of these patients had been referred due to a personal or family history of hereditary breast and/or ovarian cancer, and were typically identified using provincial [Ontario Breast Screening Program (OBSP)]^1^ guidelines for referral, using a similar approach to that described by the National Comprehensive Cancer Network (NCCN)^2^. However significant subset of our patients was referred due to a personal and/or family history of other types of cancers, including uterine, colorectal, gastric, and/or other cancers, as well as colonic polyposis.

In this study we have described the spectrum of reportable genetic sequence variants identified, which include a significant proportion of deleterious and previously unreported sequence variants and CNVs which by using this approach can be identified routinely at a sub-exon resolution for multiple genes, in parallel, with high accuracy. We have analyzed frequency of variants in moderate and high penetrance gene among patients presenting with various cancer types.

## Materials and Methods

### Data Sources

This study involved assessment of anonymized and de-identified data from a clinical laboratory database, including genetic test findings along with key clinical and demographic features, at the Molecular Diagnostics Laboratory at London Health Sciences Centre during time period from January 2016 to May 2018. All clinical and demographic details in the laboratory database were obtained from the Test Request Forms (TRFs), submitted by the ordering physician or healthcare provider, that is kept as part of routine laboratory quality control protocols. The guidelines for referral for genetic testing have been described in Supplementary Table 1. Blood samples were received by the laboratory as part of routine clinical diagnostic testing, for which patients received counseling and provided informed consent in reference cancer genetics clinics in Ontario. Anonymized summary data in the laboratory Quality Management database was presented in compliance with the lab Quality Improvement protocol. The genomic DNA was isolated by standard protocols using the MagNA Pure system (Roche Diagnostics, Laval, QC, Canada).

### NGS Panels

**FIGURE 1:**
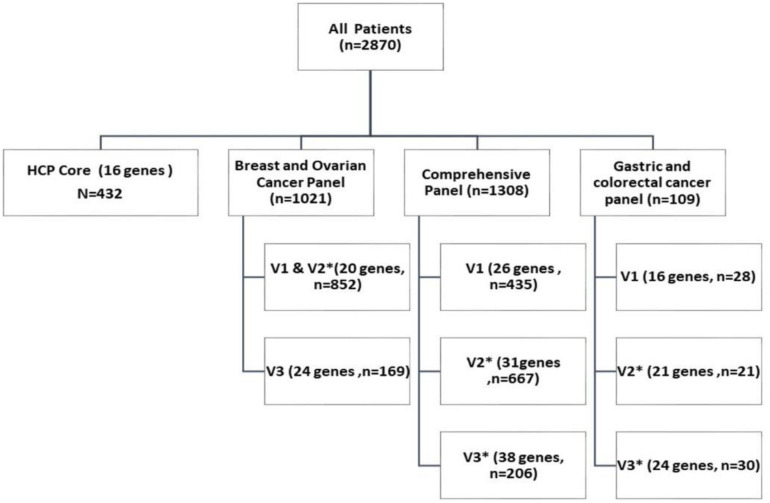
Number of patients tested for hereditary cancer predisposition with multi gene targeted panel test: This chart describes number of patients tested with each sub-panel and versions. Each sub-panel and its respective version constitute of a set of genes associated with increased predisposition to specific cancers (List of genes included is described in Supplementary Table 2).

A custom in-house developed Comprehensive Cancer Panel initially included 26 known cancer predisposition genes (comprehensive version v1), while subsequent updates and expansions of this panel are designated as v2 and v3 included 31 and 38 genes, respectively (Genes listed in Supplementary Table 2). Two subpanels (for which genes were bioinformatically filtered and reported from the comprehensive panel), included genes associated with hereditary breast and ovarian cancer syndrome (24 genes in v.3), and hereditary gastric and/or colorectal cancer syndrome (24 genes in v.3). All samples underwent target enrichment and sequencing for the genes included in the comprehensive panel, however analysis and reporting was carried out according to the predefined subset of genes as per the ordering physician’s submitted requisition form (Comprehensive; Breast and Ovarian Cancer; Gastric and Colorectal Cancer; HP16). Genes included in each panel and subsequent versions are described in Supplementary Table 2 and number of patients tested on each panel is shown in Figure 1. Our panel content was designed to meet the requirements set forth by the Ministry of Health and Long term Care of Ontario and is based on the clinical guidelines and recommendations including Cancer Care Ontario’s OBSP (see text footnote 1) and NIH ClinGen expert recommendations^3^ and National Centre for Biotechnology Information (NCBI) Gene Reviews^4^ along with genes reported in literature (Lee et al., 2019; Seifert et al., 2019).

### NGS Pipeline

Custom sequence capture probes were designed using the SeqCap EZ Choice Library system (Roche NimbleGen, Inc., Madison, WI, United States). The design included enrichment for all coding exons as well as 20 bp of the flanking intronic regions. Normally, 100 ng of genomic DNA was used for library preparations. Library preparation and target capturing sequencing steps were performed as previously described (Schenkel et al., 2016; Kerkhof et al., 2017).

### Sequence Analysis and Variant Assessment

Sequence alignment, coverage distribution and variant identification were performed with NextGene software version 2.4.1 (SoftGenetics, LLC) using standard alignment settings. BAM and VCF files were imported into Geneticist Assistant version1.1.5 (SoftGenetics, LLC) for quality control assessment as previously described (Schenkel et al., 2016; Kerkhof et al., 2017). Variants were analyzed by a clinical molecular geneticist and classified for pathogenicity as per published American College of Medical Genetics (ACMG) guidelines (Richards et al., 2015). All of the variants classified as ACMG 1 (pathogenic), 2 (Likely pathogenic), or 3 [variant of unknown significance (VUS)] were confirmed by sanger sequencing or targeted molecular assessment as appropriate.

### Copy Number Variants (CNV) Analysis and Confirmation

CNV analysis was performed following methods previously validated in our laboratory (Susswein et al., 2016). Briefly, coverage distribution reports were generated using NextGene software v2.4.1 (SoftGenetics). The normalization factor for each sample included in the capture was identified based on total coverage and then used to normalize each nucleotide of the entire panel. The normalized nucleotide value was then graphed using Excel v.14.0.6129.5000 (Microsoft Corporation) to identify exon as well as sub-exon variants. Deletions were identified by a ratio of ≤0.65 and duplications were identified by a ratio of ≥1.35. These cutoff values were calculated based on internal laboratory reference analysis as previously described. A four-allele normalization method was adapted to address pseudogene involvement (or another homologous region) for example *PMS2* gene. The coverage for the four alleles was totaled at each nucleotide position and normalization algorithm was performed. Deletions were defined by a mean ratio of ≤0.8 (3/4 alleles), whereas duplications were defined by a ratio of ≥1.2 (5/4 alleles). CNVs identified by this NGS analysis pipeline were confirmed and characterized by a second clinically validated method, including Multiplex Ligation-dependent Probe Amplification (MLPA), which was used to confirm CNVs in 35 patients. In three patients, breakpoints were identified using Long Range PCR (LR-PCR) followed by Sanger sequencing. All variants are reported using HGVS nomenclature (den Dunnen et al., 2016).

## Results

### Patients Details

Demographic details of the 2870 patients assessed in the study are included in Table 1. The number of patients tested on each subpanel (as per referring physician) is shown in Figure 1. Genetic variants were analyzed and classified according to criteria described by ACMG in five specific categories: ACMG 1 (pathogenic), ACMG 1 (likely pathogenic), ACMG 3 (variant of uncertain significance), ACMG 4 (likely benign), and ACMG 5 (Benign) (Richards et al., 2015). Genetic variants classified as ACMG 1, 2, and 3 were included in the patient’s test reports. For further analysis in this study, all variants categorized as ACMG 1 and 2 are referred to as PV and ACMG 3 variants are referred to as VUSs. Majority of patients in the cohort were female (90.3%; 2593/2870). Although a direct comparison is not possible due to a marked difference in sample size, higher frequency of PV was seen in males (21.3% vs. 14.3%). More than 50% of patients were in the age group of 50–70 years at the time of testing, and PV detection rates were slightly higher in younger age groups (<60 years). Ancestral origin or ethnicity information was available for 2181/2870 (76%) patients. Of these, approximately 84% (1828/2181) were of European ancestry, and 16% were from other ethnicities, including Asian, African, Indigenous Canadian, South American and mixed race (Table 1).

**TABLE 1:** Demographic details of patients included in the study (*N* = 2870).

	**Total patients**	**Percent of total patients**	**No. of patients with a pathogenic variant**	**Percent of patients with a pathogenic variant (within row)**
Summary	2870	100.0	431	15.0
Males	277	9.7	59	21.3
Females	2593	90.3	372	14.3
**Age (at testing)**				
<30	80	2.7	14	17.5
31–40	307	10.7	59	19.2
41–50	460	16.0	79	17.2
51–60	748	26.1	120	16.0
61–70	763	26.6	98	12.8
71–80	407	14.2	51	12.5
>80	105	3.7	10	9.5
**Population/Ancestry**				
European	1828	63.7	258	14.1
Unknown	689	24.0	124	18.0
Mixed Ancestry	83	2.9	7	8.4
South Asian	74	2.6	15	20.3
East Asian	66	2.3	7	10.6
Middle eastern	52	1.8	9	17.3
African	35	1.2	6	17.1
South American	32	1.1	5	15.6
Indigenous Canadian	11	0.4	0	0.0

### Pathogenic Variants

**FIGURE 2:**
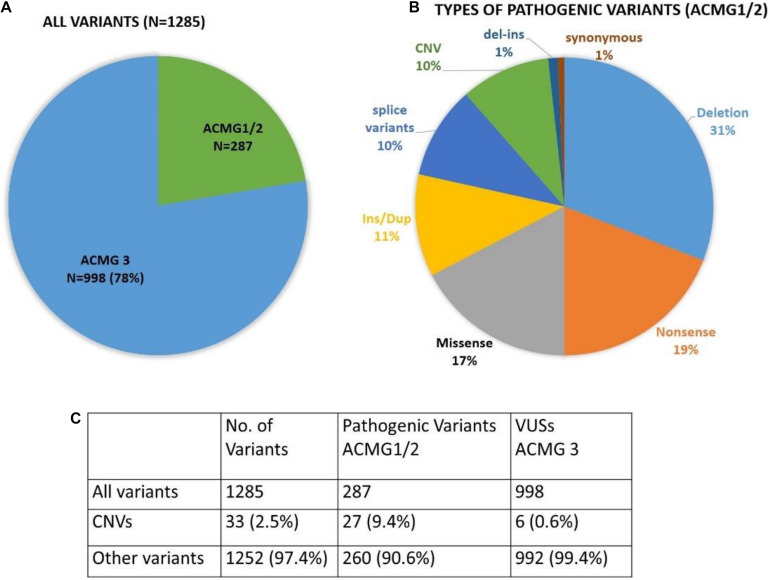
Frequency of pathogenic variants and variants of unknown significance identified in the study. This figure gives information about frequency of different types of variants **(A)** Pie chart showing number of pathogenic variants (ACMG 1 and 2) and ACMG 3 (variants of unknown significance), **(B)** Pie chart showing frequency of different types of pathogenic variants (ACMG 1 and 2), **(C)** Table showing frequency of copy number variants (CNVs) and sequence variants.

Out of 2870 patients, 431 tested positive for a PV, accounting for a 15% overall detection rate (including *MUTYH* mono-allelic and bi-allelic variants). Patients tested with the comprehensive panel had a slightly higher rate of PVs (16.2%) compared to other sub-panels, 13.6% for the Breast and Ovarian Cancer sub-panel (Supplementary Table 3). Although the number of patients analyzed with the Gastric and Colorectal Cancer sub-panel was relatively less, the PV detection rates were highest for this sub-panel (22%; 24 out of 109 tested positive for a PV). A total of 1285 reportable variants were identified; 287 (22%) were pathogenic (PV), and 998 (78%) were VUSs, (Figure 2). All variants identified in the study are listed in Supplementary Table 4. Ninety-two percent of PVs detected in this study have previously been described in the literature and/or ClinVar database, and 23 (8%) were novel, i.e., did not appear to be reported elsewhere (listed in Supplementary Table 5).

### Frequency of Pathogenic Variants in Specific Cancers

The number of patients with a PV in a high or moderate penetrance gene (as defined by NCCN; see text footnote 2), presenting with specific cancer phenotype is listed in Tables 2, 3. Overall the major cancer types seen in patients were breast, ovarian, colorectal, gastric, uterine, pancreas, prostate, brain and colonic polyposis. *BRCA1* and *BRCA2* genes had highest frequency of PVs among patients with ovarian cancer (5.4% in *BRCA1* and 4% in *BRCA2*) followed by patients with breast cancer (2.1% in *BRCA1* and 1.7% in *BRCA2*). Mismatch repair (MMR), genes had the highest frequency of PVs in the sub-group of patients presenting with colorectal cancer, (*MLH1*; 3%), *MSH2*; 1.8%, *MSH6*; 3%, *PMS2*; 1.1%) or uterine cancer (*MLH1*; 2.6%) and *MSH2*, *MSH6*, *PMS2* with1.3% each). Among patients presenting with colonic polyposis, *APC* gene had highest frequency of PVs (*APC*;4%) (Table 2). Out of 2870 patients tested, 2730 were diagnosed with cancer and 409 (14.9%) of these had a PV. About 140 patients were not diagnosed with any cancer, and had been referred for genetic testing based on criteria involving their family cancer history; 22 of these patients (15.7%) were positive for a PV in a cancer susceptibility gene.

**TABLE 2:** Frequency of pathogenic variants (PV) in patients presenting with various cancer types.

Cancer Types *	**Total Patients (%)^#^**	**Number of patients with a PV (% age calculated out of total patients with a specific cancer type)**		
		***All susceptibility genes on the panel***	***High penetrance genes***	***Moderate penetrance genes***
**Major Cancer types reported in patients**				
Breast	1548 (54%)	195 (12.5%)	BRCA1 (2.1%), BRCA2 (1.7%), PALB2 (1%), TP53 (0.4%)	CHEK2 (2.3%), ATM (1.3%), MMR genes (0.5%), BRIP1 (0.4%), BARD1, RAD51D, FANCM, RAD51C, NBN (0.2% each)
Ovarian	455 (16%)	81 (18%)	BRCA1 (5.4%), BRCA2 (4%), PALB2 (0.4%), TP53 (0.8%)	RAD51C (1.5%), RAD51D (1%), Mismatch repair gene (1.3%), ATM (1%), CHEK2 (0.6%), BRIP1 (0.4%)
Colorectal Cancer	167 (6%)	33 (19.7%)	MLH1 (3%), MSH2 (1.8%), MSH6 (3%), PMS2 (1.1%), APC (1.1%), TP53 (0.6%) Biallelic MUTYH (0.6%)	BRIP1, BARD1, ATM (0.6% each)
Polyps	100 (3.4%)	21 (21%)	APC (4%), PMS2 (2%), MUTYH (2%), MLH1 (2%), TP53 (1%), PTEN (1%), MSH6 (1%)	ATM (2%), BRCA2, CHEK2, SMAD4, (1% each)
Uterine Cancer	76 (2.6%)	12 (15.7%)	BRCA2 (4%), MLH1 (2.6%), MSH2, MSH6, PMS2 (1.3% each)	ATM, CHEK2, RAD51C, NTHL1 (1.3% each)
Gastric and other GI	51 (1.7%)	11 (21.5%)	CDH1 (2%), MSH2 (2%), PMS2 (2%), TP53 (4%)	ATM (2%), BRCA2 (6%)
Prostate Cancer	29 (1%)	3 (10.3%)	MLH1 (3.4%), CHEK2 (7%)	
Pancreatic Cancer	24 (0.8%)	2 (8.3%)	PALB2 (8.3%)	
**Patients with Rare/unspecified/No Cancer**				
Other cancers**	61 (2%)	9 (14.7%)	–	–
Unspecified Cancer	219 (7.6%)	42 (19%)	–	–
Total patients with a personal history of cancer	2730	409 (14.9%)	–	–
Patients with only family history of cancer without personal history of cancer reported	140 (4.8%)	22 (15.7%)	–	–

*Patients with personal history of multiple cancer types are included in the category of cancer type for which genetic testing/subpanel was ordered.

**Other cancer included brain cancer, hematological cancer, sarcoma, thyroid cancer and melanoma.

*#Percentage is calculated out of total 2780 patients.*

**TABLE 3:** Frequency of pathogenic variants in BRCA1 and 2 genes, other high and moderate penetrance genes in various high-risk categories of patients with breast and/or ovarian cancer.

**Breast and/or ovarian cancer High Risk categories**	**No of patients**	**Patients with pathogenic variants in all genes included in the panel**	**Patients with pathogenic variants in BRCA1 and BRCA2 genes only**	**Patients with pathogenic variants in 6 High Penetrance genes (BRCA1, BRCA2, PALB2, TP53, PTEN, CDH1)**	**Patients with pathogenic variants in moderate penetrance genes**	**Moderate penetrance genes contribution to each high-risk category**
Breast and/or ovarian cancer (at any age)	2003	276 (14%)	103 (5%)	130 (6.5%)	146 (7.5%)	CHEK2 (1.9%), ATM (1.2%), RAD51C (0.4%), RAD51D (0.4%), BRIP1 (0.4%), MMR genes (0.6%), less known gene BARD1 (0.1%), FANCM (0.1%), NBN (0.1%)
Breast and ovarian cancer (both diagnosed in one patient at any age)	49	13 (26.5%)	9 (18%)	11 (22.4%)	2 (4%)	RAD51C (2%), RAD51D (2%)
Breast cancer <35 year of age	186	27 (14.5%)	16 (8.6%)	20 (11%)	7 (3.5%)	CHEK2 (1.4%), ATM (1%), FANCM (0.5%), BARD1 (0.5%), MMR genes (0.5%)
Breast Cancer <45 years of age	482	63 (13%)	32 (6.6%)	38 (8%)	25 (5%)	CHEK2 (2%), ATM (0.8%), RAD51C/RAD51D (0.6%), BRIP1 (0.4%), FANCM (0.4%)
Ovarian cancer at any age	455	81 (18%)	43 (9.5%)	49 (11%)	32 (7%)	RAD51C (1.5%), RAD51D (1%), Mismatch repair gene (1.3%), ATM (1%), CHEK2/BRIP1 (1%)
Triple negative breast cancer	218	33 (15%)	19 (9%)	21 (9.6%)	12 (5.4%)	BRIP1 (1.8%), CHEK2 (1%), RAD51C (0.5%), RAD51D (0.5%), MMR genes (0.5%)
Bilateral breast cancer	135	12 (9%)	3 (2.2)	6 (4.4%)	6 (4.4%)	CHEK2 (1.5%), BRIP1 (0.7%), ATM (0.7%), MMR genes (0.7%)
Breast cancer with pancreas or prostate at any age	10	3 (30%)	1 (10%)	2 (20%)	1 (10%)	PALB2 (10%)

### Breast and/or Ovarian Cancer

**FIGURE 3:**
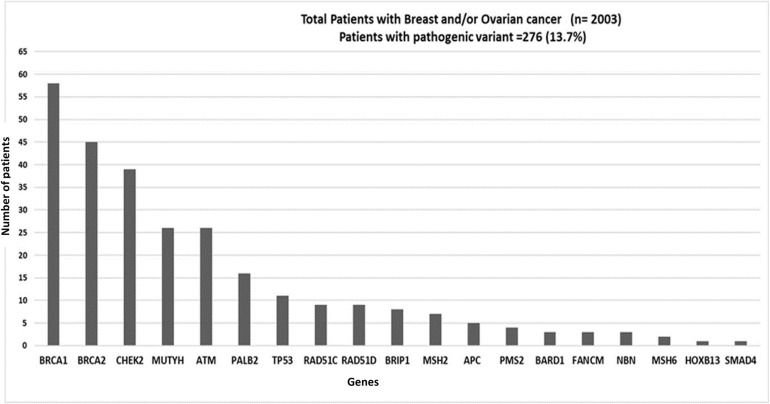
Graph showing distribution of pathogenic variants (PVs) in patients with breast and/or ovarian cancer: Patients presenting with Breast and/or Ovarian cancer (*n* = 2003). 13.7% (276/2003) patients tested positive for a PV. Genes with PVs are listed on the *X*-axis; Number of patients positive for a pathogenic variant is shown on *Y*-axis.

**FIGURE 4:**
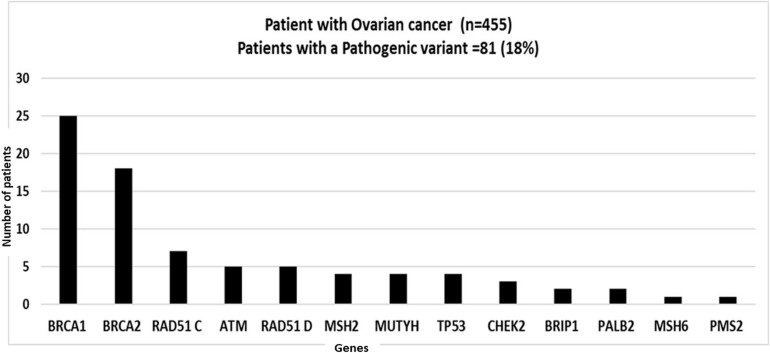
Graph showing distribution of pathogenic variants (PVs) in patients with ovarian cancer: Patients presenting with Ovarian cancer (*n* = 455). 18%(82/455) patients tested positive for a PV. Genes with PVs are listed on the *X*-axis; Number of patients positive for a pathogenic variant is shown on *Y*-axis.

Majority of this patient cohort (2003 out of 2870) patients presented with breast and/or ovarian cancer, and the frequency of patients with a PV in a cancer susceptibility gene is shown in Figure 3. Out of these patients the majority had been diagnosed with breast cancer (1548/2870; 54%: including 49 patients diagnosed with both breast and ovarian cancer) followed by patients with isolated ovarian cancer, (455/2870; 16%). PVs were detected in 12.5% of breast cancer patients, and 18% of ovarian cancer patients; the major genes and frequency of PVs are described in Figures 3, 4. Approximately 5% of patients presenting with breast cancer had a PV in one of the high penetrance genes, (*BRCA1*, *BRCA2*, *TP53*, *PALB2*, *CDH1*, and *PTEN*), and an additional 7.5% patients had a PV in one of the moderate penetrance genes (Table 2). Among ovarian cancer patients more than 9% harbored PVs in *BRCA1* and *BRCA2* genes. Notably, in patients with ovarian cancer, a significant number of PVs were identified in the moderate penetrance *RAD51* genes; *RAD51C* (1.5%) and *RAD51D* (1%), followed by the MMR and *ATM* genes (Table 2).

### Gastrointestinal Cancer and Polyposis

Approximately 11% of patients presented with gastrointestinal (GI), tract related disease; 6% with colorectal cancer, 3.4% with polyposis, and 1.7% with gastric and/or other GI tract cancers, (Table 2). Overall the PV detection rate was highest in this group: i.e., 19% with colorectal cancer, 21% with polyposis, and 21.5% with gastric cancer (Table 2). The major genes with PVs in patients with colorectal cancer included the MMR genes *MLH1*, *MSH2*, *PMS2*, and *MSH6*, (accounting for 9% of patients with a PV), followed by *APC*, *TP53*, *MUTYH*, *BARD1*, *ATM*, and *BRIP1*. Patients presenting with polyposis tested positive for PVs in *APC* (4%), *MUTYH* (2%), *PMS2* (2%), *MLH1* (2%), *TP53* (2%), and to a lesser extent in *PTEN*, *SMAD4*, and *ATM*. Among patients with gastric and other GI tract cancer, PV’s were found in *CDH1*, *TP53*, *ATM*, *MSH2*, and PMS2 genes (Table 2).

### Other Cancers (Uterine, Pancreas, Prostate)

Cancer types seen less frequently in our study included uterine (76 patients; 2.6%), pancreatic (24 patients; 0.8%), and prostate cancer (29 patients; 1%). Among the sub-group of patients with uterine cancer, PVs were identified in (12/76; 15.7%) MMR genes, as well as *BRCA2*, *ATM*, *RAD51C*, and *NTHL1*. Out of 24 patients with pancreatic cancer, two patients tested positive for a PV in the *PALB2* gene accounting for an 8.3% PV detection rate. Among 29 patients presenting with prostate cancer, only three patients had a PV (two in *CHEK2* and one in *MLH1*) (Table 2).

### Unspecified and Rare Cancer Types

The cancer type was not defined for 219 patients (7.6%), and 42 (19%) of these patients had a PV (Table 2). A total of 61 patients were reported to have rare cancer types including brain cancer, (12 patients; 1 with PV in the *MSH2* gene), thyroid cancer, (9 patients; 1 with PV in the *BRCA2* gene), melanoma (25 patients; 4 patients with PV in *PALB2*, *BRCA2* or the *CHEK2* gene), sarcoma (12 patients; 2 with a PV in *MLH1* and *CHEK2* gene, respectively) and hematological cancer (three patients).

### Identification of Clinical Sub-groups With Higher PV Detection Rates in High Penetrance Genes Among Breast and Ovarian Cancer Patients

We have analyzed pathogenic variant detection rates in 6 actionable high penetrance genes recommended by NCCN (see text footnote 2: *BRCA1*, *BRCA2*, *PALB2*, *TP53*, *PTEN*, and *CDH1*) in patients with breast and/or ovarian cancer, categorized in sub-groups based on specific clinical presentation (Table 3). It was observed that PV detection rates were higher in certain clinically categorized sub groups These include: (1) breast and ovarian cancer (both diagnosed in one patient at any age), 11/49 (22.4%), (2) breast cancer <45 years of age, 38/482 (8%), (3) breast cancer <35 year of age, 20/186 (11%), (4) ovarian cancer at any age, 49/455 (11%), (5) triple negative (ER/PR/HER-2/neu negative) breast cancer, (TNBC), 21/218 (9.6%), (6) breast cancer with pancreatic or prostate at any age, 2/10 (20%). Since these sub-groups of patients have higher PV detection rates in high risk genes, they can be defined as high- risk group of patients. It can be inferred that these high-risk sub-groups are candidates for immediate referral for genetic testing at initial clinical contact, to avoid any delay in obtaining genetic test results for these high-risk sub groups as many medical and/or surgical management options for these patients can be driven by these genetic test results.

### Copy Number Variations

**FIGURE 5:**
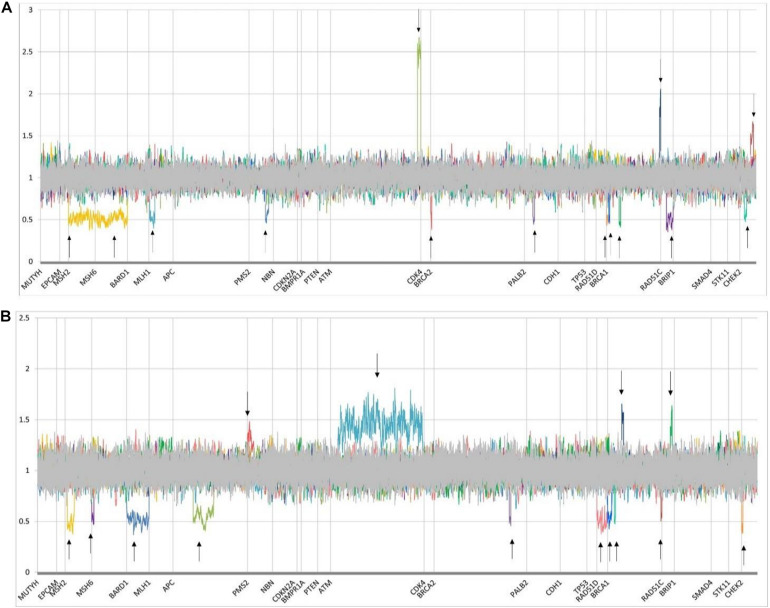
Normalized copy number variant (CNV) plots demonstrating deletions and duplications in genes on the hereditary cancer panel. *Y*-axis represents quantile normalized copy number data (for unique autosomal genes, 0.5 indicates 1 copy; 1 indicates 2 copies; and 1.5, 3 copies; for homologous autosomal genes with their pseudogene, 0.75 indicates 3 copies; 1, 4 copies; and 1.25, 5 copies). Constitutional deletions are defined by a mean ratio of ≤0.65, and duplications are defined by a ratio of ≥1.35. Homologous region PMS2/PMS2CL deletions and duplications are assessed by a ratio of <0.8 and >1.2, respectively. *X*-axis indicates gene locations. From left to right, arrows represent patients with the following CNVs. **(A)** single patient with deletions in MSH2 and MSH6 genes [MSH2:c.(?_-21)_(*21_?)del/MSH6:c.(?_-21)_(*21_?)del] (represented by 2 arrows), pathogenic deletions of MLH1, PMS2, and BRCA2 [MLH1:c.(116 + 21_11721)_(545 + 21_546-21)del, PMS2:c.(537 + 21_538-21)_(903 + 21_904-21)del, BRCA2:c.(?_-21)_(67 + 21_68-21)del], novel VUS with four copies of a region of ATM [ATM:c.(8850 + 21_8851-21)_(*21_?) (Schon and Tischkowitz, 2018)], pathogenic deletions of PALB2 and BRCA1 [PALB2:c.(2586 + 21_2587-21)_(2748 + 21_2749-21)del, BRCA1:c.(5406 + 21_5407-21)_(*21_?)del, BRCA1:c.(5332 + 21_5333-21)_(5406 + 21_5407-21)del, BRCA1:c.(4357 + 21_4358-21)_(4484 + 21_4485-21)del], duplication of unknown significance in BRCA1 [BRCA1:c.(?_-21)_(80 + 21_81-21) (Li et al., 2019)], novel pathogenic deletion in RAD51C [RAD51C: c.(571 + 21_572-21)_(*21_?)del], pathogenic deletion and duplication of CHEK2 [CHEK2:c.(908 + 21_909-21)_(1095 + 21_1096-21)del, CHEK2:c.(319 + 21_320-21)_(592 + 21_593-21)dup], respectively. **(B)** Pathogenic deletions in MSH2, MSH6, BARD1, and APC genes [MSH2:c.(366 + 21_367-21)_(1076 + 21_1077-21)del, MSH6:c.(?_-21)_(260 + 21_261-21)del, BARD1:c.(?_-21)_(*21_?)del, APC:c.1958 + 241_4457del], duplications of unknown significance in PMS2 and ATM (novel variant) [PMS2:c.(2006 + 21_2007-21)_(*21_?)dup, ATM:c.(662 + 21_663-21)_(9171 + 21_9172-21)dup], pathogenic deletions in BRCA2, RAD51D (novel variant) and BRCA1 [BRCA2:c.(8487 + 21_8488-21)_(8632 + 21_8633-21)del, RAD51D:c.(?_-21)_(*21_?)del, BRCA1:c.(5277 + 21_5278-21)_(*21_?)del, BRCA1:c.(4986 + 21_4987-21)_(5074 + 21_5075-21)del], pathogenic duplication of BRCA1 [BRCA1:c.(4185 + 21_4186-21)_(4357 + 21_4358-21)dup], pathogenic deletion in BRCA1 [BRCA1:c.(?_-21)_(80 + 21_81-21)del], pathogenic duplication of RAD51C [RAD51C:c.(837 + 21_838-21)_(965 + 21_966-21)dup], pathogenic deletion of CHEK2 [CHEK2:c.(1461 + 21_1462-21)_(*21_?)del].

Whole gene or intragenic copy number variants were detected in 32 of 2870 (1.1%) patients, with confirmation by alternate techniques as shown in Supplementary Table 6. CNVs constituted 2.5% (33/1285) of total variants, and almost one in ten (9.5%; 27/287) of all PVs (Figure 2). 37% (10/27) of the pathogenic CNVs identified were in three (*BRCA1*, *BRCA2*, and *PALB2*) of the six high penetrance breast and/or ovarian cancer genes (*BRCA1*, *BRCA2*, *PALB2*, *TP53*, *PTEN*, and *CDH1*) identified by the NCCN (see text footnote 2). *BRCA1* and *BRCA2* deletions and duplications accounted for the majority, i.e., 33% (9/27) of the pathogenic CNVs detected. Normalized CNV plots showing CNVs identified in this study are represented in Figure 5. Frequency of different types of PVs (CNVs, nonsense, missense, deletion, insertions) is depicted in Figure 2. Detection of 25–150 bp “Mid-size” insertions and deletions can be challenging for NGS-based CNV algorithm. NGS alignment parameters require 80% match of a 150 bp reads for a successful alignment. Hence, the NGS alignment algorithm is normally limited to detection of in/dels <30 bp. However, the CNV-calling algorithm which is performed in parallel using the read depth data in a 10 bp sliding window is designed to enable detection of CNV >30 bp. As part of our standard clinical testing protocol all variants detected by NGS (sequence or CNV) are confirmed by and alternate methodology before a clinical report is issued. This protocol is designed to rule out have excluded the possibility of any technical artifacts that may result from NGS analysis and include standard clinically validated methodologies such as MLPA, LR-PCR, and Sanger sequencing (Supplementary Table 6).

## Discussion

An increasingly complex pattern of germline mutations is being detected in patients presenting with various types of cancers following the almost universal adoption of NGS-based genetic test protocols in diagnostic laboratories. Many new cancer susceptibility genes have been identified, and the frequency of mutations and associated clinical cancer phenotypes continue to be described in a wide variety of different ethnic groups across the world. Methods for detection of PVs in cancer predisposition genes have evolved significantly from the gold standard sanger sequencing and MLPA to most advanced NGS based massive parallel sequencing methods (Susswein et al., 2016; Okur and Chung, 2017; Rosenthal et al., 2017; Neben et al., 2019; Oliver et al., 2019; Tsaousis et al., 2019; LaDuca et al., 2020; Yadav et al., 2020). We have implemented a NGS-based approach that we have developed and optimized for detection of sequence variants and CNVs, in our laboratory, to test families suspected with familial cancer syndromes (Schenkel et al., 2016; Kerkhof et al., 2017; Volodarsky et al., 2020). The work flow and analysis algorithm is unique and highly accurate. Here we describe the outcome of a large cohort of patients referred to be tested using this approach at the Molecular Diagnostic Laboratory, London Health Sciences Centre (LHSC), an academic health care center serving a catchment area of approximately 2 million people in Southwestern Ontario, Canada. The distribution and prevalence of clinically relevant variants has been analyzed among patients tested with different panels of genes as described above, based on their personal and family history of cancer.

We report an overall detection rate of 15% for pathogenic/likely pathogenic mutations. Previous studies have reported comparable diagnostic yield, though a direct comparison has not been possible due to variability in patient inclusion criteria, number of genes on the variant detection and classification criteria (Susswein et al., 2016; Rosenthal et al., 2017; Neben et al., 2019; Oliver et al., 2019; Tsaousis et al., 2019; LaDuca et al., 2020; Yadav et al., 2020). Our panel content was designed to meet the requirements set forth by the Ministry of Health and Long term Care of Ontario and is based on the clinical guidelines and recommendations including Cancer Care Ontario’s OBSP (see text footnote 1) and NIH ClinGen expert recommendations (see text footnote 3) and NCBI Gene Reviews (see text footnote 4) along with genes reported in literature (Lee et al., 2019; Seifert et al., 2019). The panel content and gene number has expanded over the years based on these requirements. As a comparison in a recent study with an extended targeted panel, including 64 hereditary cancer predisposition genes, PV detection rate of 19.2% was reported in 496 patients with hereditary breast and ovarian cancer and 12% patients were positive for a PV in *BRCA1* and *BRCA2* genes (Shin et al., 2020). Another study with 143 gene targeted panel showed a PV detection rate of 17% (Oliver et al., 2019). Extended panels with more genes can yields increased PV detection rates but patient selection criteria and PV detection rates for individual high/low/unknown penetrance differ between the labs and are often variably reported in the overall detection rates. Also, the supporting evidence for clinical impact of variants in newly identified genes included in larger panels is often limited (Seifert et al., 2019). However, a recent hospital–based study has reported a 5.7% mutation detection rate in 6 actionable high penetrance breast cancer genes in a subset of patients presenting with breast cancer who were selected by NCCN clinical criteria for testing (Yadav et al., 2020). In our center, for similar patients selected by the equivalent OBSP clinical criteria, the mutation detection rate of the same subpanel of genes was 6.5% (Table 3). A recent prospective multi-center study has shown very high germline pathogenic variant detection rates (13.3%) in 2984 cancer patients not selected based on cancer type, disease stage, family history of cancer ethnicity, or age, tested by 80 gene NGS panel with nearly 30% of patients had their treatments impacted due to this information (Samadder et al., 2020). These results are definitely suggestive of the fact that guidelines used for selecting patients for genetic testing should be re-evaluated as the number of genes on NGS based targeted panels are expanding or approaches like universal multigene panel testing may be implemented in near future.

### Copy Number Variants (CNVs)

Intragenic copy number changes have been identified in hereditary cancer and account for a small, but clinically significant, proportion of cases. While the majority of *BRCA1* and *BRCA2* variants are loss of function sequence changes, intragenic large genomic rearrangements have been reported to account for 3–15% (Vasickova et al., 2007; Hansen et al., 2009) of all *BRCA1* and *BRCA2* mutations. Until more recently, copy number variant detection in breast and ovarian cancer has been limited to studies of *BRCA1* and *BRCA2* genes using approaches such as MLPA, long-range and/or quantitative PCR in the primary screen, and the contribution of CNVs in other hereditary cancer predisposition genes was relatively unknown. Our laboratory has been among the first few in the world to report a clinically validated copy number variant detection algorithm that could be applied to NGS panel testing, and which was initially applied to our hereditary breast cancer panel (Schenkel et al., 2016; Kerkhof et al., 2017). These computational methods have been demonstrated to accurately detect intragenic copy number alterations of >50 bp, such as exon deletions and/or duplications, and have enabled us to detect PVs across an entire panel of genes while decreasing costs through avoidance of parallel testing, an approach, which has led to decreased test turnaround times. Our analysis pipeline is designed to circumvent challenges of detecting intermediate in/del variants using a combination of sequence alignment and high-resolution CNV calling. Sequence alignment is based on the random DNA fragmentation resulting in a staggered sequence alignment with a deep sequence coverage (>200×). This enables the CNV calling algorithm to use a sliding scale window which is designed to asses for loss or gain of sequence coverage at a 10 bp resolution. This analysis is dependent on high quality of DNA and inter/intra run sequence uniformity, and absolute sensitivity for the complex variants cannot be guaranteed in specimens not meeting the specific quality control parameters. In our experience, in peripheral blood DNA, this applies to less than 2% of specimens where the data quality is suboptimal. Similar limitation for detection of such variants is common even with targeted orthogonal methods such as Sanger sequencing and MLPA analysis. CNV detection using NGS data increases the diagnostic yield of this assay, while decreasing the need for the orthogonal testing modalities, hence increasing the TAT and overall costs to the system. However, it also must be recognized that it is possible that some more complex rearrangements, low-level mosaicism, balanced translocations and other complex genomic features may have a limited detection using this methodology and result in false negatives. This however is not unlike other genomics testing methods including the classic techniques such as MLPA^5^ and copy number microarrays as well as other NGS-based CNV detection algorithms (Park and Mori, 2010; Zhao et al., 2013). This study has identified pathogenic (ACMG 1 and ACMG 2) CNVs in 32 of 2870 (1.1%) patients, or 32 of 431 (7.4%) of all PVs identified, a proportion roughly in line with few previous reports (Mancini-DiNardo et al., 2019; Tsaousis et al., 2019; Zeng et al., 2020). A significant subset of these CNVs have been identified in some of the less well characterized cancer predisposition genes and adds to the spectrum of variants reported in these genes (Table 2). These findings emphasize the benefit of routinely incorporating CNV analysis into NGS panel testing, an approach that will significantly improve the efficiency of screening for hereditary cancers. Moving forward, this type of CNV analysis pipeline will become indispensable as further studies suggest expanding the gene testing criteria (Yadav et al., 2020; Zeng et al., 2020) and will serve to highlight the cost-effectiveness of panel-based testing for all cancer patients and/or population screening for cancer prevention (Manchanda et al., 2018; Sun et al., 2019).

We have also observed variants in few genes that have a less well-established association with certain cancer types previously reported in the literature. PVs in the *ATM* gene have been reported to have an increased predisposition to develop a wide range of cancers including breast, ovarian, pancreatic, colorectal, uterine, gastric, and/or prostate cancers as well as colorectal polyps consistent with our own findings (Supplementary Table 7) (Thompson et al., 2005; Sriramulu et al., 2019). A PV in the *APC* gene, (c.3920T > A, p.(Ile1307Lys) was detected in 5 unrelated patients with breast cancer. This sequence variant has been reported as a low penetrance allele common in the Ashkenazi Jewish population, and its association with breast cancer risk has been controversial (Redston et al., 1998; Woodage et al., 1998). However, this association is supported by our findings which also suggest increased breast cancer risk in patients carrying this variant. We have also identified a PV in *BRIP1* gene in a patient presenting with colorectal cancer (Supplementary Table 7). *BRIP1* gene is reported to moderately increase the risk of breast and/or ovarian cancer, (OMIM: #605882) though its association with colorectal cancer is sparsely reported in the literature and exact risks are not known (Ali et al., 2019). As the list of genes, associated with an increased predisposition to cancer increases, more extensive studies will be needed to obtain a better understanding of the cancer risk patterns for these genes.

## Conclusion

This study correlates clinical presentations and associated PVs in a large anonymized data set of patients with personal and/or family history of cancer, referred for genetic testing over a 2-year period to a provincial genetic testing laboratory in Southwestern Ontario, Canada. It is recognized that that there are some limitations to this study in that in this anonymized data set we were not able to confirm clinical information provided on the test requisition, and in a minority of cases this information was incomplete. However, this study has provided an overview of the pattern of DNA sequence variants and genomic rearrangements identified in variety of cancers using a unique NGS based algorithm in a clinical setting. In addition, our findings do serve to highlight some key aspects related to the genetic testing offered in the province that may help to provide a more effective approach to identify and immediately refer for testing higher risk clinical subgroups who would benefit from more timely accession to their genetic test results, which will serve to better manage their ongoing clinical care.

## Data Availability Statement

The original contributions presented in the study are included in the article/Supplementary Material, further inquiries can be directed to the corresponding author/s.

## Ethics Statement

Ethical review and approval was not required for the study on human participants in accordance with the local legislation and institutional requirements. Written informed consent to participate in this study was provided by the participants’ legal guardian/next of kin.

## Author Contributions

PB did data curation, wrote original draft, performed data analysis, and conceptualization. ML did data curation, analysis, manuscript reviewing, and editing. KR contributed in writing original draft and data analysis. DC contributed in conceptualization and reviewing manuscript. JR did data curation and analysis. JK contributed in data curation, method validation, and analysis. MV contributed to the manuscript review and editing. AS did data curation and validation of methods. MK contributed to the conceptualization, arrange resources, and manuscript review and editing. KP contributed to the manuscript review and writing. LS and HL did validation of methods, manuscript review, and writing. PA contributed to the supervision, visualization, and writing original manuscript draft. BS contributed to the investigation, supervision, visualization, conceptualization, resources, funding acquisition, writing–reviewing and editing. All authors contributed to the article and approved the submitted version.

## Conflict of Interest

The authors declare that the research was conducted in the absence of any commercial or financial relationships that could be construed as a potential conflict of interest.

## Footnotes

1 https://www.cancercareontario.ca/en/cancer-care-ontario/programs/screening-programs/ontario-breast-obsp

2 nccn_guidelines_genfamhighrisk_breastovarianpancreatic_v1.2020_1.pdf

3 https://clinicalgenome.org/

4 https://www.ncbi.nlm.nih.gov/books/NBK1116/

5 www.mrcholland.com

## Supplementary Material

Supplementary Table 1 Guidelines for referral for hereditary cancer predisposition genetic testing.

Supplementary Table 2 List of genes included in different subpanels and respective versions.

Supplementary Table 3 Number of patients tested on each sub-panel (Panel name/no of genes; genes listed in Supplementary Table 2).

Supplementary Table 4 List of all patients tested and genetic variants (ACMG 1, 2, and 3) identified in genes included on the panel.

Supplementary Table 5 Novel Pathogenic Variants (PVs) identified in the study.

Supplementary Table 6 Copy Number Variants identified in this study using our NGS algorithm and validation using alternate techniques.

Supplementary Table 7 Number of patients with each cancer type positive for a pathogenic variant in genes across all sub-panels.
