# Supplementary material for: Analysis of Sequence and Copy Number Variants in Canadian Patient Cohort With Familial Cancer Syndromes Using a Unique Next Generation Sequencing Based Approach
Source: Front Genet. 2021 Jul 13;12:698595. doi: 10.3389/fgene.2021.698595 (PMC8314385; doi:10.3389/fgene.2021.698595)
Supplement: Supplementary Table 1 — Guidelines for referral for hereditary cancer predisposition genetic testing. [file Data_Sheet_1.zip › Supplementary files/Supplementary Table S1.docx]

**Supplementary Table S1:** Guidelines for referral for hereditary cancer predisposition genetic testing

**RISK CATEGORIES FOR INDIVIDUALS ELIGIBLE FOR SCREENING FOR A GENETIC SUSCEPTIBILITY TO BREAST OR OVARIAN CANCERS**

**Testing for Affected Individuals with Breast or Ovarian Cancer**

**At least one case of cancer:**

1. Ashkenazi Jewish and breast cancer <50 years, or ovarian cancer at any age.

*Note: testing limited to ethnic specific mutations, unless other criteria given in this list are met.*

2. Breast cancer <35 years of age.

3. Male breast cancer.

4. Invasive serous ovarian cancer at any age.

**At least 2 cases of cancer on the same side of the family:**

5. Breast cancer <60 years, and a first or second-degree relative with ovarian cancer or male breast cancer.

6. Breast and ovarian cancer in the same individual, or bilateral breast cancer with the first case <50 years.

7. Two cases of breast cancer, both <50 years, in first or second-degree relatives.

8. Two cases of ovarian cancer, any age, in first or second-degree relatives.

9. Ashkenazi Jewish and breast cancer at any age, and any family history of breast or ovarian cancer.

*Note: testing limited to ethnic specific mutations, unless other criteria given in this list are met.*

**At least 3 cases of cancer on the same side of the family:**

10. Three or more cases of breast or ovarian cancer at any age.

***Testing for Unaffected Individuals (this should be done only if affected individuals are unavailable e.g. deceased)***

11. Relative of individual with known BRCA1 or BRCA2 mutation.

*Note: specific family mutation only tested.*

12. Ashkenazi Jewish and first or second-degree relative of individual with:

breast cancer <50 years, or-ovarian cancer at any age, or-male breast cancer, or-breast cancer, any age, with positive family history of breast or ovarian cancer

*Note: testing limited to ethnic specific mutations, unless meet other criteria*

13. A pedigree strongly suggestive of hereditary breast/ovarian cancer, i.e. risk of carrying a mutation for the individual being tested is >10%.

**RISK CATEGORIES FOR INDIVIDUALS ELIGIBLE FOR SCREENING FOR A GENETIC**

**SUSCEPTIBILITY TO COLON CANCER**

**Testing for Hereditary Non-Polyposis Colon Cancer (HNPCC)**

If a tumour sample is unavailable, germline testing may proceed on the youngest, living, affected individual from families meeting criteria 1 & 2 ONLY.

1. Affected and unaffected individuals from families with a known HNPCC causing mutation.

2. Affected individual from Amsterdam I and II families. Family must meet all of the following criteria:

- Three affected relatives with any combination of colorectal, endometrial,small bowel,ureter, transitional cell kidney cancer (urothelial), sebaceous adenoma/carcinoma and/or keratoacanthoma.

- One should be a first-degree relative of the other two.

- At least 2 successive generations should be affected.

- At least 1 diagnosis must be before age 50.

- Tumour type should be confirmed by review of pathology or other medical records.

3. Affected individuals from families with:

- Three affected individuals, one with colorectal cancer, and the other two with any combination of: colorectal, endometrial, small bowel, ureter, sebaceous adenoma/carcinoma, ovarian, pancreatic, kidney (transitional cell cancer only), gastric, primary brain or primary hepatobiliary cancer.

- Two of the three family members must be in a first-degree relationship.

- At least one diagnosis under the age of 50.

- FAP should be excluded.

- Tumors should be verified by pathological examination.

4. Individual affected with CRC and a second primary HNPCC-associated cancer (as listed in #3). This includes synchronous and metachronous colorectal cancers. At least one primary cancer must be diagnosed under age 55. Families are eligible with or without family history of HNPCC-associated cancer, and tumours should be verified by pathological examination.

5. Individual diagnosed with CRC under the age of 35. Families are eligible with or without family history of HNPCC-associated cancer, and tumours should be verified by pathological examination.

6. One case of CRC<50, with a 1st or 2nd degree relative with one of the following HNPCC-related cancers diagnosed <50; colorectal, endometrial, small bowel, ureter, urothelial, sebaceous adenoma/carcinoma or keratoacanthoma.

7. Individuals with immunodeficient tumours (regardless of family history) as follows

- MSH2 deficient tumour +/- MSH6 deficiency (sequence and MLPA of MSH2 gene only)

- MSH6 (only) deficient tumour (sequence and MLPA of MSH6 gene only)

- MLH1 deficient tumour in individual < age 60 (sequence and MLPA of MLH1 gene only)

**Testing for Familial Adenomatous Polyposis (FAP)**

Families eligible for testing:

1. Affected and unaffected individuals from families with a known FAP causing mutation.

2. Individuals with clinical confirmed FAP (100 or more adenomas).

3. Individuals with putative attenuated FAP, that is, 10 or more histologically confirmed adenomas. Cumulative pathology and endoscopy reports are required to confirm that the total number and histology are appropriate. A referral with less than 10 adenomas (including hyperplastic polyps) will be excluded.
