# Supplementary material for: Analysis of Sequence and Copy Number Variants in Canadian Patient Cohort With Familial Cancer Syndromes Using a Unique Next Generation Sequencing Based Approach
Source: Front Genet. 2021 Jul 13;12:698595. doi: 10.3389/fgene.2021.698595 (PMC8314385; doi:10.3389/fgene.2021.698595)
Supplement: Supplementary Table 1 — Guidelines for referral for hereditary cancer predisposition genetic testing. [file Data_Sheet_1.zip › Supplementary files/Supplementary Table S2.docx]

**Supplementary Table S2** : List of genes included in different subpanels and respective versions
