# Supplementary material for: Analysis of Sequence and Copy Number Variants in Canadian Patient Cohort With Familial Cancer Syndromes Using a Unique Next Generation Sequencing Based Approach
Source: Front Genet. 2021 Jul 13;12:698595. doi: 10.3389/fgene.2021.698595 (PMC8314385; doi:10.3389/fgene.2021.698595)
Supplement: Supplementary Table 1 — Guidelines for referral for hereditary cancer predisposition genetic testing. [file Data_Sheet_1.zip › Supplementary files/Supplementary Table S3.docx]

**Supplementary Table S3: Number** of patients tested on each sub-panel (Panel name /no of genes; genes listed in Supplementary Table S2)

|  | Total patients | Percent of total patients | No. of patients with a pathogenic variant | Percent of patients within row with a pathogenic variant |
| --- | --- | --- | --- | --- |
| HCP Core | **432** | **15** | **56** | **13.1** |
| *Version 1 (16 genes)* | 377 | 13.0 | 48 | 12.9 |
| *Version 2 (16 genes)** | 55 | 1.9 | 8 | 14.5 |
| Breast & ovarian cancer panel | **1021** | **36** | **139** | **13.6** |
| *Version 1 (20 genes)* | 274 | 9.5 | 37 | 13.5 |
| *Version 2 (20 genes)** | 578 | 20.1 | 79 | 13.7 |
| *Version 3 (24 genes)** | 169 | 5.9 | 23 | 13.6 |
| Comprehensive Panel | **1308** | **46** | **212** | **16.2** |
| *Version 1 (26 genes)* | 435 | 15.2 | 79 | 18.2 |
| *Version 2 (31 genes)** | 667 | 23.2 | 97 | 14.5 |
| *Version 3 (38 genes)** | 206 | 7.2 | 36 | 17.5 |
| Gastric & colorectal cancer panel | **109** | **4** | **24** | **22.0** |
| *Version 1 (16 genes)* | 28 | 1.0 | 9 | 32.1 |
| *Version 2 (21 genes )** | 51 | 1.8 | 10 | 19.6 |
| *Version 3 (24 genes)** | 30 | 1.0 | 5 | 16.7 |
| Total | 2870 | 100 | 431 | 15.0 |

*** Promoter analysis included for PTEN and APC**
