# Supplementary material for: Analysis of Sequence and Copy Number Variants in Canadian Patient Cohort With Familial Cancer Syndromes Using a Unique Next Generation Sequencing Based Approach
Source: Front Genet. 2021 Jul 13;12:698595. doi: 10.3389/fgene.2021.698595 (PMC8314385; doi:10.3389/fgene.2021.698595)
Supplement: Supplementary Table 1 — Guidelines for referral for hereditary cancer predisposition genetic testing. [file Data_Sheet_1.zip › Supplementary files/Supplementary Table S5.docx]

**Supplementary Table S5:** Novel Pathogenic Variants (PVs) identified in the study

| S. No | Novel pathogenic variants (ACMG 1 and 2)  (absent from Clinvar and literature) | Proband's Cancer Type | Types of cancer observed in the Proband's family |
| --- | --- | --- | --- |
| 1 | APC:c.1-?_165+?del | Polyps | Colorectum; Other |
| 2 | APC:c.1958+241_4457del | Colorectum | NA |
| 3 | ATM:c.2467-1G>T | Breast | Breast; Ovary; Prostate; Colorectum; Hematological |
| 4 | ATM:c.3746+1G>T | Lung /Retino blastoma | unspecified |
| 5 | ATM:c.57_58dup,p.(Ala20Glufs*15) | Ovary | NA |
| 6 | ATM:c.7736_7737insC,p.(Arg2579Serfs*7) | Polyps | Ovary; Colorectum; Polyps |
| 7 | BRCA1:c.1195_1196del,p.(His399*) | Breast; Ovary | NA |
| 8 | BRCA1:c.300delG,p.(Glu100Aspfs*19) | Ovary | NA |
| 9 | BRCA1:c.4276delT,p.(Ser1426Leufs*8) | Breast; Ovary | Ovary |
| 10 | BRCA2:c.871_884del,p.(Leu291Ilefs*2) | Breast | Breast; Ovary |
| 11 | BRIP1:c.628-1G>T | Colorectum | Colorectum; Sarcoma; Other |
| 12 | MLH1:c.1872delC,p.(Tyr625Ilefs*12) | Colorectum | NA |
| 13 | MLH1:c.843dupA,p.(Ala282Serfs*25) | Uterus | Gastric; Hematological; Other |
| 14 | MSH6:c.(?_-21)_(*21_?)del; MSH2:c.(?_-21)_(*21_?)del | Polyps | OtherGI; Melanoma |
| 15 | MSH6:c.2966dupA,p.(Asn989Lysfs*16) | Colorectum | Colorectum; OtherGI; Polyps; Melanoma; Hematological |
| 16 | PALB2:c.2328delC,p.(Phe776Leufs*75) | Pancreas | Uterus; Gastric |
| 17 | PMS2:c.2279delC,p.(Pro760Glnfs*8) | Polyps | Ovary; Prostate; Uterus; Colorectum; Brain |
| 18 | PMS2:c.546delC,p.(Met184Trpfs*17) | Polyps | Other |
| 19 | RAD51C:c.571+2T>C | Ovary | Ovary; Colorectum; Other |
| 20 | RAD51C:c.572-?_1131+?del | Ovary | Breast; Prostate; Other |
| 21 | RAD51D:c.(?_-21)_(*21_?)del | Breast | Breast; Prostate; OtherGI; Melanoma; Brain; Other |
| 22 | SMAD4:c.1425_1428del,p.(Gly476Glufs*2) | Polyps | Colorectum |
| 23 | TP53:c.580_581delinsA,p.(Leu194Ilefs*53) | Ovary | Colorectum; Gastric |
