# Supplementary material for: Analysis of Sequence and Copy Number Variants in Canadian Patient Cohort With Familial Cancer Syndromes Using a Unique Next Generation Sequencing Based Approach
Source: Front Genet. 2021 Jul 13;12:698595. doi: 10.3389/fgene.2021.698595 (PMC8314385; doi:10.3389/fgene.2021.698595)

Supplementary Table S6: Copy Number Variants identified in this study using our NGS algorithm and validation using alternate techniques .

S No	Variant	Number of patients positive for this variant	ACMG category#	Alternate technique used to confirm CNV##	Confirmation results using alternate techniques shown below ###
1	**APC:c.1-?_165+?del ***	1	2	MLPA	Figure (i)
2	APC:c.1958+241_4457del**	1	1	LR-PCR / Sanger	Validation shown in previous study [17]
3	ATM:c.(?_-21)_(*21_?)del (15%)	1	1	MLPA	Figure (ii)
4	**ATM:c.(662+21_663-21)_(9171+21_9172-21)dup***	1	3	MLPA	Figure (iii)
5	**ATM:c.(8850+21_8851-21)_(*21_?)[4]***	1	3	MLPA	Figure (iv)
6	BARD1:c.(?_-20)_(*20_?)del	1	1	Not Analysed	-
7	BRCA1:c.(?_-21)_(80+21_81-21)[3]	1	3	MLPA	Figure (v)
8	BRCA1:c.(?_-21)_(80+21_81-21)del	1	1	MLPA	Figure (vi)
9	BRCA1:c.(4185+21_4186-21)_(4357+21_4358-21)dup	1	1	MLPA	Figure (vii)
10	BRCA1:c.(4357+21_4358-21)_(4484+21_4485-21)del	1	1	MLPA	Figure (viii)
11	BRCA1:c.(4986+21_4987-21)_(5074+21_5075-21)del	2	1	MLPA	Figure (ix)
12	BRCA1:c.(5277+21_5278-21)_(5592+21_*21_?)del	1	1	MLPA	Figure (x)
13	BRCA1:c.(5332+21_5333-21)_(5406+21_5407-21)del	1	1	MLPA	Figure (xi)
14	BRCA1:c.(5406+21_5407-21)_(*21_?)del	1	1	MLPA	Figure (xii)
15	BRCA2:c.(?_-21)_(67+21_68-21)del	1	2	MLPA	Figure (xiii)
16	BRCA2:c.(8487+21_8488-21)_(8632+21_8633-21)del	1	1	MLPA	Figure (xiv)
17	CHEK2:c.(1461+21_1462-21)_(*21_?)del	1	1	MLPA	Figure (xv)
18	CHEK2:c.(319+21_320-21)_(592+21_593-21)dup	2	2	MLPA	Figure (xvi)
19	CHEK2:c.(908+21_909-21)_(1095+21_1096-21)del	3	1	MLPA	Figure (xvii)
20	MLH1:c.(116+21_117-21)_(545+21_546-21)del	2	1	MLPA	Figure (xviii)

21	MSH2:c.(?_-21)_(*21_?)del	1	1	MLPA	Figure (xix)
22	MSH2:c.(366+21_367-21)_(1076+21_1077-21)del	1	1	MLPA	Figure (xx)
23	MSH6:c.(?_-21)_(*21_?)del	1	1	MLPA	Figure (xxi)
24	MSH6:c.(?_-21)_(260+21_261-21)del	1	1	MLPA	Figure (xxii)
25	PALB2:c.(2586+21_2587-21)_(2748+21_2749-21)del	1	1	MLPA	Figure (xxiii)
26	PMS2:c.(2006+21_2007-21)_(*21_?)dup	1	3	MLPA	Figure (xxiv)
27	PMS2:c.(2445+21_2446-21)_(*21_?)del	1	1	MLPA	Figure (xxv)
28	PMS2:c.(537+21_538-21)_(903+21_904-21)del	1	1	MLPA	Figure (xxvi)
29	**RAD51C:c.572-?_1131+?del ***	1	1	MLPA	Figure (xxvii)
30	RAD51C:c.(837+21_838-21)_(965+21_966-21)dup	1	1	MLPA	Figure (xxviii)
31	**RAD51D:c.(?_-21)_(*21_?)del ***	1	1	MLPA	Figure (xxix)
32	POLE:c.5959_5960ins198,p.(Leu1986_Pro1987ins66) **	1	3	LR-PCR / Sanger	Figure (xxx)
33	PTEN:c.80-958_80-60del *	1	3	LR-PCR / Sanger	Figure (xxxi)

*Copy number variants not reported elsewhere in literature and Clinvar

** CNVs reported by our lab previously (including validation)

# ACMG category 1: Pathogenic

ACMG category 2: Likely pathogenic

ACMG category 3: Unknown Significance

MLPA: Multiplex ligation-dependent probe amplification ;LR-PCR :Long Range PCR

<u>### Confirmation results using alternate techniques</u>

Figure (i) : MLPA results showing APC:c.1-?_165+?del *

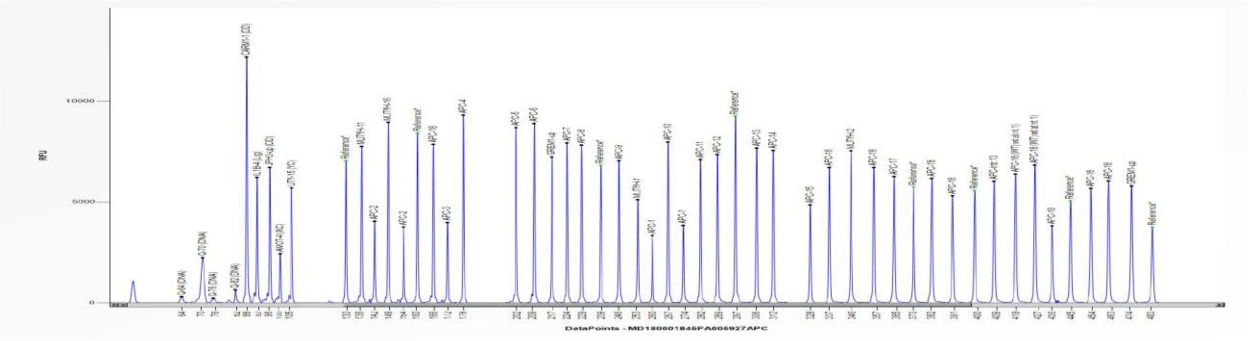

D [nt]	Gene-Exon	Chr.band	hg18 loc.	Height	Area	Ratio[H]	Stdev	[REF]	[Sam]	Width	d[nt]
260	APC-1	05q22.2	05-112.071072	3338	24071	***0.5***	0.08	<<*	?	52	0.3
274	APC-1	05q22.2	05-112.071434	3840	29848	***0.5***	0.02	<<*	?	54	0.5
142	APC-2	05q22.2	05-112.101127	4046	28531	***0.51***	0.04	<<*	?	33	**1.0**
154	APC-2	05q22.2	05-112.101435	3759	27170	***0.5***	0.03	<<*	?	35	-1.1
172	APC-3	05q22.2	05-112.101975	3983	27101	***0.5***	0.06	<<*	?	32	-0.8
178	APC-4	05q22.2	05-112.118477	9313	60775	*0.98*	0.03	=	=	37	-0.7
202	APC-5	05q22.2	05-112.129936	8690	59954	*0.97*	0.04	=	=	45	0.1
209	APC-6	05q22.2	05-112.130843	8896	62561	*0.96*	0.03	=	=	56	0.0
224	APC-7	05q22.2	05-112.139223	7932	56095	*0.97*	0.04	=	=	50	0.0
229	APC-8	05q22.2	05-112.144431	7826	54610	*0.97*	0.04	=	=	48	0.3
246	APC-9	05q22.2	05-112.156040	7051	51566	*1*	0.04	=	=	44	0.4
267	APC-10	05q22.2	05-112.164867	7978	59341	*1.04*	0.04	=	=	62	**0.5**
282	APC-11	05q22.2	05-112.179090	7105	54274	*0.98*	0.05	=	=	65	**0.7**
289	APC-12	05q22.2	05-112.182873	7356	57149	*0.98*	0.04	=	=	78	0.1
306	APC-13	05q22.2	05-112.185511	7679	60135	*0.95*	0.04	=	=	61	0.0
409	APC-intr.13	05q22.2	05-112.186901	6032	57337	*0.96*	0.07	=	=	78	-0.1
312	APC-14	05q22.2	05-112.190704	7560	59633	*1.01*	0.05	=	=	55	-0.3
328	APC-15	05q22.2	05-112.191531	4857	38240	*0.85*	**0.16**	=	=	46	-0.1
337	APC-16	05q22.2	05-112.192470	6710	53319	*0.96*	0.04	=	=	40	-0.5
365	APC-17	05q22.2	05-112.198622	6265	55425	*0.94*	0.08	=	=	80	**0.6**
382	APC-18	05q22.2	05-112.201341	6171	56883	*0.97*	0.03	=	=	50	0.2
419	APC-18 (WT) w	05q22.2	05-112.202335	6386	61870	*0.95*	0.04	=	=	57	-0.2
427	APC-18 (WT) w	05q22.2	05-112.203069	6832	67649	*0.97*	0.05	=	=	83	-0.7
357	APC-18	05q22.2	05-112.204006	6711	59169	*0.98*	0.04	=	=	86	0.4
454	APC-18	05q22.2	05-112.204713	5675	58089	*0.98*	0.03	=	=	58	0.5
166	APC-18	05q22.2	05-112.205396	7863	53878	*0.99*	0.07	=	=	48	-0.5
463	APC-18	05q22.2	05-112.206115	6050	60772	*1*	0.06	=	=	68	-0.3
436	APC-18	05q22.2	05-112.206814	3811	37131	*0.88*	**0.23**	=	=	40	-0.7
391	APC-18	05q22.2	05-112.207595	5308	48217	*1*	0.05	=	=	63	0.3
217	GREM1-up	15q13.3	15-030.789036	7230	50146	*0.98*	0.03	=	=	56	0.5
474	GREM1-up	15q13.3	15-030.791889	5793	62242	*1.03*	0.06	=	=	81	-0.9
400	Reference*	01p31.3	01-065.848260	5573	51297	*1*	0.03	=	=	83	0.0
483	Reference*	04q12	04-055.298084	3757	41917	*1.01*	0.08	=	=	69	-0.4
130	Reference*	05q31.1	05-132.037607	7051	47730	*1.01*	0.04	=	=	44	**0.8**
445	Reference*	06q21	06-108.321498	5056	50245	*1*	0.04	=	=	65	0.0
297	Reference*	07q21.2	07-091.680512	9273	72767	*0.97*	0.06	=	=	75	0.3
238	Reference*	08q22.2	08-100.781024	6830	47880	*1.02*	0.03	=	=	43	0.5
373	Reference*	16q13	16-055.495805	5751	50390	*0.94*	0.09	=	=	51	-0.2
160	Reference*	17p13.1	17-007.066732	8420	58275	*0.99*	0.04	=	=	54	-0.5
**Median value all probe values:**				**6711**	**54698**	**0.97**	**0.04**			**56**	**0.02**

Figure (ii): Figure (i) : MLPA results showing : ATM:c.(?_-21)_(*21_?)del (15%)

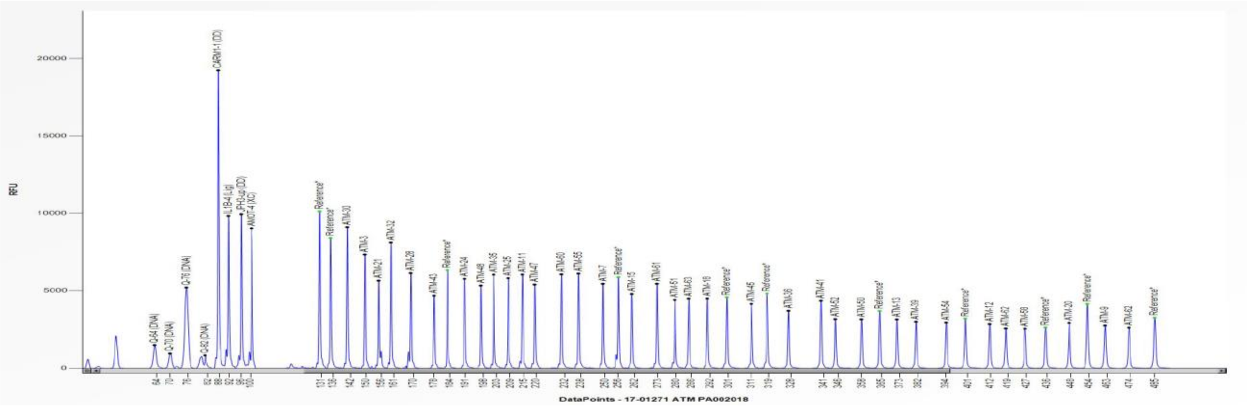

D [nt]	Gene-Exon	Chr.band	hg18 loc.	Height	Area	Ratio^{H}	Stdev	[REF]	[Sam]	Width	d[nt]
150	ATM-3	11q22.3	11-107.603703	7341	50328	*0.88*	0.04	=	=	39	0.1
250	ATM-7	11q22.3	11-107.620815	5453	42290	*0.78*	0.04	<<	=	53	0.0
463	ATM-9	11q22.3	11-107.624892	2761	31259	*0.73*	0.04	<<	=	59	0.1
215	ATM-11	11q22.3	11-107.627935	6053	46051	*0.83*	0.04	<<	=	50	-0.1
412	ATM-12	11q22.3	11-107.628759	2854	28633	*0.81*	0.04	<<	=	60	0.0
373	ATM-13	11q22.3	11-107.629847	3150	30118	*0.77*	0.04	<<	=	84	0.0
262	ATM-15	11q22.3	11-107.633459	4796	38245	*0.75*	0.04	<<	=	59	-0.1
292	ATM-18	11q22.3	11-107.644411	4498	37772	*0.76*	0.04	<<	=	67	0.1
448	ATM-20	11q22.3	11-107.647275	2929	31491	*0.72*	0.04	<<	=	59	0.0
156	ATM-21	11q22.3	11-107.648427	5655	45169	*0.86*	0.05	=	=	56	0.0
191	ATM-24	11q22.3	11-107.657007	5773	41644	*0.83*	0.04	<<	=	46	-0.1
209	ATM-25	11q22.3	11-107.658636	5817	43254	*0.81*	0.04	<<	=	52	-0.1
170	ATM-28	11q22.3	11-107.664991	6149	49284	*0.82*	0.05	=	=	57	0.1
142	ATM-30	11q22.3	11-107.668598	9106	63480	*0.87*	0.05	=	=	61	0.0
161	ATM-32	11q22.3	11-107.670949	8121	57862	*0.86*	0.04	<<	=	57	0.0
203	ATM-35	11q22.3	11-107.677594	6049	44609	*0.84*	0.04	<<	=	62	0.0
328	ATM-36	11q22.3	11-107.678850	3710	32664	*0.8*	0.04	<<	=	85	0.0
382	ATM-39	11q22.3	11-107.686031	3003	29370	*0.75*	0.04	<<	=	75	0.0
341	ATM-41	11q22.3	11-107.691766	4359	38909	*0.75*	0.04	<<	=	60	0.0
178	ATM-43	11q22.3	11-107.693358	4686	33483	*0.83*	0.04	<<	=	47	0.0
311	ATM-45	11q22.3	11-107.697268	4157	36027	*0.8*	0.04	<<	=	66	0.1
220	ATM-47	11q22.3	11-107.702093	5403	41649	*0.82*	0.05	<<	=	48	0.1
198	ATM-48	11q22.3	11-107.703616	5338	38132	*0.83*	0.04	<<	=	55	0.0
358	ATM-50	11q22.3	11-107.706170	3158	29438	*0.81*	0.05	<<	=	56	0.0
280	ATM-51	11q22.3	11-107.707413	4412	37008	*0.77*	0.03	<<	=	62	0.0
346	ATM-52	11q22.3	11-107.707867	3168	28637	*0.79*	0.04	<<	=	59	0.0
394	ATM-54	11q22.3	11-107.709834	2947	30167	*0.82*	0.04	<<	=	70	0.1
238	ATM-55	11q22.3	11-107.710889	6116	47908	*0.8*	0.04	<<	=	73	0.0
427	ATM-58	11q22.3	11-107.721746	2553	26248	*0.77*	0.04	<<	=	61	0.0
232	ATM-60	11q22.3	11-107.729703	6068	45896	*0.82*	0.04	<<	=	69	0.0
273	ATM-61	11q22.3	11-107.731288	5455	43748	*0.8*	0.04	<<	=	79	0.1
474	ATM-62	11q22.3	11-107.739228	2619	29946	*0.75*	0.04	<<	=	72	0.0
419	ATM-62	11q22.3	11-107.739925	2568	26518	*0.75*	0.05	<<	=	57	0.0
286	ATM-63	11q22.3	11-107.741279	4492	36613	*0.85*	0.04	<<	=	62	0.1
436	Reference*	01q23.3	01-159.406366	2632	27860	*0.91*	0.04	=	=	53	0.0
319	Reference*	03q13.2	03-113.667629	4830	44045	*1.03*	0.05	=	=	66	0.0
131	Reference*	03q21.3	03-129.999515	10133	72379	*1.08*	0.06	=	=	52	-0.1
256	Reference*	04q25	04-110.941155	5883	49339	*1.01*	0.05	=	=	51	0.0
365	Reference*	05q33.3	05-158.137017	3698	34339	*0.99*	0.05	=	=	68	0.0
301	Reference*	06q22.33	06-129.511860	4582	38757	*0.96*	0.04	=	=	71	0.0
136	Reference*	07p14.3	07-030.974908	8411	60800	*1.06*	0.05	=	=	53	-0.1
401	Reference*	08q12.2	08-061.913286	3205	33704	*0.97*	0.05	=	=	72	0.0
184	Reference*	09p24.2	09-002.644106	6341	43935	*1.01*	0.05	=	=	55	0.0
454	Reference*	13q13.1	13-031.879392	4153	44316	*0.93*	0.04	=	=	61	0.0
485	Reference*	18q21.1	18-045.630186	3260	37819	*0.93*	0.05	=	=	74	0.0
**Median value all probe values:**				**4582**	**38245**	**0.82**	**0.04**			**60**	**-0.01**

Figure (iii) MLPA results showing ATM:c.(662+21_663-21)_(9171+21_9172-21)dup*

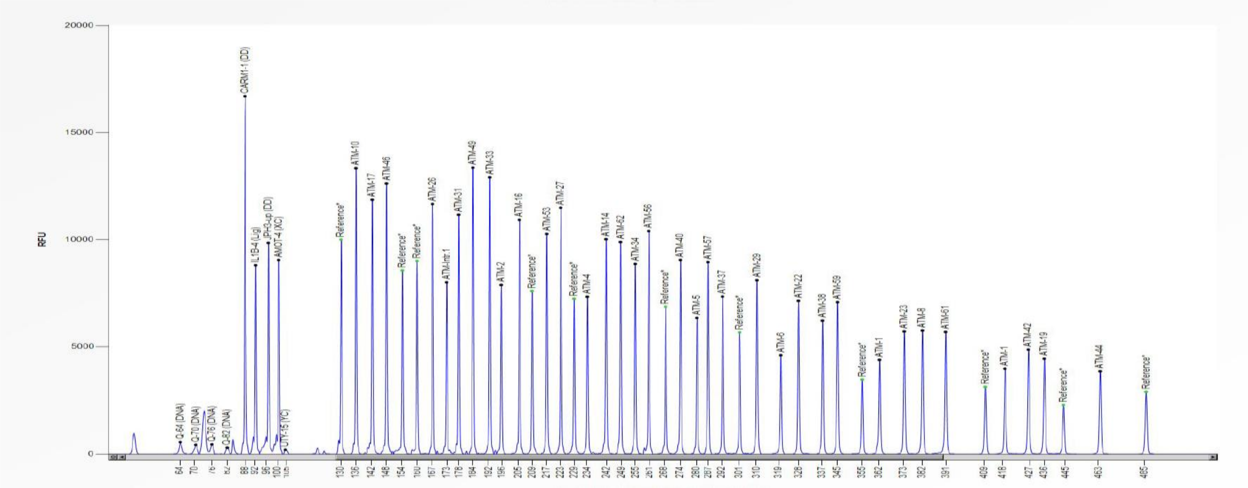

D [nt]	Gene-Exon	Chr.band	hg18 loc.	Height	Area	Ratio [H]	Stdev	[REF]	[Sam]	Width	d[nt]
418	ATM-1	11q22.3	11-107.598830	3983	38699	*1.05*	0.04	=	=	71	0.1
362	ATM-1	11q22.3	11-107.598966	4397	41378	*1.1*	0.04	=	=	61	0.1
173	ATM-Intr.1	11q22.3	11-107.600753	8016	56596	*0.97*	0.06	=	=	54	0.0
196	ATM-2	11q22.3	11-107.603560	7894	54032	*1.03*	0.03	=	=	37	0.0
234	ATM-4	11q22.3	11-107.605158	7342	54118	*0.97*	0.03	=	=	42	0.0
280	ATM-5	11q22.3	11-107.611683	6355	48787	*1.03*	0.05	=	=	51	0.0
319	ATM-6	11q22.3	11-107.619924	4614	38466	*1.07*	0.04	=	=	56	-0.1
382	ATM-8	11q22.3	11-107.622916	5768	53641	***1.65***	0.07	>>*	=	59	0.0
136	ATM-10	11q22.3	11-107.626777	13344	89516	***1.41***	0.08	>>*	=	50	0.0
242	ATM-14	11q22.3	11-107.632194	10030	75631	***1.44***	0.06	>>*	=	62	0.0
205	ATM-16	11q22.3	11-107.634921	10932	77750	***1.49***	0.06	>>*	=	69	0.0
142	ATM-17	11q22.3	11-107.643137	11874	86943	***1.44***	0.04	>>*	=	75	-0.1
436	ATM-19	11q22.3	11-107.646985	4450	44021	***1.53***	0.07	>>*	=	47	0.1
328	ATM-22	11q22.3	11-107.648710	7153	56664	***1.43***	0.05	>>*	=	52	-0.1
373	ATM-23	11q22.3	11-107.655436	5726	51815	***1.45***	0.08	>>*	=	60	0.0
167	ATM-26	11q22.3	11-107.660224	11674	77388	***1.48***	0.05	>>*	=	36	0.0
223	ATM-27	11q22.3	11-107.663610	11497	81407	***1.59***	0.06	>>*	=	51	0.0
310	ATM-29	11q22.3	11-107.665703	8116	67377	***1.59***	0.06	>>*	=	45	0.0
178	ATM-31	11q22.3	11-107.669339	11172	76042	***1.54***	0.05	>>*	=	47	0.0
192	ATM-33	11q22.3	11-107.673233	12919	89093	***1.47***	0.06	>>*	=	46	-0.1
255	ATM-34	11q22.3	11-107.675638	8876	66397	***1.44***	0.07	>>*	=	50	-0.1
292	ATM-37	11q22.3	11-107.680715	7351	58100	***1.41***	0.06	>>*	=	58	0.0
337	ATM-38	11q22.3	11-107.683830	6227	51994	***1.43***	0.05	>>*	=	50	0.0
274	ATM-40	11q22.3	11-107.688294	9059	68696	***1.44***	0.05	>>*	=	55	0.0
427	ATM-42	11q22.3	11-107.691970	4875	49226	***1.57***	0.07	>>*	=	68	0.0
463	ATM-44	11q22.3	11-107.695883	3865	39452	***1.58***	**0.14**	>>*	=	56	0.0
148	ATM-46	11q22.3	11-107.701285	12629	88096	***1.41***	0.04	>>*	=	51	-0.1
184	ATM-49	11q22.3	11-107.705086	13369	90938	***1.49***	0.07	>>*	=	37	0.0
217	ATM-53	11q22.3	11-107.708696	10277	74261	***1.4***	0.05	>>*	=	49	-0.1
261	ATM-56	11q22.3	11-107.711788	10409	74869	***1.5***	0.05	>>*	=	32	0.0
287	ATM-57	11q22.3	11-107.719162	8960	67915	***1.47***	0.04	>>*	=	35	0.0
345	ATM-59	11q22.3	11-107.723225	7092	61343	***1.53***	0.06	>>*	=	56	-0.1
391	ATM-61	11q22.3	11-107.730735	5703	54661	***1.54***	0.08	>>*	=	52	0.0
249	ATM-62	11q22.3	11-107.740982	9898	74236	***1.48***	0.06	>>*	=	57	0.0
154	Reference*	02q12.3	02-108.906225	8571	63585	*1.03*	0.03	=	=	66	0.0
130	Reference*	03q21.3	03-129.999519	10012	69634	*0.99*	0.03	=	=	41	0.0
268	Reference*	05p15.2	05-009.490899	6876	52651	*1.01*	0.03	=	=	62	0.0
160	Reference*	07q21.3	07-093.873480	9012	60022	*0.98*	0.03	=	=	32	0.0
355	Reference*	08q22.3	08-105.578750	3479	30554	*1.01*	0.04	=	=	51	0.0
445	Reference*	13q14.3	13-050.428652	2288	23855	*1.06*	0.06	=	=	55	0.0
229	Reference*	15q25.2	15-082.497791	7251	54672	*0.99*	0.05	=	=	63	-0.1
301	Reference*	16q13	16-055.490937	5684	45357	*0.98*	0.03	=	=	59	0.0
409	Reference*	17q21.2	17-037.637569	3135	30764	*0.96*	0.03	=	=	47	0.0
485	Reference*	18q21.1	18-045.630186	2903	31119	*1.12*	0.08	=	=	53	-0.1
209	Reference*	20q11.23	20-034.978521	7612	54580	*0.96*	0.03	=	=	51	0.0
**Median value all probe values:**				**7612**	**56664**	**1.43***	**0.05**			**52**	**-0.02**

Figure (iv) : MLPA results showing ATM:c.(8850+21_8851-21)_(*21_?)[4]*

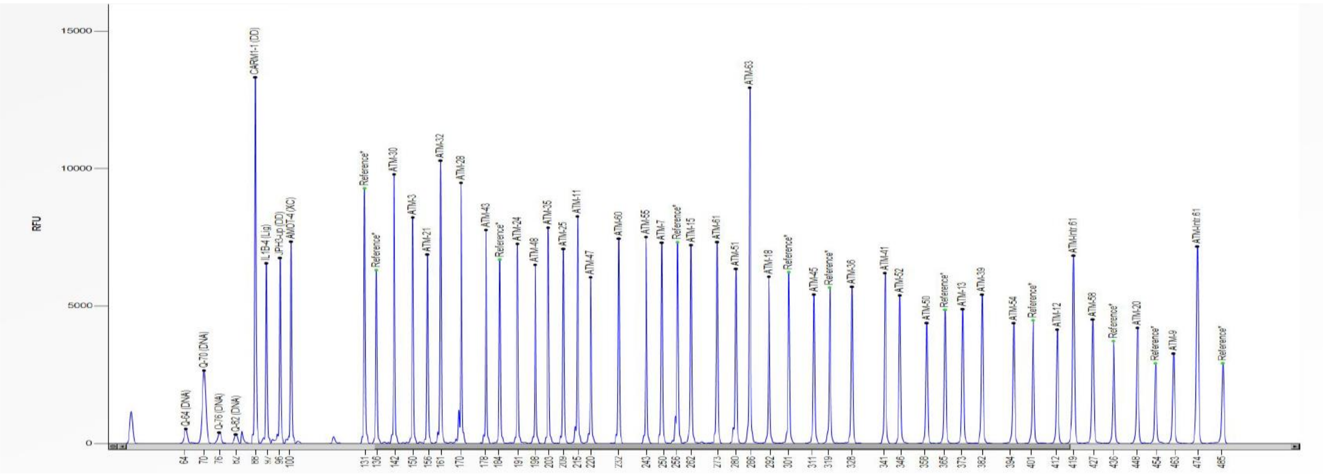

D [nt]	Gene-Exon	Chr.band	hg18 loc.	Height	Area	Ratio^{H}	Stdev	[REF]	[Sam]	Width	d[nt]
150	ATM-3	11q22.3	11-107.603703	8226	56460	1.01	0.05	=	=	44	-0.1
250	ATM-7	11q22.3	11-107.620815	7307	55967	1.01	0.04	=	=	54	0.0
463	ATM-9	11q22.3	11-107.624892	3272	37514	1.03	0.04	=	=	76	0.0
215	ATM-11	11q22.3	11-107.627935	8264	63451	1.04	0.04	=	=	60	0.0
412	ATM-12	11q22.3	11-107.628759	4145	41533	0.99	0.04	=	=	57	-0.1
373	ATM-13	11q22.3	11-107.629847	4889	46482	1.01	0.03	=	=	58	0.1
262	ATM-15	11q22.3	11-107.633459	7220	59036	1.02	0.04	=	=	64	0.0
292	ATM-18	11q22.3	11-107.644411	6070	50350	1.04	0.05	=	=	62	0.0
448	ATM-20	11q22.3	11-107.647275	4208	45565	1.01	0.04	=	=	56	-0.1
156	ATM-21	11q22.3	11-107.648427	6878	47566	1.08	**0.11**	=	=	44	0.0
191	ATM-24	11q22.3	11-107.657007	7265	52076	1.02	0.05	=	=	45	0.0
209	ATM-25	11q22.3	11-107.658636	7081	52129	0.99	0.03	=	=	44	0.0
170	ATM-28	11q22.3	11-107.664991	9486	73927	1.11	0.09	=	=	53	0.0
142	ATM-30	11q22.3	11-107.668598	9794	69185	1	0.03	=	=	45	0.0
161	ATM-32	11q22.3	11-107.670949	10294	72826	1.07	0.05	=	=	57	0.0
203	ATM-35	11q22.3	11-107.677594	7855	56283	1.05	0.04	=	=	45	0.1
328	ATM-36	11q22.3	11-107.678850	5703	49786	1.08	**0.15**	=	=	50	0.0
382	ATM-39	11q22.3	11-107.686031	5418	52993	1.04	0.05	=	=	49	0.1
341	ATM-41	11q22.3	11-107.691766	6201	54748	1.05	0.04	=	=	51	0.1
178	ATM-43	11q22.3	11-107.693358	7769	54993	1.01	0.03	=	=	47	0.0
311	ATM-45	11q22.3	11-107.697268	5420	46493	1.03	0.05	=	=	67	0.0
220	ATM-47	11q22.3	11-107.702093	6049	46586	0.98	0.05	=	=	55	0.0
198	ATM-48	11q22.3	11-107.703616	6502	47349	1.01	0.03	=	=	53	0.1
358	ATM-50	11q22.3	11-107.706170	4385	40562	0.98	0.06	=	=	65	0.0
280	ATM-51	11q22.3	11-107.707413	6354	53479	1.04	0.08	=	=	57	0.0
346	ATM-52	11q22.3	11-107.707867	5390	48272	0.99	0.04	=	=	61	0.0
394	ATM-54	11q22.3	11-107.709834	4380	44002	1.04	0.05	=	=	68	0.0
243	ATM-55	11q22.3	11-107.710889	7514	56834	1.05	0.06	=	=	45	0.0
427	ATM-58	11q22.3	11-107.721746	4514	47984	1.02	0.03	=	=	80	-0.1
232	ATM-60	11q22.3	11-107.729703	7455	55086	1.02	0.03	=	=	37	-0.1
273	ATM-61	11q22.3	11-107.731288	7330	58165	1.01	0.05	=	=	42	-0.1
474	ATM-Intr.61	11q22.3	11-107.739228	7169	81789	**2.43**	**0.15**	**>>***	=	67	0.0
419	ATM-Intr.61	11q22.3	11-107.739925	6836	71552	**2.43**	**0.14**	**>>***	=	74	-0.1
286	ATM-63	11q22.3	11-107.741279	12948	106265	**2.31**	0.1	**>>***	=	71	0.0
436	Reference*	01q23.3	01-159.406366	3727	39550	0.98	0.05	=	=	51	-0.1
319	Reference*	03q13.2	03-113.667629	5675	49934	1	0.04	=	=	56	0.1
131	Reference*	03q21.3	03-129.999519	9287	64978	1.04	0.04	=	=	45	0.0
256	Reference*	04q25	04-110.941155	7326	62893	0.98	0.04	=	=	52	0.0
365	Reference*	05q33.3	05-158.137017	4864	45906	1	0.04	=	=	64	0.0
301	Reference*	06q22.33	06-129.511860	6238	51989	1.02	0.04	=	=	55	0.0
136	Reference*	07p14.3	07-030.974908	6312	44169	1	0.03	=	=	42	0.0
401	Reference*	08q12.2	08-061.913286	4485	46127	1.01	0.03	=	=	64	0.0
184	Reference*	09p24.2	09-002.644106	6696	47976	0.97	0.03	=	=	62	0.0
454	Reference*	13q13.1	13-031.879392	2918	32381	1.06	0.09	=	=	62	-0.1
485	Reference*	18q21.1	18-045.630186	2919	33560	0.99	0.04	=	=	47	-0.1
**Median value all probe values:**				**6354**	**51989**	**1.02**	**0.04**			**55**	**0**

Figure (v) MLPA results showing BRCA1:c.(?_-21)_(80+21_81-21)[3]

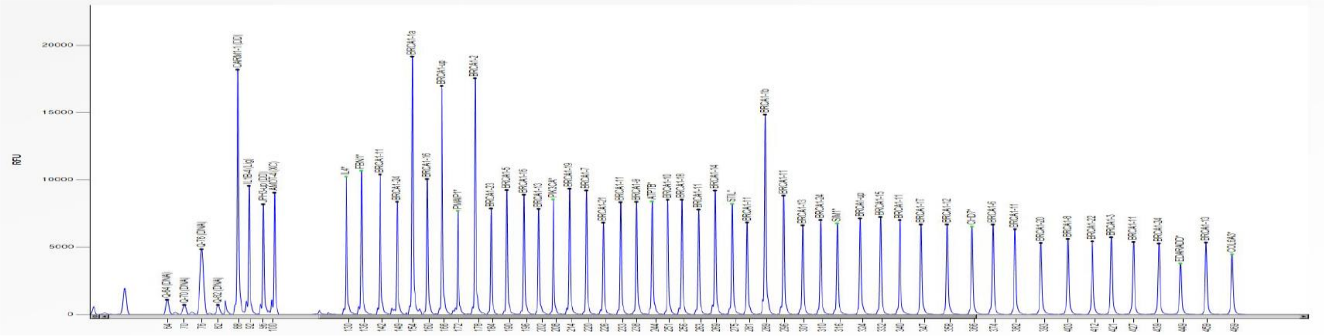

D [nt]	Gene-Exon	Chr.band	hg18 loc.	Height	Area	Ratio^{H}	Stdev	[REF]	[Sam]	Width	d[nt]
310	BRCA1-24	17q21.31	17-038.450829	7020	57899	1	0.04	=	=	68	-0.1
149	BRCA1-24	17q21.31	17-038.451166	8369	**61602**	0.98	0.05	=	=	42	-0.1
439	BRCA1-24	17q21.31	17-038.451283	5271	54489	0.91	0.04	=	=	95	0.0
184	BRCA1-23	17q21.31	17-038.453196	7868	54618	1.02	0.06	=	=	42	0.1
412	BRCA1-22	17q21.31	17-038.454659	5435	53145	0.91	0.05	=	=	70	0.0
226	BRCA1-21	17q21.31	17-038.456598	6823	51754	1.02	0.05	=	=	56	0.0
393	BRCA1-20	17q21.31	17-038.462606	5314	53162	0.95	0.04	=	=	73	0.0
214	BRCA1-19	17q21.31	17-038.468858	9346	70400	0.99	0.04	=	=	57	0.0
256	BRCA1-18	17q21.31	17-038.469436	8531	67019	0.98	0.04	=	=	52	0.0
347	BRCA1-17	17q21.31	17-038.473167	6687	58439	0.96	0.04	=	=	72	0.0
196	BRCA1-16	17q21.31	17-038.476425	8900	64626	1.02	0.07	=	=	58	0.0
160	BRCA1-16	17q21.31	17-038.476716	10057	70589	0.99	0.05	=	=	42	0.0
332	BRCA1-15	17q21.31	17-038.479958	7241	61009	0.99	0.04	=	=	56	0.0
269	BRCA1-14	17q21.31	17-038.482059	9209	75533	1	0.04	=	=	65	0.0
459	BRCA1-13	17q21.31	17-038.487745	5340	52303	0.92	0.04	=	=	64	0.0
202	BRCA1-13	17q21.31	17-038.487951	7836	57553	1.04	0.05	=	=	59	0.0
301	BRCA1-13	17q21.31	17-038.488025	6626	55518	0.98	0.04	=	=	63	0.0
358	BRCA1-12	17q21.31	17-038.496486	6688	60986	0.96	0.04	=	=	79	0.0
142	BRCA1-11	17q21.31	17-038.497036	10401	77951	1.04	0.05	=	=	54	-0.1
281	BRCA1-11	17q21.31	17-038.497460	6839	54426	0.98	0.04	=	=	51	0.0
427	BRCA1-11	17q21.31	17-038.498001	5383	53769	0.93	0.04	=	=	76	0.0
340	BRCA1-11	17q21.31	17-038.498514	7017	60421	0.99	0.05	=	=	75	-0.1
233	BRCA1-11	17q21.31	17-038.499023	8339	62637	0.99	0.04	=	=	51	0.1
296	BRCA1-11	17q21.31	17-038.499436	8835	73873	1	0.05	=	=	62	0.0
382	BRCA1-11	17q21.31	17-038.499815	6333	58857	0.98	0.04	=	=	64	0.0
263	BRCA1-11	17q21.31	17-038.500273	7789	59813	1.01	0.04	=	=	56	0.1
251	BRCA1-10	17q21.31	17-038.501402	8524	62687	1.01	0.05	=	=	46	0.0
238	BRCA1-9	17q21.31	17-038.502751	8365	63469	1	0.04	=	=	57	0.0
403	BRCA1-8	17q21.31	17-038.505340	5606	55119	0.95	0.04	=	=	71	0.1
220	BRCA1-7	17q21.31	17-038.509662	9216	66273	1	0.04	=	=	55	0.0
374	BRCA1-6	17q21.31	17-038.510430	6685	61339	0.94	0.05	=	=	70	0.0
190	BRCA1-5	17q21.31	17-038.512011	9245	66623	0.98	0.04	=	=	55	0.0
421	BRCA1-3	17q21.31	17-038.521268	5730	56171	0.93	0.05	=	=	67	0.1
178	BRCA1-2	17q21.31	17-038.529585	17548	128206	**1.85**	0.09	>>*	?	65	0.0
289	BRCA1-1b	17q21.31	17-038.530571	14851	123600	**1.86**	0.07	>>*	?	70	0.0
154	BRCA1-1a	17q21.31	17-038.530812	19160	140723	**1.89**	**0.13**	>>*	?	40	0.0
166	BRCA1-up	17q21.31	17-038.531706	16984	124335	**1.89**	**0.14**	>>*	?	58	0.0
324	BRCA1-up	17q21.31	17-038.535583	7138	61300	0.96	0.04	=	=	73	0.0
275	STIL*	01p33	01-047.538303	8214	65879	1.01	0.04	=	=	61	0.0
449	EDARADD*	01q42.3	01-234.657310	3779	40080	0.92	0.06	=	=	78	0.1
469	COL6A3*	02q37.3	02-237.961415	4467	47495	0.88	0.04	<<	=	71	0.0
208	PIK3CA*	03q26.32	03-180.399606	8550	60845	1.03	0.04	=	=	54	0.0
130	IL4*	05q31.1	05-132.037607	10230	74670	1.04	0.05	=	=	63	-0.1
316	SIM1*	06q16.3	06-101.001940	6772	56500	1	0.04	=	=	60	0.1
366	CHD7*	08q12.2	08-061.927614	6526	60352	0.94	0.05	=	=	74	0.0
244	ATP7B*	13q14.3	13-051.416301	8402	67482	1.02	0.04	=	=	62	0.1
136	FBN1*	15q21.1	15-046.501165	10684	81511	1.02	0.05	=	=	60	-0.1
172	PMAIP1*	18q21.32	18-055.720855	7692	59151	1	0.06	=	=	57	0.0
**Median value all probe values:**				**7812**	**60998**	**0.99**	**0.04**			**61.5**	**0.01**

Figure (vi) : MLPA results showing BRCA1:c.(?_-21)_(80+21_81-21)del

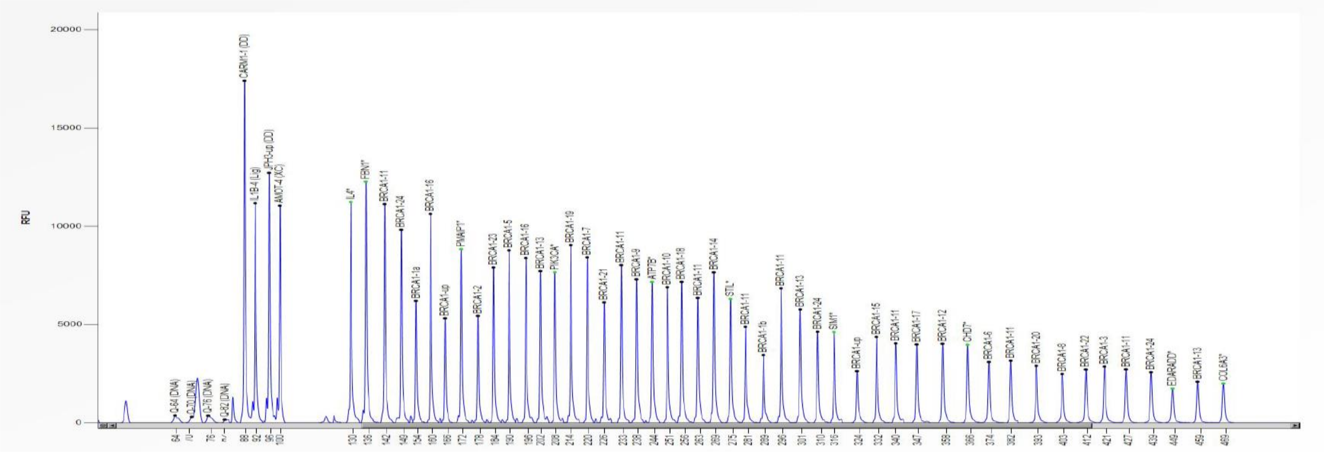

D [nt]	Gene-Exon	Chr.band	hg18 loc.	Height	Area	Ratio	Stdev	[REF]	[Sam]	Width	d[nt]
310	BRCA1-24	17q21.31	17-038.450829	4633	48952	0.96	0.03	=	=	74	0.0
149	BRCA1-24	17q21.31	17-038.451166	9823	85289	0.97	0.04	=	=	46	0.0
439	BRCA1-24	17q21.31	17-038.451283	2569	32989	0.99	0.07	=	=	84	0.1
184	BRCA1-23	17q21.31	17-038.453196	7903	68031	0.97	0.04	=	=	49	0.1
412	BRCA1-22	17q21.31	17-038.454659	2708	34790	0.95	0.04	=	=	103	0.0
226	BRCA1-21	17q21.31	17-038.456598	6125	57470	0.95	0.04	=	=	42	0.0
393	BRCA1-20	17q21.31	17-038.462606	2898	35829	0.91	0.04	=	=	85	0.0
214	BRCA1-19	17q21.31	17-038.468858	9043	84675	1.02	0.04	=	=	58	0.0
256	BRCA1-18	17q21.31	17-038.469436	7172	73357	1.07	0.05	=	=	72	0.0
347	BRCA1-17	17q21.31	17-038.473167	3985	43524	0.99	0.04	=	=	66	0.0
196	BRCA1-16	17q21.31	17-038.476425	8387	76111	0.98	0.03	=	=	67	0.0
160	BRCA1-16	17q21.31	17-038.476716	10633	91941	0.98	0.04	=	=	55	0.1
332	BRCA1-15	17q21.31	17-038.479958	4370	47826	0.92	0.04	=	=	63	0.0
269	BRCA1-14	17q21.31	17-038.482059	7656	77497	1.04	0.04	=	=	70	0.0
459	BRCA1-13	17q21.31	17-038.487745	2086	26796	0.91	0.04	=	=	78	0.0
202	BRCA1-13	17q21.31	17-038.487951	7722	69761	1.01	0.04	=	=	42	0.0
301	BRCA1-13	17q21.31	17-038.488025	5769	62206	1.16	0.05	=	=	71	-0.1
358	BRCA1-12	17q21.31	17-038.496486	4020	46642	1.01	0.04	=	=	79	0.0
142	BRCA1-11	17q21.31	17-038.497036	11133	99553	0.93	0.04	=	=	62	0.0
281	BRCA1-11	17q21.31	17-038.497460	4885	50953	0.92	0.03	=	=	63	0.0
427	BRCA1-11	17q21.31	17-038.498001	2715	32969	1.05	0.05	=	=	76	0.1
340	BRCA1-11	17q21.31	17-038.498514	4041	43858	0.96	0.04	=	=	45	0.0
233	BRCA1-11	17q21.31	17-038.499023	8027	74344	1.09	0.04	=	=	57	0.0
296	BRCA1-11	17q21.31	17-038.499436	6845	70307	1.09	0.04	>>	=	78	0.0
382	BRCA1-11	17q21.31	17-038.499815	3160	35513	0.92	0.03	=	=	45	0.0
263	BRCA1-11	17q21.31	17-038.500273	6356	64622	0.99	0.04	=	=	59	0.0
251	BRCA1-10	17q21.31	17-038.501402	6899	66767	0.98	0.03	=	=	65	0.0
238	BRCA1-9	17q21.31	17-038.502751	7301	71330	1.01	0.04	=	=	61	0.0
403	BRCA1-8	17q21.31	17-038.505340	2482	31113	0.86	0.03	<<	=	80	0.1
220	BRCA1-7	17q21.31	17-038.509662	8419	74929	0.99	0.04	=	=	51	0.0
374	BRCA1-6	17q21.31	17-038.510430	3094	37404	0.9	0.05	=	=	77	0.0
190	BRCA1-5	17q21.31	17-038.512011	8776	75774	0.91	0.04	=	=	44	0.0
421	BRCA1-3	17q21.31	17-038.521268	2860	36247	0.97	0.04	=	=	69	0.0
178	BRCA1-2	17q21.31	17-038.529585	5438	47716	**0.53**	0.02	<<*	=	50	0.0
289	BRCA1-1b	17q21.31	17-038.530571	3445	36080	**0.57**	0.02	<<*	=	54	0.0
154	BRCA1-1a	17q21.31	17-038.530812	6203	53655	**0.52**	0.02	<<*	=	39	0.0
166	BRCA1-up	17q21.31	17-038.531706	5314	45426	**0.53**	0.04	<<*	=	44	0.0
324	BRCA1-up	17q21.31	17-038.535583	2625	30501	**0.53**	0.02	<<*	=	71	0.0
275	STIL*	01p33	01-047.538303	6300	64352	1	0.04	=	=	77	0.0
449	EDARADD*	01q42.3	01-234.657310	1739	23727	1.04	0.04	=	=	91	0.0
469	COL6A3*	02q37.3	02-237.961415	1996	26927	1.03	0.04	=	=	68	0.0
208	PIK3CA*	03q26.32	03-180.399606	7660	66512	0.97	0.04	=	=	42	0.0
130	IL4*	05q31.1	05-132.037607	11241	102131	0.93	0.05	=	=	68	-0.1
316	SIM1*	06q16.3	06-101.001940	4615	47147	0.97	0.04	=	=	42	0.0
366	CHD7*	08q12.2	08-061.927614	3968	48068	1.06	0.04	=	=	90	0.0
244	ATP7B*	13q14.3	13-051.416301	7171	69794	1	0.03	=	=	57	0.0
136	FBN1*	15q21.1	15-046.501165	12277	115995	0.98	0.04	=	=	68	0.0
172	PMAIP1*	18q21.32	18-055.720855	8839	79057	1.04	0.04	=	=	63	0.0
**Median value all probe values:**				**5947**	**55562**	**0.97**	**0.04**			**64**	**0**

Figure (vii) : MLPA results showing BRCA1:c.(4185+21_4186-21)_(4357+21_4358-21)dup

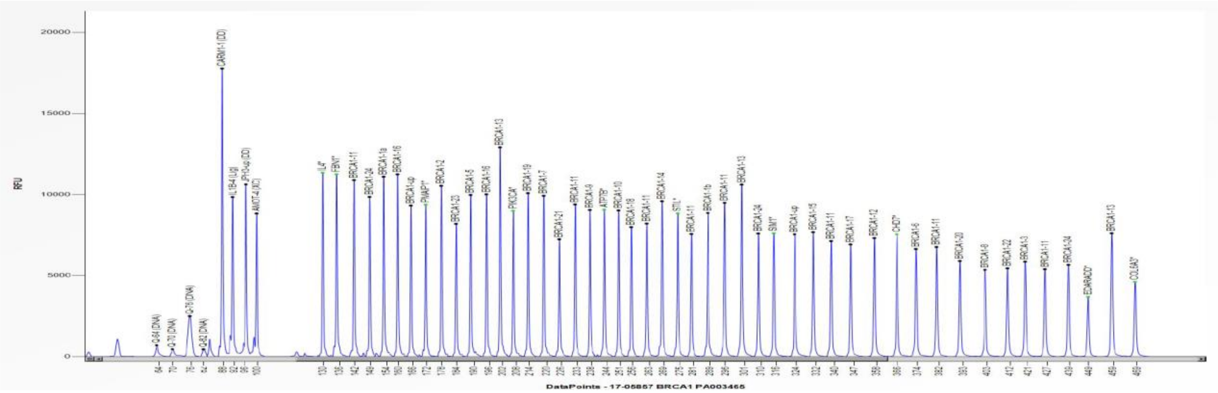

D [nt]	Gene-Exon	Chr.band	hg18 loc.	Height	Area	Ratio [H]	Stdev	[REF]	[Sam]	Width	d[nt]
310	BRCA1-24	17q21.31	17-038.450829	7598	62869	*0.97*	0.06	=	=	55	-0.1
149	BRCA1-24	17q21.31	17-038.451166	9852	68436	*1.07*	0.09	=	=	39	0.3
439	BRCA1-24	17q21.31	17-038.451283	5661	56057	*0.89*	0.05	=	=	80	0.3
184	BRCA1-23	17q21.31	17-038.453196	8192	56869	*0.96*	0.06	=	=	35	0.2
412	BRCA1-22	17q21.31	17-038.454659	5450	53417	*0.87*	0.07	=	=	71	0.1
226	BRCA1-21	17q21.31	17-038.456598	7242	54904	*0.93*	0.06	=	=	49	0.0
393	BRCA1-20	17q21.31	17-038.462606	5902	57809	*0.87*	0.05	=	=	77	0.2
214	BRCA1-19	17q21.31	17-038.468858	10088	73582	*0.99*	0.06	=	=	44	0.1
256	BRCA1-18	17q21.31	17-038.469436	7989	60090	*0.84*	0.05	<<	=	43	-0.1
347	BRCA1-17	17q21.31	17-038.473167	6925	60322	*0.91*	0.06	=	=	75	0.0
196	BRCA1-16	17q21.31	17-038.476425	10015	70446	*1.11*	0.08	=	=	56	0.1
160	BRCA1-16	17q21.31	17-038.476716	11247	77949	*1.08*	0.08	=	=	36	0.3
332	BRCA1-15	17q21.31	17-038.479958	7681	64766	*0.96*	0.06	=	=	65	-0.1
269	BRCA1-14	17q21.31	17-038.482059	9590	77853	*0.97*	0.06	=	=	63	-0.1
459	BRCA1-13	17q21.31	17-038.487745	7614	78809	*1.24*	0.07	>>	=	78	0.3
202	BRCA1-13	17q21.31	17-038.487951	12913	93591	***1.59***	**0.11**	>>*	=	56	0.0
301	BRCA1-13	17q21.31	17-038.488025	10617	90838	***1.31***	0.08	>>*	=	68	0.0
358	BRCA1-12	17q21.31	17-038.496486	7316	64876	*0.97*	0.06	=	=	74	0.0
142	BRCA1-11	17q21.31	17-038.497036	10892	78147	*1.05*	0.07	=	=	48	0.2
281	BRCA1-11	17q21.31	17-038.497460	7568	58574	*0.99*	0.06	=	=	48	0.0
427	BRCA1-11	17q21.31	17-038.498001	5396	54332	*0.84*	0.06	=	=	71	0.1
340	BRCA1-11	17q21.31	17-038.498514	7131	62675	*0.93*	0.06	=	=	77	-0.2
233	BRCA1-11	17q21.31	17-038.499023	9390	69937	*1.11*	0.08	=	=	52	0.1
296	BRCA1-11	17q21.31	17-038.499436	9487	77879	*1.02*	0.07	=	=	53	-0.2
382	BRCA1-11	17q21.31	17-038.499815	6764	61237	*0.98*	0.06	=	=	72	0.2
263	BRCA1-11	17q21.31	17-038.500273	8209	63418	*0.97*	0.06	=	=	57	-0.1
251	BRCA1-10	17q21.31	17-038.501402	9020	68344	*1.02*	0.07	=	=	45	-0.1
238	BRCA1-9	17q21.31	17-038.502751	9051	65377	*1.02*	0.07	=	=	44	-0.1
403	BRCA1-8	17q21.31	17-038.505340	5355	51286	*0.81*	0.05	<<	=	70	0.1
220	BRCA1-7	17q21.31	17-038.509662	9924	73629	*1.01*	0.06	=	=	66	0.0
374	BRCA1-6	17q21.31	17-038.510430	6640	61287	*0.84*	0.07	=	=	65	0.2
190	BRCA1-5	17q21.31	17-038.512011	9975	72626	*1*	0.07	=	=	63	0.1
421	BRCA1-3	17q21.31	17-038.521268	5859	58568	*0.87*	0.06	=	=	72	0.1
178	BRCA1-2	17q21.31	17-038.529585	10542	72389	*1.05*	0.07	=	=	35	0.3
289	BRCA1-1b	17q21.31	17-038.530571	8864	73877	*1.01*	0.07	=	=	71	-0.2
154	BRCA1-1a	17q21.31	17-038.530812	11099	82300	*1*	0.08	=	=	58	0.3
166	BRCA1-up	17q21.31	17-038.531706	9323	64975	*0.96*	0.06	=	=	47	0.2
324	BRCA1-up	17q21.31	17-038.535583	7551	64451	*0.92*	0.06	=	=	60	0.1
275	STIL*	01p33	01-047.538303	8837	70066	*0.97*	0.06	=	=	59	-0.1
449	EDARADD*	01q42.3	01-234.657310	3690	36999	*0.87*	0.08	=	=	68	0.2
469	COL6A3*	02q37.3	02-237.961415	4611	50029	*0.84*	0.06	=	=	72	0.4
208	PIK3CA*	03q26.32	03-180.399606	9000	65617	*1.04*	0.07	=	=	55	0.1
130	IL4*	05q31.1	05-132.037607	11345	74714	*1.06*	**0.11**	=	=	49	0.3
316	SIM1*	06q16.3	06-101.001940	7611	63057	*1*	0.06	=	=	67	-0.2
366	CHD7*	08q12.2	08-061.927614	7550	67522	*0.99*	0.06	=	=	58	0.4
244	ATP7B*	13q14.3	13-051.416301	9067	71671	*1.02*	0.06	=	=	60	0.0
136	FBN1*	15q21.1	15-046.501165	11252	83430	*1.04*	0.08	=	=	60	0.3
172	PMAIP1*	18q21.32	18-055.720855	9377	65887	*1.13*	0.07	=	=	42	0.2
**Median value all probe values:**				**8523**	**65497**	**0.99**	**0.06**			**59.5**	**0.09**

Figure (viii) MLPA results showing BRCA1: c.(4357+21_4358-21)_(4484+21_4485-21)del

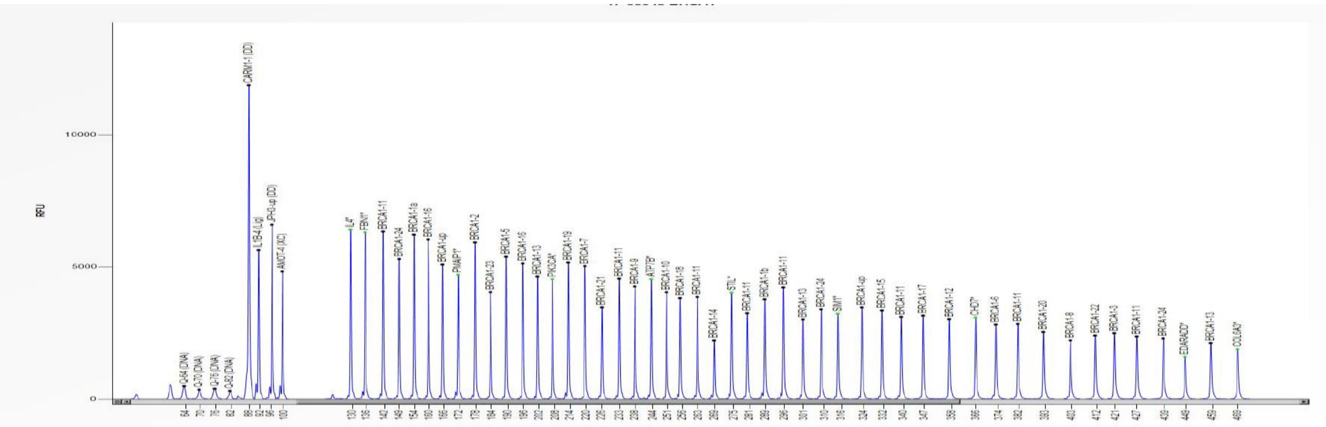

D [nt]	Gene-Exon	Chr.band	hg18 loc.	Height	Area	Ratio[H]	Stdev	[REF]	[Sam]	Width	d[nt]
310	BRCA1-24	17q21.31	17-038.450829	3400	25440	*1*	0.06	=	=	62	0.0
149	BRCA1-24	17q21.31	17-038.451166	5305	32972	*0.96*	0.05	=	=	46	-0.1
439	BRCA1-24	17q21.31	17-038.451283	2299	20722	*1.08*	0.05	=	=	64	0.0
184	BRCA1-23	17q21.31	17-038.453196	4052	25671	*1.06*	0.05	=	=	54	0.0
412	BRCA1-22	17q21.31	17-038.454659	2406	20868	*1*	0.05	=	=	60	0.0
226	BRCA1-21	17q21.31	17-038.456598	3474	23114	*1.04*	0.05	=	=	56	0.0
393	BRCA1-20	17q21.31	17-038.462606	2542	21789	*0.99*	0.05	=	=	77	0.0
214	BRCA1-19	17q21.31	17-038.468858	5176	34114	*1.02*	0.04	=	=	66	0.1
256	BRCA1-18	17q21.31	17-038.469436	3827	25891	*1.1*	0.06	=	=	52	0.0
347	BRCA1-17	17q21.31	17-038.473167	3161	24784	*1.03*	0.05	=	=	52	0.0
196	BRCA1-16	17q21.31	17-038.476425	5139	32363	*1.03*	0.04	=	=	61	0.1
160	BRCA1-16	17q21.31	17-038.476716	6047	37024	*1*	0.04	=	=	52	0.0
332	BRCA1-15	17q21.31	17-038.479958	3357	25841	*1.05*	0.05	=	=	64	0.0
269	BRCA1-14	17q21.31	17-038.482059	2220	16274	***0.51***	0.02	<<*	?	59	0.1
459	BRCA1-13	17q21.31	17-038.487745	2122	19348	*1.02*	0.04	=	=	53	0.0
202	BRCA1-13	17q21.31	17-038.487951	4643	29782	*1.01*	0.05	=	=	45	0.1
301	BRCA1-13	17q21.31	17-038.488025	3015	22176	*0.99*	0.05	=	=	69	0.1
358	BRCA1-12	17q21.31	17-038.496486	3026	23566	*1*	0.04	=	=	58	0.0
142	BRCA1-11	17q21.31	17-038.497036	6349	40359	*1*	0.05	=	=	67	-0.1
281	BRCA1-11	17q21.31	17-038.497460	3255	23370	*0.96*	0.05	=	=	59	0.0
427	BRCA1-11	17q21.31	17-038.498001	2370	21317	*1.03*	0.05	=	=	60	0.0
340	BRCA1-11	17q21.31	17-038.498514	3112	24041	*0.99*	0.04	=	=	57	0.1
233	BRCA1-11	17q21.31	17-038.499023	4564	30155	*1.03*	0.05	=	=	56	0.0
296	BRCA1-11	17q21.31	17-038.499436	4230	31370	*1.04*	0.04	=	=	69	0.0
382	BRCA1-11	17q21.31	17-038.499815	2848	24132	*0.99*	0.05	=	=	72	0.0
263	BRCA1-11	17q21.31	17-038.500273	3871	26707	*1.03*	0.05	=	=	57	0.1
251	BRCA1-10	17q21.31	17-038.501402	4048	27431	*0.97*	0.04	=	=	45	0.0
238	BRCA1-9	17q21.31	17-038.502751	4269	28759	*1*	0.05	=	=	53	0.1
403	BRCA1-8	17q21.31	17-038.505340	2224	19510	*0.95*	0.05	=	=	68	-0.1
220	BRCA1-7	17q21.31	17-038.509662	5039	32242	*1.02*	0.05	=	=	45	0.0
374	BRCA1-6	17q21.31	17-038.510430	2823	23222	*0.96*	0.05	=	=	60	0.0
190	BRCA1-5	17q21.31	17-038.512011	5396	34007	*1*	0.04	=	=	52	0.0
421	BRCA1-3	17q21.31	17-038.521268	2497	22144	*1.01*	0.06	=	=	69	0.0
178	BRCA1-2	17q21.31	17-038.529585	5935	36842	*1.06*	0.05	=	=	63	0.0
289	BRCA1-1b	17q21.31	17-038.530571	3779	28299	*1*	0.06	=	=	61	0.1
154	BRCA1-1a	17q21.31	17-038.530812	6228	38954	*1.01*	0.07	=	=	55	0.0
166	BRCA1-up	17q21.31	17-038.531706	5098	31145	*1.12*	0.06	=	=	54	0.0
324	BRCA1-up	17q21.31	17-038.535583	3472	25928	*1.03*	0.06	=	=	51	0.0
275	STIL*	01p33	01-047.538303	4030	28997	*1*	0.05	=	=	56	0.0
449	EDARADD*	01q42.3	01-234.657310	1598	14542	*1.05*	0.05	=	=	60	0.0
469	COL6A3*	02q37.3	02-237.961415	1888	17946	*1.03*	0.04	=	=	67	0.0
208	PIK3CA*	03q26.32	03-180.399606	4543	28786	*0.98*	0.05	=	=	59	0.0
130	IL4*	05q31.1	05-132.037607	6419	38705	*0.99*	0.05	=	=	50	0.0
316	SIM1*	06q16.3	06-101.001940	3234	23962	*0.98*	0.04	=	=	65	0.1
366	CHD7*	08q12.2	08-061.927614	3083	25244	*1.05*	0.06	=	=	70	0.0
244	ATP7B*	13q14.3	13-051.416301	4541	29941	*1.1*	0.05	=	=	45	0.0
136	FBN1*	15q21.1	15-046.501165	6318	40168	*0.98*	0.04	=	=	64	-0.1
172	PMAIP1*	18q21.32	18-055.720855	4713	30321	*0.97*	0.04	=	=	59	0.0
**Median value all probe values:**				**3803**	**25910**	**1.01**	**0.05**			**59**	**0.01**

Figure (ix) : MLPA results showing BRCA1:c.(4986+21_4987-21)_(5074+21_5075-21)del

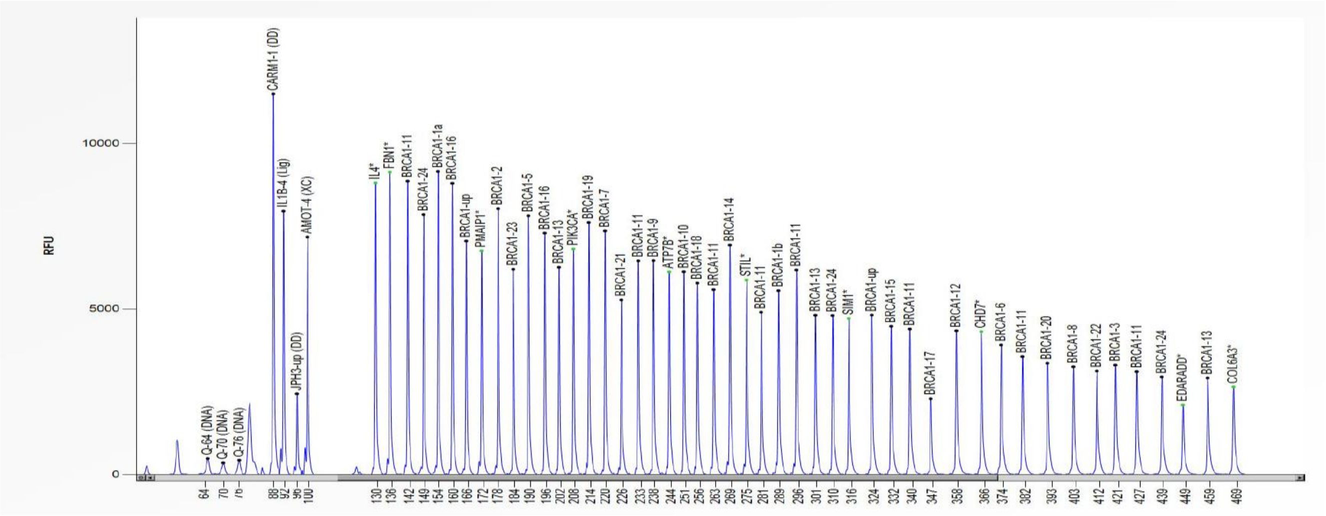

D [nt]	Gene-Exon	Chr.band	hg18 loc.	Height	Area	Ratio ^{H}	Stdev	[REF]	[Sam]	Width	d[nt]
310	BRCA1-24	17q21.31	17-038.450829	4806	45507	*0.96*	0.03	=	=	71	0.0
149	BRCA1-24	17q21.31	17-038.451166	7857	59695	*0.97*	0.03	=	=	63	0.1
439	BRCA1-24	17q21.31	17-038.451283	2950	34186	*0.94*	0.05	=	=	97	0.1
184	BRCA1-23	17q21.31	17-038.453196	6204	46759	*0.98*	**0.12**	=	=	54	0.0
412	BRCA1-22	17q21.31	17-038.454659	3130	34778	*0.93*	0.03	=	=	63	0.1
226	BRCA1-21	17q21.31	17-038.456598	5278	42893	*0.93*	0.04	=	=	64	-0.1
393	BRCA1-20	17q21.31	17-038.462606	3366	36924	*0.94*	0.03	=	=	77	0.1
214	BRCA1-19	17q21.31	17-038.468858	7618	62450	*0.97*	0.03	=	=	66	0.0
256	BRCA1-18	17q21.31	17-038.469436	5788	48701	*1.03*	**0.12**	=	=	57	0.0
347	BRCA1-17	17q21.31	17-038.473167	2292	22815	***0.54***	0.02	<<*	=	55	0.0
196	BRCA1-16	17q21.31	17-038.476425	7298	56931	*1.02*	0.04	=	=	60	0.0
160	BRCA1-16	17q21.31	17-038.476716	8801	65086	*0.98*	0.03	=	=	52	0.0
332	BRCA1-15	17q21.31	17-038.479958	4481	44060	*0.95*	0.03	=	=	70	-0.1
269	BRCA1-14	17q21.31	17-038.482059	6935	62710	*0.98*	0.05	=	=	68	0.0
459	BRCA1-13	17q21.31	17-038.487745	2919	33721	*0.96*	0.05	=	=	69	0.1
202	BRCA1-13	17q21.31	17-038.487951	6265	48781	*0.97*	0.03	=	=	50	-0.1
301	BRCA1-13	17q21.31	17-038.488025	4814	45182	*0.96*	0.03	=	=	67	-0.1
358	BRCA1-12	17q21.31	17-038.496486	4344	45375	*1.01*	0.03	=	=	87	-0.1
142	BRCA1-11	17q21.31	17-038.497036	8868	67903	*0.93*	0.03	=	=	58	0.0
281	BRCA1-11	17q21.31	17-038.497460	4907	44186	*0.98*	0.03	=	=	54	0.0
427	BRCA1-11	17q21.31	17-038.498001	3113	35296	*1.01*	0.03	=	=	81	0.1
340	BRCA1-11	17q21.31	17-038.498514	4399	44247	*1.01*	0.03	=	=	72	-0.1
233	BRCA1-11	17q21.31	17-038.499023	6458	53935	*0.99*	0.03	=	=	60	0.0
296	BRCA1-11	17q21.31	17-038.499436	6182	58737	*1.03*	0.04	=	=	82	0.0
382	BRCA1-11	17q21.31	17-038.499815	3566	38520	*0.96*	0.04	=	=	69	0.1
263	BRCA1-11	17q21.31	17-038.500273	5590	49332	*0.96*	0.03	=	=	59	0.0
251	BRCA1-10	17q21.31	17-038.501402	6130	50589	*1*	0.03	=	=	52	0.0
238	BRCA1-9	17q21.31	17-038.502751	6467	54076	*0.98*	0.03	=	=	55	-0.1
403	BRCA1-8	17q21.31	17-038.505340	3258	35439	*0.96*	0.06	=	=	89	0.0
220	BRCA1-7	17q21.31	17-038.509662	7365	58739	*0.98*	0.03	=	=	55	0.0
374	BRCA1-6	17q21.31	17-038.510430	3917	41962	*0.96*	0.04	=	=	69	0.0
190	BRCA1-5	17q21.31	17-038.512011	7821	60819	*0.98*	0.03	=	=	59	0.0
421	BRCA1-3	17q21.31	17-038.521268	3309	36730	*0.95*	0.04	=	=	63	0.0
178	BRCA1-2	17q21.31	17-038.529585	8036	63559	*0.96*	0.03	=	=	62	0.0
289	BRCA1-1b	17q21.31	17-038.530571	5558	52147	*0.96*	0.03	=	=	73	0.0
154	BRCA1-1a	17q21.31	17-038.530812	9161	70344	*0.95*	0.04	=	=	55	0.1
166	BRCA1-up	17q21.31	17-038.531706	7059	53617	*0.97*	**0.13**	=	=	56	0.0
324	BRCA1-up	17q21.31	17-038.535583	4820	46657	*0.92*	0.03	=	=	83	-0.1
275	STIL*	01p33	01-047.538303	5878	52894	*1*	0.03	=	=	65	0.0
449	EDARADD*	01q42.3	01-234.657310	2103	25174	*1.03*	0.03	=	=	76	0.1
469	COL6A3*	02q37.3	02-237.961415	2651	32025	*1.04*	0.05	=	=	73	0.0
208	PIK3CA*	03q26.32	03-180.399606	6817	52617	*1*	0.03	=	=	63	0.0
130	IL4*	05q31.1	05-132.037607	8815	66773	*0.97*	0.03	=	=	55	-0.1
316	SIM1*	06q16.3	06-101.001940	4718	43618	*1.01*	0.04	=	=	64	-0.1
366	CHD7*	08q12.2	08-061.927614	4319	45302	*1.04*	0.04	=	=	69	0.0
244	ATP7B*	13q14.3	13-051.416301	6126	52547	*0.97*	0.04	=	=	55	-0.1
136	FBN1*	15q21.1	15-046.501165	9140	70585	*0.99*	0.03	=	=	58	0.0
172	PMAIP1*	18q21.32	18-055.720855	6761	53635	*0.99*	0.03	=	=	63	0.0
**Median value all probe values:**				**5689**	**48741**	**0.97**	**0.03**			**63**	**-0.01**

Figure (x) : MLPA results showing BRCA1:c.(5277+21_5278-21)_(5592+21_*21_?)del

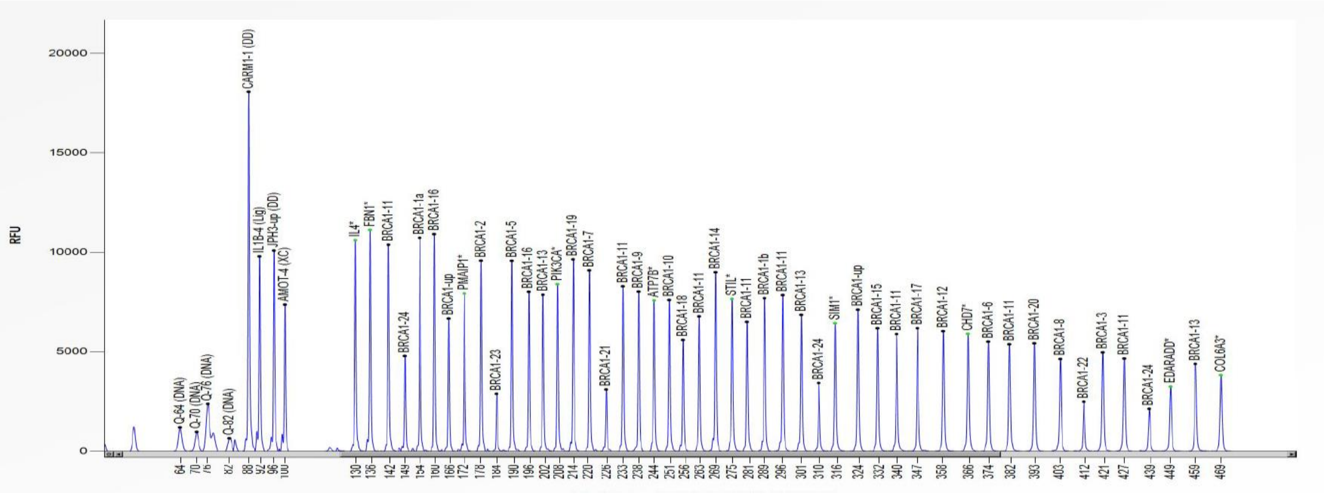

D [nt]	Gene-Exon	Chr.band	hg18 loc.	Height	Area	Ratio H	Stdev	[REF]	[Sam]	Width	d[nt]
310	BRCA1-24	17q21.31	17-038.450829	3434	30063	***0.52***	0.02	<<*	=	57	0.0
149	BRCA1-24	17q21.31	17-038.451166	4792	34896	***0.53***	0.02	<<*	=	36	0.1
439	BRCA1-24	17q21.31	17-038.451283	2134	21956	***0.44***	0.02	<<*	=	52	0.0
184	BRCA1-23	17q21.31	17-038.453196	2888	20981	***0.46***	0.07	<<*	=	36	0.0
412	BRCA1-22	17q21.31	17-038.454659	2492	25150	***0.49***	0.04	<<*	=	48	0.0
226	BRCA1-21	17q21.31	17-038.456598	3106	24149	***0.49***	0.02	<<*	=	43	0.0
393	BRCA1-20	17q21.31	17-038.462606	5427	51630	*1.01*	0.05	=	=	91	0.0
214	BRCA1-19	17q21.31	17-038.468858	9647	73484	*1.02*	0.04	=	=	52	0.0
256	BRCA1-18	17q21.31	17-038.469436	5595	43875	*0.89*	**0.11**	=	=	54	0.0
347	BRCA1-17	17q21.31	17-038.473167	6185	54896	*1*	0.06	=	=	46	-0.1
196	BRCA1-16	17q21.31	17-038.476425	8018	58637	*0.96*	0.04	=	=	54	0.0
160	BRCA1-16	17q21.31	17-038.476716	10916	74475	*1.06*	0.03	=	=	42	0.0
332	BRCA1-15	17q21.31	17-038.479958	6184	53284	*0.96*	0.04	=	=	55	0.0
269	BRCA1-14	17q21.31	17-038.482059	8998	72820	*1.01*	0.05	=	=	55	0.0
459	BRCA1-13	17q21.31	17-038.487745	4392	47189	*0.91*	0.08	=	=	81	0.0
202	BRCA1-13	17q21.31	17-038.487951	7869	58669	*1*	0.04	=	=	58	0.0
301	BRCA1-13	17q21.31	17-038.488025	6857	58630	*1*	0.05	=	=	57	0.0
358	BRCA1-12	17q21.31	17-038.496486	6037	55595	*0.97*	0.05	=	=	72	0.0
142	BRCA1-11	17q21.31	17-038.497036	10383	76638	*1.02*	0.03	=	=	64	0.0
281	BRCA1-11	17q21.31	17-038.497460	6505	52729	*0.97*	0.03	=	=	56	0.1
427	BRCA1-11	17q21.31	17-038.498001	4662	47582	*0.95*	0.07	=	=	77	-0.1
340	BRCA1-11	17q21.31	17-038.498514	5892	54480	*0.95*	0.04	=	=	70	-0.1
233	BRCA1-11	17q21.31	17-038.499023	8292	62285	*1.01*	0.04	=	=	54	0.0
296	BRCA1-11	17q21.31	17-038.499436	7854	65785	*1*	0.04	=	=	69	0.0
382	BRCA1-11	17q21.31	17-038.499815	5380	52359	*0.94*	0.07	=	=	61	0.0
263	BRCA1-11	17q21.31	17-038.500273	6781	53393	*0.95*	0.03	=	=	50	0.0
251	BRCA1-10	17q21.31	17-038.501402	7605	58924	*0.97*	0.03	=	=	51	0.0
238	BRCA1-9	17q21.31	17-038.502751	8026	61417	*1.01*	0.03	=	=	55	0.0
403	BRCA1-8	17q21.31	17-038.505340	4639	46779	*0.89*	0.06	=	=	86	0.0
220	BRCA1-7	17q21.31	17-038.509662	9095	66746	*1.01*	0.05	=	=	46	0.1
374	BRCA1-6	17q21.31	17-038.510430	5511	52980	*0.89*	0.04	=	=	70	0.0
190	BRCA1-5	17q21.31	17-038.512011	9574	69368	*1.01*	0.04	=	=	54	0.0
421	BRCA1-3	17q21.31	17-038.521268	4970	50118	*0.91*	0.07	=	=	66	-0.1
178	BRCA1-2	17q21.31	17-038.529585	9581	69469	*1*	0.04	=	=	48	0.0
289	BRCA1-1b	17q21.31	17-038.530571	7696	64083	*1.01*	0.04	=	=	58	0.1
154	BRCA1-1a	17q21.31	17-038.530812	10728	75201	*1*	0.05	=	=	35	0.0
166	BRCA1-up	17q21.31	17-038.531706	6673	47361	*0.93*	**0.15**	=	=	51	0.0
324	BRCA1-up	17q21.31	17-038.535583	7118	61601	*1.04*	0.04	=	=	65	0.0
275	STIL*	01p33	01-047.538303	7660	64824	*1.02*	0.03	=	=	68	0.0
449	EDARADD*	01q42.3	01-234.657310	3253	34490	*0.95*	0.07	=	=	57	0.0
469	COL6A3*	02q37.3	02-237.961415	3830	41619	*0.89*	0.06	=	=	53	0.1
208	PIK3CA*	03q26.32	03-180.399606	8402	61432	*1*	0.03	=	=	54	0.0
130	IL4*	05q31.1	05-132.037607	10613	78133	*1.01*	0.05	=	=	72	0.0
316	SIM1*	06q16.3	06-101.001940	6438	55460	*1.03*	0.03	=	=	64	0.0
366	CHD7*	08q12.2	08-061.927614	5908	56477	*0.99*	0.04	=	=	81	0.0
244	ATP7B*	13q14.3	13-051.416301	7583	59593	*1.01*	0.04	=	=	50	0.0
136	FBN1*	15q21.1	15-046.501165	11131	80806	*1.01*	0.03	=	=	50	0.0
172	PMAIP1*	18q21.32	18-055.720855	7933	59606	*0.99*	0.04	=	=	63	-0.1
**Median value all probe values:**				6727	56036	0.99	**0.04**			55	0

Figure (xi): MLPA results showing BRCA1: c.(5332+21_5333-21)_(5406+21_5407-21)del

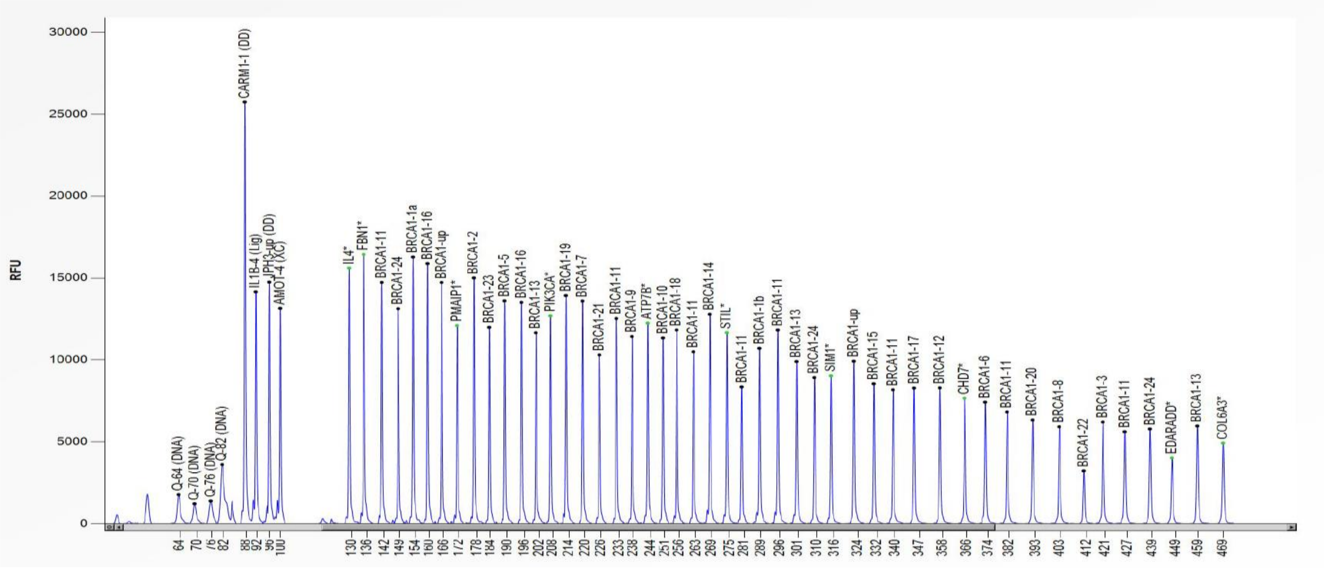

D [nt]	Gene-Exon	Chr.band	hg18 loc.	Height	Area	Ratio	Stdev	[REF]	[Sam]	Width	d[nt]
310	BRCA1-24	17q21.31	17-038.450829	8922	71566	*0.92*	0.04	=	=	62	-0.1
149	BRCA1-24	17q21.31	17-038.451166	13127	91875	*0.98*	0.05	=	=	44	0.0
439	BRCA1-24	17q21.31	17-038.451283	5786	57010	*0.85*	0.05	<<	=	85	0.1
184	BRCA1-23	17q21.31	17-038.453196	11991	81619	*0.97*	0.05	=	=	41	0.0
412	BRCA1-22	17q21.31	17-038.454659	3225	30881	***0.49***	0.03	<<*	=	66	0.1
226	BRCA1-21	17q21.31	17-038.456598	10307	75198	*0.99*	0.05	=	=	54	-0.1
393	BRCA1-20	17q21.31	17-038.462606	6331	59093	*0.9*	0.04	=	=	70	0.0
214	BRCA1-19	17q21.31	17-038.468858	13932	101925	*0.97*	0.05	=	=	60	0.0
256	BRCA1-18	17q21.31	17-038.469436	11828	88576	*0.94*	0.05	=	=	56	0.0
347	BRCA1-17	17q21.31	17-038.473167	8284	71181	*0.95*	0.05	=	=	84	-0.1
196	BRCA1-16	17q21.31	17-038.476425	13514	96573	*1.03*	0.05	=	=	65	0.0
160	BRCA1-16	17q21.31	17-038.476716	15883	109442	*1.02*	0.05	=	=	44	-0.1
332	BRCA1-15	17q21.31	17-038.479958	8545	71316	*0.88*	0.06	=	=	70	0.0
269	BRCA1-14	17q21.31	17-038.482059	12788	101634	*0.94*	0.05	=	=	70	0.0
459	BRCA1-13	17q21.31	17-038.487745	5971	59626	*0.9*	0.05	=	=	76	0.0
202	BRCA1-13	17q21.31	17-038.487951	11658	85089	*0.99*	0.05	=	=	60	0.0
301	BRCA1-13	17q21.31	17-038.488025	9903	81778	*0.94*	0.06	=	=	70	0.0
358	BRCA1-12	17q21.31	17-038.496486	8297	72106	*0.95*	0.05	=	=	101	0.0
142	BRCA1-11	17q21.31	17-038.497036	14732	105493	*0.97*	0.05	=	=	59	0.0
281	BRCA1-11	17q21.31	17-038.497460	8355	64930	*0.86*	0.04	=	=	56	-0.1
427	BRCA1-11	17q21.31	17-038.498001	5613	55255	*0.86*	0.04	<<	=	78	0.1
340	BRCA1-11	17q21.31	17-038.498514	8177	68686	*0.91*	0.05	=	=	80	0.0
233	BRCA1-11	17q21.31	17-038.499023	12533	90333	*1.01*	0.05	=	=	57	-0.1
296	BRCA1-11	17q21.31	17-038.499436	11826	95950	*0.98*	0.05	=	=	77	0.0
382	BRCA1-11	17q21.31	17-038.499815	6823	62266	*0.88*	0.05	=	=	89	0.0
263	BRCA1-11	17q21.31	17-038.500273	10498	79904	*0.94*	0.05	=	=	59	0.0
251	BRCA1-10	17q21.31	17-038.501402	11343	82621	*0.95*	0.04	=	=	48	0.0
238	BRCA1-9	17q21.31	17-038.502751	11433	84443	*0.94*	0.04	=	=	53	0.0
403	BRCA1-8	17q21.31	17-038.505340	5921	57776	*0.9*	0.05	=	=	88	0.0
220	BRCA1-7	17q21.31	17-038.509662	13595	97146	*0.97*	0.04	=	=	62	0.0
374	BRCA1-6	17q21.31	17-038.510430	7422	67034	*0.91*	0.05	=	=	85	0.0
190	BRCA1-5	17q21.31	17-038.512011	13607	96677	*0.97*	0.05	=	=	56	0.0
421	BRCA1-3	17q21.31	17-038.521268	6209	60510	*0.84*	0.06	=	=	83	0.0
178	BRCA1-2	17q21.31	17-038.529585	15010	106390	*1.02*	0.05	=	=	63	-0.1
289	BRCA1-1b	17q21.31	17-038.530571	10711	87604	*0.94*	0.05	=	=	73	0.0
154	BRCA1-1a	17q21.31	17-038.530812	16283	116518	*0.99*	0.05	=	=	60	0.0
166	BRCA1-up	17q21.31	17-038.531706	14729	103156	*1.01*	0.05	=	=	53	-0.1
324	BRCA1-up	17q21.31	17-038.535583	9924	83042	*0.99*	0.05	=	=	72	-0.1
275	STIL*	01p33	01-047.538303	11667	90198	*1.01*	0.06	=	=	68	0.0
449	EDARADD*	01q42.3	01-234.657310	4017	40160	*0.92*	0.07	=	=	81	0.0
469	COL6A3*	02q37.3	02-237.961415	4925	51725	*0.86*	0.06	=	=	75	0.1
208	PIK3CA*	03q26.32	03-180.399606	12696	89172	*1.03*	0.05	=	=	53	-0.1
130	IL4*	05q31.1	05-132.037607	15607	110898	*1.05*	0.05	=	=	65	0.1
316	SIM1*	06q16.3	06-101.001940	9032	71531	*0.99*	0.05	=	=	68	0.0
366	CHD7*	08q12.2	08-061.927614	7667	69313	*0.92*	0.04	=	=	80	0.1
244	ATP7B*	13q14.3	13-051.416301	12246	93579	*0.99*	0.05	=	=	65	0.0
136	FBN1*	15q21.1	15-046.501165	16444	119438	*1.06*	0.05	=	=	61	0.1
172	PMAIP1*	18q21.32	18-055.720855	12101	86175	*1.01*	0.05	=	=	46	0.0
**Median value all probe values:**				**11027**	**82832**	**0.95**	**0.05**			**65**	**-0.01**

Figure (xii): MLPA results showing BRCA1:c.(5406+21_5407-21)_(*21_?)del

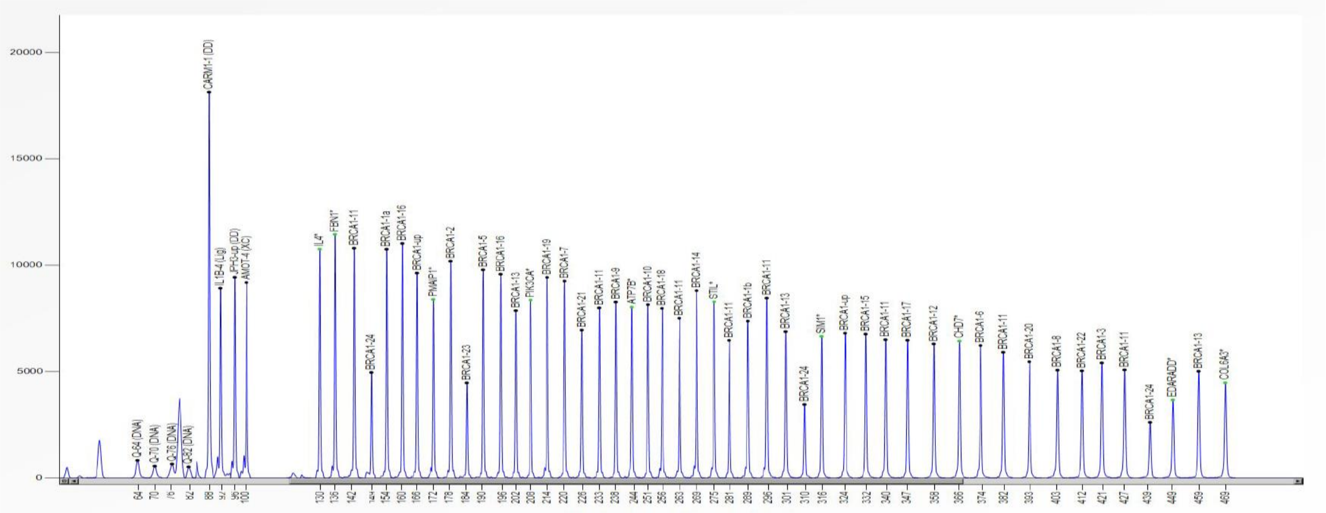

D [nt]	Gene-Exon	Chr.band	hg18 loc.	Height	Area	Ratio ^{H}	Stdev	[REF]	[Sam]	Width	d[nt]
310	BRCA1-24	17q21.31	17-038.450829	3455	26233	*0.49*	0.01	<<*	<<*	55	0.0
149	BRCA1-24	17q21.31	17-038.451166	4961	30739	*0.52*	0.02	<<*	<<*	21	0.1
439	BRCA1-24	17q21.31	17-038.451283	2616	25247	*0.48*	0.02	<<*	<<*	76	0.0
184	BRCA1-23	17q21.31	17-038.453196	4472	30262	*0.52*	0.01	<<*	<<*	59	0.1
412	BRCA1-22	17q21.31	17-038.454659	5039	46230	*0.93*	0.03	=	=	69	0.0
226	BRCA1-21	17q21.31	17-038.456598	6953	47941	*0.96*	0.03	=	=	62	-0.1
393	BRCA1-20	17q21.31	17-038.462606	5465	47701	*0.96*	0.02	=	=	67	0.1
214	BRCA1-19	17q21.31	17-038.468858	9430	63109	*0.93*	0.03	=	=	52	-0.1
256	BRCA1-18	17q21.31	17-038.469436	7973	56020	*0.91*	0.02	<<	=	54	0.0
347	BRCA1-17	17q21.31	17-038.473167	6476	50626	*0.96*	0.03	=	=	51	0.0
196	BRCA1-16	17q21.31	17-038.476425	9580	62327	*1.02*	0.03	=	=	59	0.0
160	BRCA1-16	17q21.31	17-038.476716	11026	69949	*0.98*	0.04	=	=	61	0.0
332	BRCA1-15	17q21.31	17-038.479958	6766	52901	*0.93*	0.03	=	=	78	0.0
269	BRCA1-14	17q21.31	17-038.482059	8809	64553	*0.93*	0.03	=	=	56	-0.1
459	BRCA1-13	17q21.31	17-038.487745	5016	46872	*0.94*	0.03	=	=	66	0.0
202	BRCA1-13	17q21.31	17-038.487951	7866	52091	*0.97*	0.03	=	=	42	0.0
301	BRCA1-13	17q21.31	17-038.488025	6881	52280	*0.94*	0.05	=	=	70	0.0
358	BRCA1-12	17q21.31	17-038.496486	6303	52023	*0.97*	0.02	=	=	84	0.0
142	BRCA1-11	17q21.31	17-038.497036	10803	72646	*0.96*	0.03	=	=	71	0.0
281	BRCA1-11	17q21.31	17-038.497460	6470	46627	*0.98*	0.05	=	=	48	0.0
427	BRCA1-11	17q21.31	17-038.498001	5083	47000	*0.95*	0.03	=	=	72	0.0
340	BRCA1-11	17q21.31	17-038.498514	6502	51514	*0.98*	0.04	=	=	65	0.1
233	BRCA1-11	17q21.31	17-038.499023	7998	54530	*0.95*	0.03	=	=	53	0.1
296	BRCA1-11	17q21.31	17-038.499436	8457	63561	*0.97*	0.03	=	=	67	-0.1
382	BRCA1-11	17q21.31	17-038.499815	5910	50667	*0.94*	0.03	=	=	67	0.0
263	BRCA1-11	17q21.31	17-038.500273	7511	54067	*0.99*	0.03	=	=	50	0.0
251	BRCA1-10	17q21.31	17-038.501402	8151	55111	*1.01*	0.03	=	=	50	-0.1
238	BRCA1-9	17q21.31	17-038.502751	8273	55730	*0.98*	0.03	=	=	60	0.0
403	BRCA1-8	17q21.31	17-038.505340	5071	45812	*0.95*	0.04	=	=	78	-0.1
220	BRCA1-7	17q21.31	17-038.509662	9257	61873	*0.96*	0.03	=	=	58	0.0
374	BRCA1-6	17q21.31	17-038.510430	6229	52800	*0.96*	0.03	=	=	77	0.0
190	BRCA1-5	17q21.31	17-038.512011	9788	63132	*0.98*	0.03	=	=	60	0.0
421	BRCA1-3	17q21.31	17-038.521268	5411	49164	*0.94*	0.03	=	=	78	0.0
178	BRCA1-2	17q21.31	17-038.529585	10192	67041	*0.97*	0.03	=	=	56	0.1
289	BRCA1-1b	17q21.31	17-038.530571	7376	55921	*0.95*	0.03	=	=	64	0.0
154	BRCA1-1a	17q21.31	17-038.530812	10755	69468	*0.95*	0.03	=	=	50	0.0
166	BRCA1-up	17q21.31	17-038.531706	9635	63568	*0.96*	0.03	=	=	53	0.0
324	BRCA1-up	17q21.31	17-038.535583	6808	53655	*0.93*	0.03	=	=	66	-0.1
275	STIL*	01p33	01-047.538303	8284	60971	*1.03*	0.03	=	=	57	-0.1
449	EDARADD*	01q42.3	01-234.657310	3673	34662	*0.98*	0.03	=	=	79	0.1
469	COL6A3*	02q37.3	02-237.961415	4476	43234	*1.01*	0.03	=	=	65	0.0
208	PIK3CA*	03q26.32	03-180.399606	8367	53395	*0.99*	0.03	=	=	44	0.0
130	IL4*	05q31.1	05-132.037607	10759	68799	*0.99*	0.03	=	=	39	0.0
316	SIM1*	06q16.3	06-101.001940	6660	49893	*1.03*	0.04	=	=	63	0.1
366	CHD7*	08q12.2	08-061.927614	6437	54176	*1*	0.03	=	=	67	0.0
244	ATP7B*	13q14.3	13-051.416301	8032	56825	*0.97*	0.04	=	=	61	-0.1
136	FBN1*	15q21.1	15-046.501165	11455	74944	*1.01*	0.03	=	=	56	0.0
172	PMAIP1*	18q21.32	18-055.720855	8395	56712	*0.96*	0.02	=	=	55	0.0
**Median value all probe values:**				**7164**	**53525**	**0.96**	**0.03**			**60.5**	**0**

Figure (xiii) : MLPA results showing BRCA2:c.(?_-21)_(67+21_68-21)del

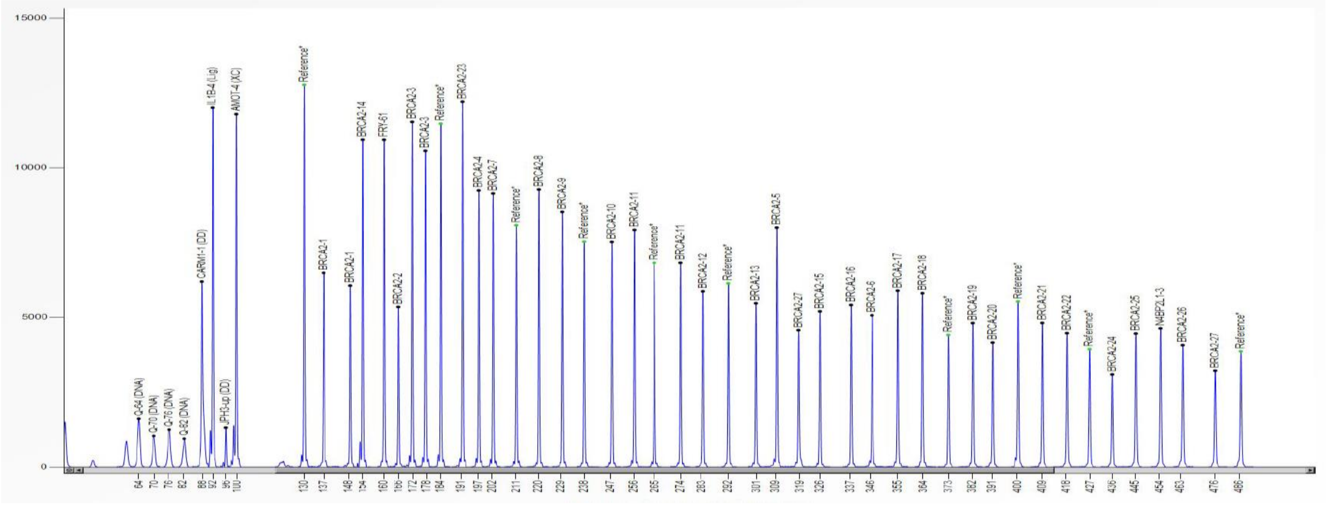

D [nt]	Gene-Exon	Chr.band	hg18 loc.	Height	Area	Ratio[H]	Stdev	[REF]	[Sam]	Width	d[nt]
160	FRY-61	13q13.1	13-031.767521	10939	65119	1	0.04	=	=	54	0.0
137	BRCA2-1	13q13.1	13-031.787593	6491	39872	0.53	0.03	<<*	=	53	0.0
148	BRCA2-1	13q13.1	13-031.787801	6065	37463	0.51	0.02	<<*	=	50	-0.1
166	BRCA2-2	13q13.1	13-031.788599	5352	31977	0.49	0.02	<<*	=	36	0.0
178	BRCA2-3	13q13.1	13-031.791351	10570	63555	0.99	0.04	=	=	48	0.0
172	BRCA2-3	13q13.1	13-031.791539	11538	68179	1.01	0.04	=	=	39	0.0
197	BRCA2-4	13q13.1	13-031.797209	9245	56352	0.93	0.04	=	=	63	0.0
309	BRCA2-5	13q13.1	13-031.798240	8003	55059	1.08	0.05	=	=	52	-0.1
346	BRCA2-6	13q13.1	13-031.798360	5072	36152	1.02	0.04	=	=	56	0.0
202	BRCA2-7	13q13.1	13-031.798677	9145	55465	0.95	0.05	=	=	49	0.0
220	BRCA2-8	13q13.1	13-031.801290	9278	56633	1	0.04	=	=	51	-0.1
229	BRCA2-9	13q13.1	13-031.803112	8529	52486	0.98	0.05	=	=	51	0.0
247	BRCA2-10	13q13.1	13-031.804736	7525	47446	0.99	0.06	=	=	55	0.0
256	BRCA2-11	13q13.1	13-031.808425	7924	50791	1.06	0.04	=	=	58	-0.1
274	BRCA2-11	13q13.1	13-031.813228	6828	43503	1.03	0.04	=	=	58	-0.1
283	BRCA2-12	13q13.1	13-031.816481	5874	39132	0.99	0.05	=	=	59	-0.1
301	BRCA2-13	13q13.1	13-031.818973	5474	36941	0.97	0.05	=	=	42	-0.2
154	BRCA2-14	13q13.1	13-031.827123	10942	68470	0.94	0.05	=	=	33	-0.1
326	BRCA2-15	13q13.1	13-031.828641	5203	34765	0.93	0.04	=	=	48	-0.1
337	BRCA2-16	13q13.1	13-031.829982	5419	37581	0.97	0.04	=	=	62	-0.1
355	BRCA2-17	13q13.1	13-031.834744	5896	41452	1.06	0.05	=	=	49	-0.1
364	BRCA2-18	13q13.1	13-031.835565	5811	42623	1.03	0.05	=	=	50	-0.1
382	BRCA2-19	13q13.1	13-031.842558	4814	35298	0.99	0.04	=	=	56	0.0
391	BRCA2-20	13q13.1	13-031.843090	4161	30761	1	0.05	=	=	59	0.0
409	BRCA2-21	13q13.1	13-031.848828	4818	35674	1.02	0.05	=	=	48	0.1
418	BRCA2-22	13q13.1	13-031.851548	4476	34416	1.02	0.04	=	=	49	0.1
191	BRCA2-23	13q13.1	13-031.851892	12211	73200	1	0.05	=	=	54	0.0
436	BRCA2-24	13q13.1	13-031.852214	3097	24152	1.01	0.04	=	=	43	0.0
445	BRCA2-25	13q13.1	13-031.867023	4462	35818	1.02	0.05	=	=	60	0.0
463	BRCA2-26	13q13.1	13-031.869059	4075	33588	1	0.06	=	=	61	0.0
476	BRCA2-27	13q13.1	13-031.870386	3221	26975	1.03	0.05	=	=	52	0.0
319	BRCA2-27	13q13.1	13-031.871035	4576	33500	1.07	0.05	=	=	50	0.0
454	N4BP2L1-3	13q13.1	13-031.879392	4635	37467	1.07	0.06	=	=	54	0.0
238	Reference*	02q13	02-113.606703	7535	47179	0.98	0.04	=	=	63	-0.1
486	Reference*	02q32.2	02-189.584681	3868	32859	1.16	0.06	=	=	56	0.0
184	Reference*	04q35.2	04-187.390323	11478	67942	1.05	0.05	=	=	51	0.0
130	Reference*	05q31.1	05-132.037607	12777	75432	1.01	0.05	=	=	55	-0.1
427	Reference*	11q12.3	11-061.486331	3941	31039	0.92	0.05	=	=	53	0.0
373	Reference*	11q22.3	11-107.655436	4417	31517	1.01	0.06	=	=	42	0.0
292	Reference*	12q13.13	12-050.594518	6141	40753	0.93	0.04	=	=	62	0.0
211	Reference*	12q23.2	12-101.762243	8083	49695	0.99	0.04	=	=	56	0.0
400	Reference*	13q14.2	13-049.576833	5535	43013	1.01	0.04	=	=	59	0.1
265	Reference*	19p13.2	19-011.079136	6827	43260	0.99	0.06	=	=	48	-0.1
**Median value all probe values:**				**5896**	**40753**	**1**	**0.05**			**53**	**-0.03**

Figure (xiv) : MLPA results showing BRCA2:c.(8487+21_8488-21)_(8632+21_8633-21)del

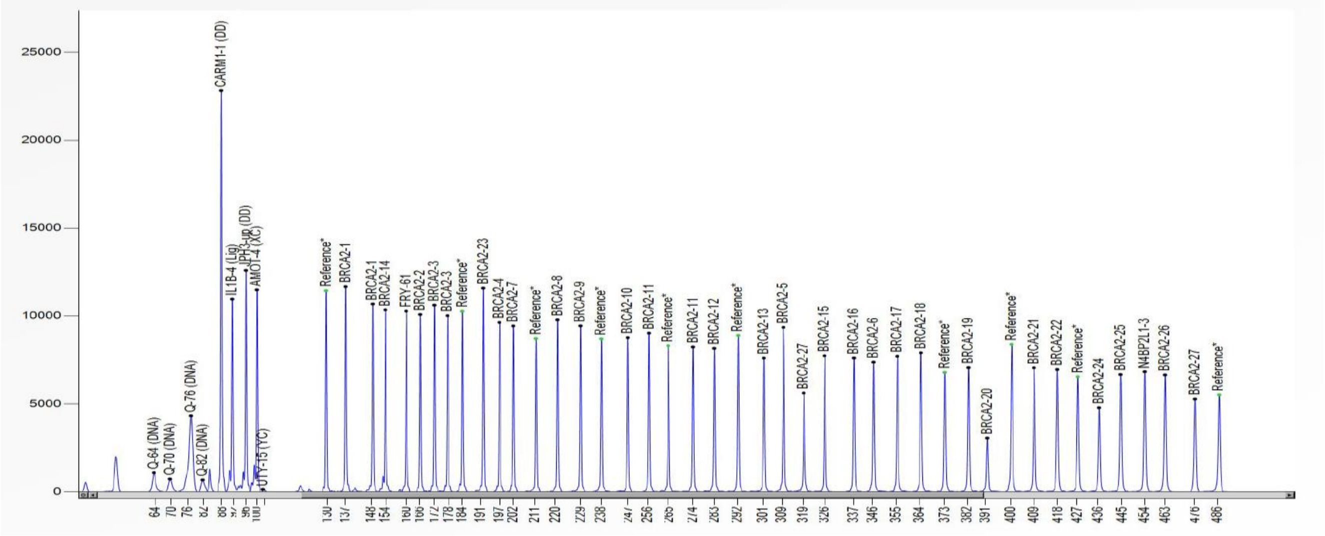

D [nt]	Gene-Exon	Chr.band	hg18 loc.	Height	Area	Ratio	Stdev	[REF]	[Sam]	Width	d[nt]
160	FRY-61	13q13.1	13-031.767521	10282	66604	*0.92*	0.06	=	=	36	0.1
137	BRCA2-1	13q13.1	13-031.787593	11674	75536	*0.96*	0.06	=	=	48	0.0
148	BRCA2-1	13q13.1	13-031.787801	10683	71006	*0.92*	0.06	=	=	47	0.0
166	BRCA2-2	13q13.1	13-031.788599	10088	65720	*0.91*	0.06	=	=	40	0.0
178	BRCA2-3	13q13.1	13-031.791351	10020	65439	*0.94*	0.06	=	=	46	0.0
172	BRCA2-3	13q13.1	13-031.791539	10616	68850	*0.93*	0.07	=	=	36	0.1
197	BRCA2-4	13q13.1	13-031.797209	9637	63828	*0.98*	0.06	=	=	53	0.0
309	BRCA2-5	13q13.1	13-031.798240	9356	73060	*0.93*	0.04	=	=	57	-0.1
346	BRCA2-6	13q13.1	13-031.798360	7375	60287	*1.02*	0.05	=	=	59	0.0
202	BRCA2-7	13q13.1	13-031.798677	9438	63332	*0.95*	0.05	=	=	50	0.0
220	BRCA2-8	13q13.1	13-031.801290	9787	65474	*0.99*	0.04	=	=	69	0.0
229	BRCA2-9	13q13.1	13-031.803112	9441	63735	*0.97*	0.06	=	=	46	0.0
247	BRCA2-10	13q13.1	13-031.804736	8767	60176	*0.96*	0.05	=	=	35	0.0
256	BRCA2-11	13q13.1	13-031.808425	9026	63622	*0.97*	0.04	=	=	56	0.0
274	BRCA2-11	13q13.1	13-031.813228	8239	59109	*0.97*	0.05	=	=	43	0.0
283	BRCA2-12	13q13.1	13-031.816481	8160	59979	*0.98*	0.05	=	=	45	0.0
301	BRCA2-13	13q13.1	13-031.818973	7607	57932	*0.99*	0.04	=	=	55	0.1
154	BRCA2-14	13q13.1	13-031.827123	10350	72885	*0.9*	0.07	=	=	58	0.0
326	BRCA2-15	13q13.1	13-031.828641	7736	58959	*1*	0.05	=	=	44	0.1
337	BRCA2-16	13q13.1	13-031.829982	7612	61768	*0.98*	0.06	=	=	72	-0.1
355	BRCA2-17	13q13.1	13-031.834744	7708	64155	*0.97*	0.05	=	=	56	-0.1
364	BRCA2-18	13q13.1	13-031.835565	7906	66564	*1.01*	0.05	=	=	60	-0.1
382	BRCA2-19	13q13.1	13-031.842558	7065	62033	*0.98*	0.05	=	=	58	-0.1
391	BRCA2-20	13q13.1	13-031.843090	3052	27746	***0.52***	0.03	<<*	=	55	-0.1
409	BRCA2-21	13q13.1	13-031.848828	7054	66285	*1.02*	0.05	=	=	82	-0.1
418	BRCA2-22	13q13.1	13-031.851548	6958	63723	*1.04*	0.07	=	=	50	-0.1
191	BRCA2-23	13q13.1	13-031.851892	11590	76837	*0.94*	0.06	=	=	57	0.0
436	BRCA2-24	13q13.1	13-031.852214	4781	46119	*1.03*	0.08	=	=	78	0.0
445	BRCA2-25	13q13.1	13-031.867023	6671	64631	*1.01*	0.07	=	=	63	0.1
463	BRCA2-26	13q13.1	13-031.869059	6648	65719	*1.01*	0.07	=	=	83	0.0
476	BRCA2-27	13q13.1	13-031.870386	5275	52609	*1.05*	**0.11**	=	=	42	0.0
319	BRCA2-27	13q13.1	13-031.871035	5618	47473	*0.97*	0.05	=	=	70	0.0
454	N4BP2L1-3	13q13.1	13-031.879392	6834	66586	*1.02*	0.08	=	=	77	-0.1
238	Reference*	02q13	02-113.606703	8701	61848	*0.98*	0.05	=	=	76	0.1
486	Reference*	02q32.2	02-189.584681	5518	58433	*1.03*	0.1	=	=	56	0.0
184	Reference*	04q35.2	04-187.390323	10278	67828	*0.96*	0.06	=	=	50	0.0
130	Reference*	05q31.1	05-132.037607	11443	72326	*0.96*	0.08	=	=	43	-0.1
427	Reference*	11q12.3	11-061.486331	6547	61745	*1.02*	0.07	=	=	78	-0.1
373	Reference*	11q22.3	11-107.655436	6792	57286	*1.06*	0.07	=	=	53	0.0
292	Reference*	12q13.13	12-050.594518	8897	66947	*1.01*	0.05	=	=	46	-0.1
211	Reference*	12q23.2	12-101.762243	8721	58214	*0.98*	0.05	=	=	50	0.0
400	Reference*	13q14.2	13-049.576833	8389	76044	*1.02*	0.05	=	=	61	0.0
265	Reference*	19p13.2	19-011.079136	8310	58879	*1*	0.06	=	=	37	0.0
**Median value all probe values:**				**8310**	**63735**	**0.98**	**0.06**			**55**	**-0.02**

Figure (xv) : MLPA results showing CHEK2:c.(1461+21_1462-21)_(*21_?)del

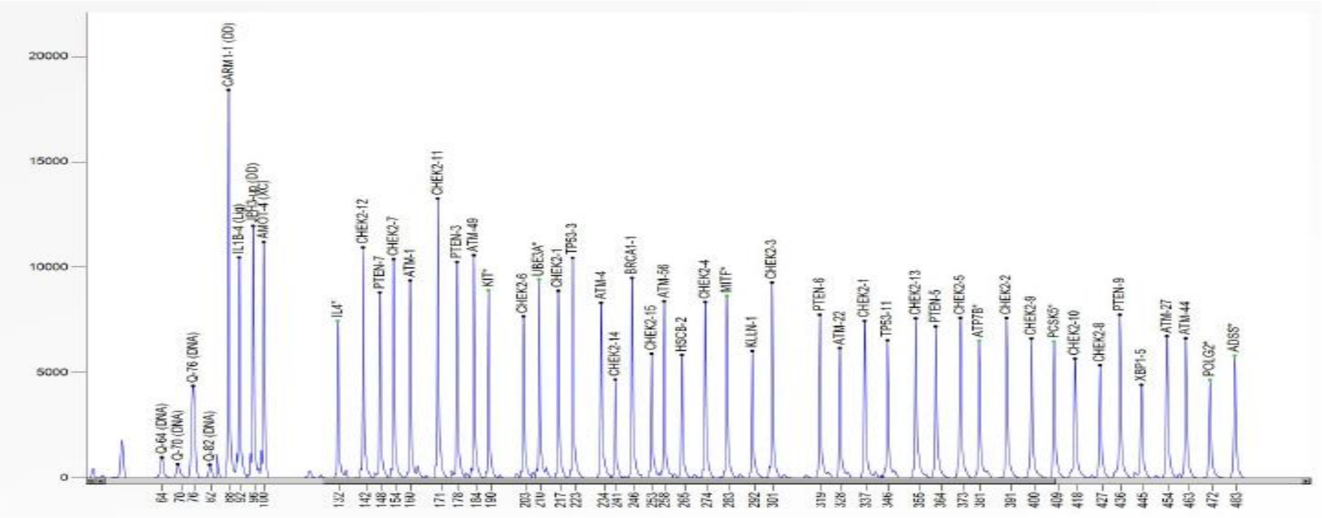

D [nt]	Gene-Exon	Chr.band	hg18 loc.	Height	Area	Ratio	Stdev	[REF]	[Sam]	Width	d[nt]
292	KLLN-1	10q23.31	10-089.612348	6024	58171	*1.02*	0.05	=	=	72	0.0
178	PTEN-3	10q23.31	10-089.675483	10248	87654	*0.97*	0.04	=	=	50	0.0
364	PTEN-5	10q23.31	10-089.682864	7191	80027	*0.95*	0.03	=	=	89	0.0
319	PTEN-6	10q23.31	10-089.701944	7744	75144	*0.98*	0.04	=	=	47	0.0
148	PTEN-7	10q23.31	10-089.707638	8807	73486	*1.04*	0.04	=	=	54	0.0
436	PTEN-9	10q23.31	10-089.716113	7745	92594	*0.96*	0.03	=	=	88	0.0
160	ATM-1	11q22.3	11-107.598963	9370	78706	*0.98*	0.06	=	=	50	-0.1
234	ATM-4	11q22.3	11-107.605158	8310	77036	*0.97*	0.05	=	=	66	0.0
328	ATM-22	11q22.3	11-107.648710	6166	59187	*1*	0.05	=	=	46	0.0
454	ATM-27	11q22.3	11-107.663555	6728	80316	*0.98*	0.03	=	=	64	-0.1
463	ATM-44	11q22.3	11-107.695883	6625	78227	*0.96*	0.03	=	=	67	0.0
184	ATM-49	11q22.3	11-107.705086	10573	90676	*0.97*	0.04	=	=	64	0.0
258	ATM-56	11q22.3	11-107.711788	8385	77276	*1*	0.03	=	=	65	0.1
346	TP53-11	17p13.1	17-007.513675	6528	67229	*0.96*	0.03	=	=	46	0.0
223	TP53-3	17p13.1	17-007.520274	10445	94496	*0.97*	0.04	=	=	68	0.1
246	BRCA1-1	17q21.31	17-038.530812	9492	84206	*0.98*	0.03	=	=	52	0.0
253	CHEK2-15	22q12.1	22-027.413871	5898	55245	***0.52***	0.02	<<*	?	49	0.0
241	CHEK2-14	22q12.1	22-027.414887	4665	39986	***0.52***	0.02	<<*	?	42	0.0
355	CHEK2-13	22q12.1	22-027.420111	7575	88453	*0.93*	0.03	=	=	93	0.0
142	CHEK2-12	22q12.1	22-027.421039	10942	90930	*1.02*	0.03	=	=	62	0.0
171	CHEK2-11	22q12.1	22-027.421646	13265	112691	*0.97*	0.03	=	=	64	0.0
418	CHEK2-10	22q12.1	22-027.422916	5660	71505	*0.95*	0.04	=	=	93	0.0
400	CHEK2-9	22q12.1	22-027.425857	6615	77880	*1.02*	0.03	=	=	108	0.0
427	CHEK2-8	22q12.1	22-027.429486	5354	61981	*0.99*	0.04	=	=	63	0.0
154	CHEK2-7	22q12.1	22-027.435971	10385	88373	*0.95*	0.03	=	=	64	0.0
203	CHEK2-6	22q12.1	22-027.437923	7671	66770	*0.94*	0.03	=	=	52	0.1
373	CHEK2-5	22q12.1	22-027.445376	7600	89235	*0.96*	0.03	=	=	89	0.0
274	CHEK2-4	22q12.1	22-027.451000	8350	77652	*0.98*	0.04	=	=	59	0.0
301	CHEK2-3	22q12.1	22-027.451240	9274	88960	*0.96*	0.03	=	=	70	0.0
391	CHEK2-2	22q12.1	22-027.460460	7595	84909	*1*	0.03	=	=	87	0.0
337	CHEK2-1	22q12.1	22-027.467771	7456	75202	*1*	0.03	=	=	59	0.0
217	CHEK2-1	22q12.1	22-027.467977	8884	76777	*0.99*	0.04	=	=	61	0.1
265	HSCB-2	22q12.1	22-027.469904	5833	51599	*0.97*	0.05	=	=	58	0.0
445	XBP1-5	22q12.1	22-027.521634	4420	52623	*0.96*	0.03	=	=	57	0.0
310	CHEK2-11 (MUT	22q12.1	22-027.421828	**0**	0	**0**	0	**<<****	<<**	**0**	0.0
483	ADSS*	01q44	01-242.653849	5809	70794	*0.97*	0.03	=	=	67	0.0
283	MITF*	03p13	03-070.088343	8638	79995	*1.01*	0.03	=	=	60	0.0
190	KIT*	04q12	04-055.259455	8909	74942	*1*	0.03	=	=	60	0.1
132	IL4*	05q31.1	05-132.037610	7458	58902	*1.03*	0.04	=	=	45	0.0
409	PCSK5*	09q21.13	09-078.032269	6473	73623	*0.97*	0.03	=	=	74	0.0
381	ATP7B*	13q14.3	13-051.413247	6515	69584	*1.05*	0.04	=	=	43	-0.1
210	UBE3A*	15q11.2	15-023.135402	9424	80117	*1.01*	0.02	=	=	40	0.0
472	POLG2*	17q23.3	17-059.919503	4651	56470	*0.98*	0.03	=	=	100	0.0
**Median value all probe values:**				**7598**	**77156**	**0.98**	**0.03**			**62.5**	**0**

Figure (xvi) : MLPA results showing CHEK2:c.(319+21_320-21)_(592+21_593-21)dup

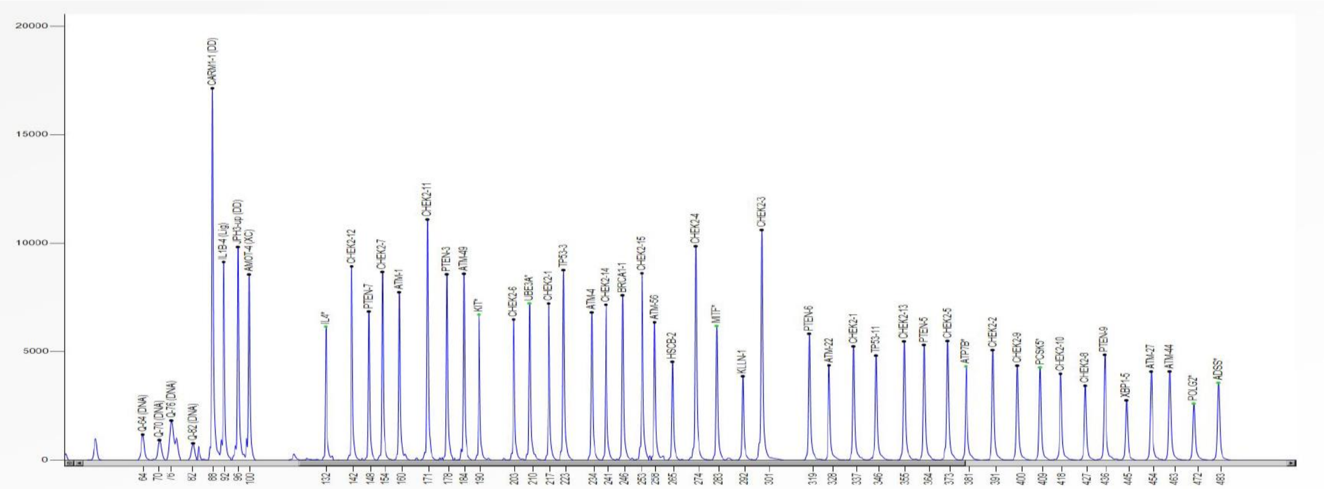

D [nt]	Gene-Exon	Chr.band	hg18 loc.	Height	Area	Ratio H	Stdev	[REF]	[Sam]	Width	d[nt]
292	KLLN-1	10q23.31	10-089.612348	3862	35205	0.97	0.09	=	=	67	0.0
178	PTEN-3	10q23.31	10-089.675483	8566	64575	1.01	0.07	=	=	39	-0.1
364	PTEN-5	10q23.31	10-089.682864	5308	54038	1	0.08	=	=	76	0.0
319	PTEN-6	10q23.31	10-089.701944	5823	55522	1.11	0.23	=	=	84	0.0
148	PTEN-7	10q23.31	10-089.707638	6849	51827	0.99	0.08	=	=	49	0.0
436	PTEN-9	10q23.31	10-089.716113	4853	54444	0.99	0.13	=	=	72	0.0
160	ATM-1	11q22.3	11-107.598963	7737	62943	1.12	0.18	=	=	63	0.0
234	ATM-4	11q22.3	11-107.605158	6806	56962	1.03	0.08	=	=	49	0.0
328	ATM-22	11q22.3	11-107.648710	4365	42052	1.08	0.13	=	=	62	-0.1
454	ATM-27	11q22.3	11-107.663555	4079	47351	0.99	0.08	=	=	75	0.1
463	ATM-44	11q22.3	11-107.695883	4084	47190	0.99	0.09	=	=	75	0.1
184	ATM-49	11q22.3	11-107.705086	8591	68806	1.14	0.22	=	=	58	-0.1
258	ATM-56	11q22.3	11-107.711788	6351	52642	0.93	0.07	=	=	37	0.0
346	TP53-11	17p13.1	17-007.513675	4817	51794	1.26	0.5	=	=	86	-0.1
223	TP53-3	17p13.1	17-007.520274	8760	73462	1.05	0.08	=	=	56	-0.1
246	BRCA1-1	17q21.31	17-038.530812	7597	62822	0.97	0.07	=	=	47	0.0
253	CHEK2-15	22q12.1	22-027.413871	8611	76596	0.93	0.09	=	=	50	0.0
241	CHEK2-14	22q12.1	22-027.414887	7161	58617	1.03	0.08	=	=	51	0.0
355	CHEK2-13	22q12.1	22-027.420111	5470	56387	1.05	0.12	=	=	65	0.0
142	CHEK2-12	22q12.1	22-027.421039	8930	67758	1.02	0.07	=	=	54	0.0
171	CHEK2-11	22q12.1	22-027.421646	11093	85827	1.03	0.08	=	=	64	-0.1
418	CHEK2-10	22q12.1	22-027.422916	3978	44410	1.02	0.11	=	=	76	0.1
400	CHEK2-9	22q12.1	22-027.425857	4351	46463	1.07	0.09	=	=	75	0.0
427	CHEK2-8	22q12.1	22-027.429486	3426	37802	1.03	0.07	=	=	67	0.0
154	CHEK2-7	22q12.1	22-027.435971	8676	68413	1	0.07	=	=	63	0.0
203	CHEK2-6	22q12.1	22-027.437923	6481	52802	1	0.08	=	=	45	0.0
373	CHEK2-5	22q12.1	22-027.445376	5490	59886	0.94	0.08	=	=	76	-0.1
274	CHEK2-4	22q12.1	22-027.451000	9859	89918	1.43	0.1	>>*	?	96	0.0
301	CHEK2-3	22q12.1	22-027.451240	10610	101978	1.43	0.13	>>*	?	117	0.0
391	CHEK2-2	22q12.1	22-027.460460	5070	52932	1.05	0.08	=	=	66	0.0
337	CHEK2-1	22q12.1	22-027.467771	5240	52557	0.89	0.1	=	=	76	-0.1
217	CHEK2-1	22q12.1	22-027.467977	7218	58808	1	0.07	=	=	59	0.0
265	HSCB-2	22q12.1	22-027.469904	4531	38511	1.11	0.32	=	=	50	0.0
445	XBP1-5	22q12.1	22-027.521634	2751	30859	1.09	0.15	=	=	57	0.0
310	CHEK2-11 (MUT	22q12.1	22-027.421828	0	0	0	0	<<**	<<**	0	0.0
483	ADSS*	01q44	01-242.653849	3562	42076	1.21	0.33	=	=	74	0.1
283	MITF*	03p13	03-070.088343	6183	54435	0.92	0.08	=	=	56	0.0
190	KIT*	04q12	04-055.259455	6709	53643	1.03	0.14	=	=	49	0.0
132	IL4*	05q31.1	05-132.037610	6150	46636	1.03	0.07	=	=	37	0.0
409	PCSK5*	09q21.13	09-078.032269	4271	45147	1	0.09	=	=	71	0.0
381	ATP7B*	13q14.3	13-051.413247	4321	46340	0.99	0.08	=	=	79	0.0
210	UBE3A*	15q11.2	15-023.135402	7226	60167	0.98	0.08	=	=	59	-0.1
472	POLG2*	17q23.3	17-059.919503	2604	31985	0.98	0.07	=	=	75	0.1
**Median value all probe values:**				**5986**	**53840**	**1.02**	**0.08**			**63.5**	**-0.01**

Figure (xvii): MLPA results showing CHEK2: c.(908+21_909-21)_(1095+21_1096-21)del

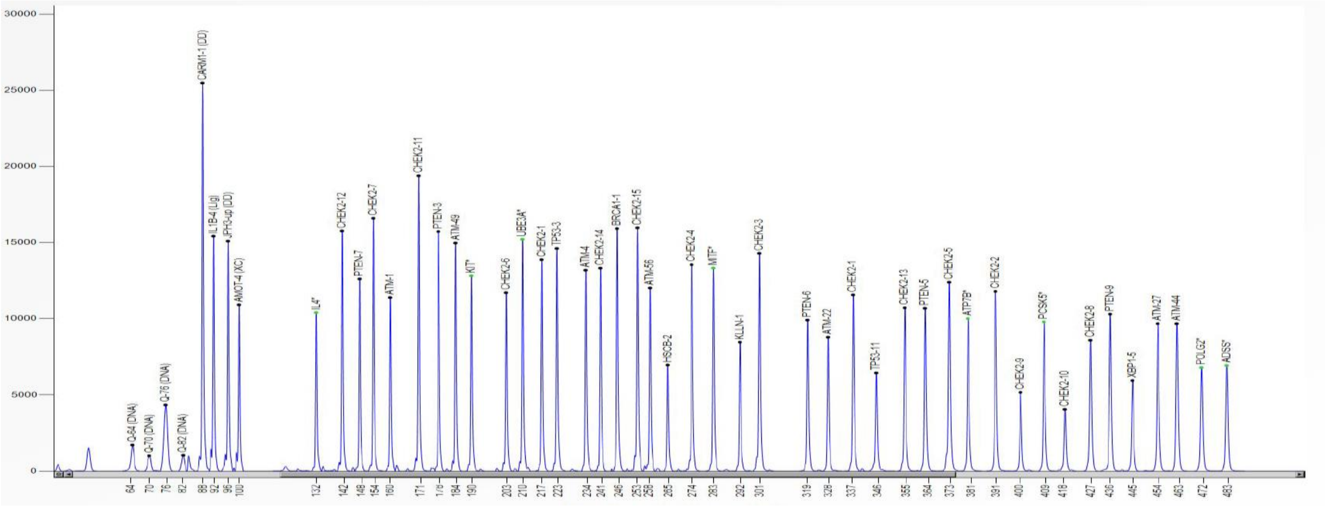

D [nt]	Gene-Exon	Chr.band	hg18 loc.	Height	Area	Ratio[H]	Stdev	[REF]	[Sam]	Width	d[nt]
292	KLLN-1	10q23.31	10-089.612348	8465	70934	1.06	0.07	=	=	51	0.0
178	PTEN-3	10q23.31	10-089.675483	15727	111349	0.96	0.06	=	=	34	0.0
364	PTEN-5	10q23.31	10-089.682864	10691	99376	0.93	0.05	=	=	68	0.0
319	PTEN-6	10q23.31	10-089.701944	9916	85695	0.95	**0.11**	=	=	64	0.0
148	PTEN-7	10q23.31	10-089.707638	12620	88115	0.91	0.05	=	=	47	0.0
436	PTEN-9	10q23.31	10-089.716113	10306	104791	0.98	0.09	=	=	78	0.0
160	ATM-1	11q22.3	11-107.598963	11395	81409	0.95	**0.14**	=	=	39	0.0
234	ATM-4	11q22.3	11-107.605158	13189	102628	0.96	0.06	=	=	49	0.1
328	ATM-22	11q22.3	11-107.648710	8790	74708	1.03	**0.12**	=	=	40	0.0
454	ATM-27	11q22.3	11-107.663555	9683	101595	0.99	0.05	=	=	61	0.1
463	ATM-44	11q22.3	11-107.695883	9678	101971	0.9	0.07	=	=	61	0.1
184	ATM-49	11q22.3	11-107.705086	14975	108222	1	0.1	=	=	41	-0.1
258	ATM-56	11q22.3	11-107.711788	12023	96676	0.95	0.05	=	=	49	-1.3
346	TP53-11	17p13.1	17-007.513675	6447	61633	0.94	**0.24**	=	=	74	0.0
223	TP53-3	17p13.1	17-007.520274	14616	111468	0.89	0.05	=	=	60	0.0
246	BRCA1-1	17q21.31	17-038.530812	15921	123795	1.02	0.05	=	=	46	-1.2
253	CHEK2-15	22q12.1	22-027.413871	15972	127503	0.94	0.05	=	=	47	-1.6
241	CHEK2-14	22q12.1	22-027.414887	13324	100593	0.99	0.07	=	=	43	0.1
355	CHEK2-13	22q12.1	22-027.420111	10720	102883	0.97	0.08	=	=	69	-0.1
142	CHEK2-12	22q12.1	22-027.421039	15765	111600	0.92	0.05	=	=	51	0.1
171	CHEK2-11	22q12.1	22-027.421646	19390	140867	0.91	0.05	=	=	66	-0.1
418	CHEK2-10	22q12.1	22-027.422916	4053	42328	**0.51**	0.04	<<*	=	52	0.0
400	CHEK2-9	22q12.1	22-027.425857	5172	49349	**0.52**	0.03	<<*	=	54	0.0
427	CHEK2-8	22q12.1	22-027.429486	8592	86634	1	0.06	=	=	57	0.1
154	CHEK2-7	22q12.1	22-027.435971	16597	118072	0.97	0.06	=	=	60	0.0
203	CHEK2-6	22q12.1	22-027.437923	11715	88016	0.94	0.06	=	=	45	0.0
373	CHEK2-5	22q12.1	22-027.445376	12400	124417	0.98	0.06	=	=	78	-0.1
274	CHEK2-4	22q12.1	22-027.451000	13556	112170	0.96	0.07	=	=	59	0.1
301	CHEK2-3	22q12.1	22-027.451240	14298	122101	0.96	0.06	=	=	58	0.0
391	CHEK2-2	22q12.1	22-027.460460	11797	110961	0.98	0.07	=	=	83	0.0
337	CHEK2-1	22q12.1	22-027.467771	11571	103857	1.01	0.07	=	=	61	0.0
217	CHEK2-1	22q12.1	22-027.467977	13879	103765	0.91	0.06	=	=	54	0.1
265	HSCB-2	22q12.1	22-027.469904	6966	54766	0.98	**0.17**	=	=	47	0.0
445	XBP1-5	22q12.1	22-027.521634	5945	60183	1.04	**0.14**	=	=	51	0.0
310	CHEK2-11 (MUT	22q12.1	22-027.421828	**0**	0	**0**	0	<<**	<<**	**0**	0.0
483	ADSS*	01q44	01-242.653849	6936	76234	0.95	**0.11**	=	=	73	0.1
283	MITF*	03p13	03-070.088343	13333	107199	1	0.06	=	=	64	0.0
190	KIT*	04q12	04-055.259455	12830	94517	1.01	0.07	=	=	50	-0.1
132	IL4*	05q31.1	05-132.037610	10409	73098	0.9	0.05	=	=	37	0.0
409	PCSK5*	09q21.13	09-078.032269	9801	94450	1.04	0.06	=	=	68	0.0
381	ATP7B*	13q14.3	13-051.413247	10013	94611	0.93	0.06	=	=	79	0.0
210	UBE3A*	15q11.2	15-023.135402	15218	112834	0.98	0.07	=	=	35	0.1
472	POLG2*	17q23.3	17-059.919503	6802	74162	1.04	0.05	=	=	77	0.1
**Median value all probe values:**				**11643**	**101094**	**0.96**	**0.06**			**55.5**	**0.01**

Figure (xviii): MLPA results showing MLH1:c.(116+21_117-21)_(545+21_546-21)del

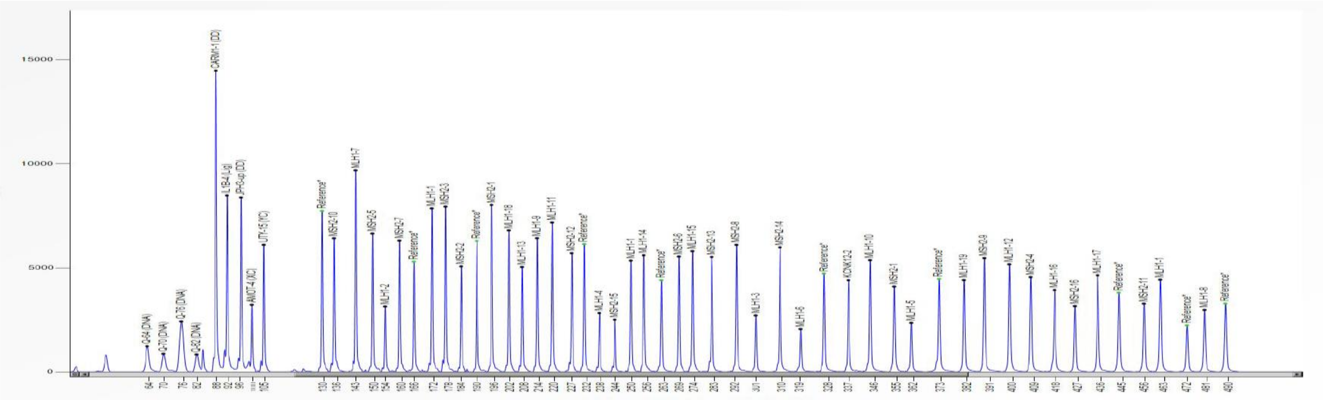

D [nt]	Gene-Exon	Chr.band	hg18 loc.	Height	Area	Ratio ^{H}	Stdev	[REF]	[Sam]	Width	d[nt]
196	MSH2-1	02p21	02-047.483525	8025	57868	*1.01*	0.07	=	=	59	0.0
355	MSH2-1	02p21	02-047.483612	4100	36834	*0.95*	0.1	=	=	68	0.0
184	MSH2-2	02p21	02-047.489038	5071	34933	*0.94*	0.06	=	=	34	0.0
178	MSH2-3	02p21	02-047.490867	7944	58609	*0.98*	0.04	=	=	61	-0.1
409	MSH2-4	02p21	02-047.493144	4555	43938	*1.02*	0.06	=	=	72	0.0
150	MSH2-5	02p21	02-047.494986	6649	47273	*1*	0.06	=	=	53	0.1
269	MSH2-6	02p21	02-047.496841	5543	41967	*0.99*	0.05	=	=	45	0.0
160	MSH2-7	02p21	02-047.510386	6310	43694	*1.01*	0.04	=	=	49	0.0
292	MSH2-8	02p21	02-047.526347	6105	47579	*1.06*	0.04	=	=	61	0.0
391	MSH2-9	02p21	02-047.543721	5468	52008	*1*	0.04	=	=	90	0.0
136	MSH2-10	02p21	02-047.547286	6420	47638	*0.98*	0.04	=	=	58	-0.1
456	MSH2-11	02p21	02-047.551703	3277	32711	*1*	0.06	=	=	55	0.1
227	MSH2-12	02p21	02-047.555667	5705	42953	*1*	0.04	=	=	61	0.0
283	MSH2-13	02p21	02-047.557100	5523	44332	*0.97*	0.04	=	=	63	0.0
310	MSH2-14	02p21	02-047.559068	5985	50276	*1*	0.06	=	=	78	-0.1
244	MSH2-15	02p21	02-047.561316	2507	19428	*0.97*	0.05	=	=	53	0.0
427	MSH2-16	02p21	02-047.563439	3159	30162	*0.86*	0.05	=	=	73	0.0
337	KCNK12-2	02p21	02-047.601937	4405	37566	*0.97*	0.05	=	=	49	0.0
463	MLH1-1	03p22.2	03-037.009473	4430	45678	*0.93*	0.05	=	=	76	0.0
172	MLH1-1	03p22.2	03-037.010000	7858	56971	*1.02*	0.08	=	=	57	0.1
250	MLH1-1	03p22.2	03-037.010126	5348	40927	*0.95*	0.08	=	=	61	0.0
154	MLH1-2	03p22.2	03-037.012853	3141	22088	***0.56***	0.03	<<*	=	33	0.0
301	MLH1-3	03p22.2	03-037.017529	2712	20964	***0.52***	0.02	<<*	=	36	0.0
238	MLH1-4	03p22.2	03-037.020909	2827	21195	***0.48***	0.02	<<*	=	49	0.0
362	MLH1-5	03p22.2	03-037.023534	2354	21382	***0.5***	0.02	<<*	=	63	0.0
319	MLH1-6	03p22.2	03-037.025325	2056	17578	***0.52***	0.03	<<*	=	43	0.1
143	MLH1-7	03p22.2	03-037.028372	9690	68657	*1.09*	0.04	=	=	61	0.0
481	MLH1-8	03p22.2	03-037.028660	2976	30627	*1.05*	0.07	=	=	71	0.0
214	MLH1-9	03p22.2	03-037.031058	6425	46188	*1*	0.04	=	=	52	0.0
346	MLH1-10	03p22.2	03-037.034042	5369	47969	*1.06*	0.05	=	=	90	0.0
220	MLH1-11	03p22.2	03-037.036956	7181	51132	*1.05*	0.04	=	=	51	0.0
400	MLH1-12	03p22.2	03-037.042143	5170	49535	*1.01*	0.07	=	=	77	0.0
208	MLH1-13	03p22.2	03-037.045357	5039	36867	*0.92*	0.03	=	=	50	0.1
256	MLH1-14	03p22.2	03-037.056781	5605	44148	*1.03*	0.05	=	=	61	0.1
274	MLH1-15	03p22.2	03-037.058844	5805	45260	*1.02*	0.05	=	=	48	0.0
418	MLH1-16	03p22.2	03-037.064056	3931	39778	*1.1*	0.06	=	=	71	0.0
436	MLH1-17	03p22.2	03-037.065022	4642	44420	*1.02*	0.04	=	=	76	0.0
202	MLH1-18	03p22.2	03-037.065492	6800	48020	*1.03*	0.05	=	=	46	0.0
382	MLH1-19	03p22.2	03-037.067101	4411	41490	*0.98*	0.04	=	=	81	0.0
130	Reference*	02p13.2	02-071.750350	7737	53886	*1.05*	0.05	=	=	48	-0.1
445	Reference*	03q26.2	03-170.331898	3807	38343	*0.98*	0.06	=	=	77	0.1
232	Reference*	04p13	04-042.278439	6144	45617	*0.99*	0.05	=	=	59	-0.1
373	Reference*	04q35.2	04-187.444615	4467	44275	*1.02*	0.07	=	=	109	0.0
472	Reference*	07p21.1	07-019.714909	2237	23084	*0.87*	0.06	=	=	70	0.1
166	Reference*	09p24.1	09-006.544621	5299	36807	*0.96*	0.04	=	=	49	0.0
328	Reference*	09q34.11	09-129.477992	4727	41312	*1.03*	0.05	=	=	77	0.0
190	Reference*	12q12	12-039.020354	6292	42381	*1.03*	0.04	=	=	38	-0.1
263	Reference*	17q25.3	17-072.998432	4407	34457	*0.95*	0.09	=	=	68	0.0
490	Reference*	22q12.3	22-035.020256	3282	35639	*1.04*	0.07	=	=	83	0.0
**Median value all probe values:**				**5170**	**42953**	**1**	**0.05**			**61**	**0**

Figure (xix) : MLPA results showing MSH2:c.(?_-21)_(*21_?)del

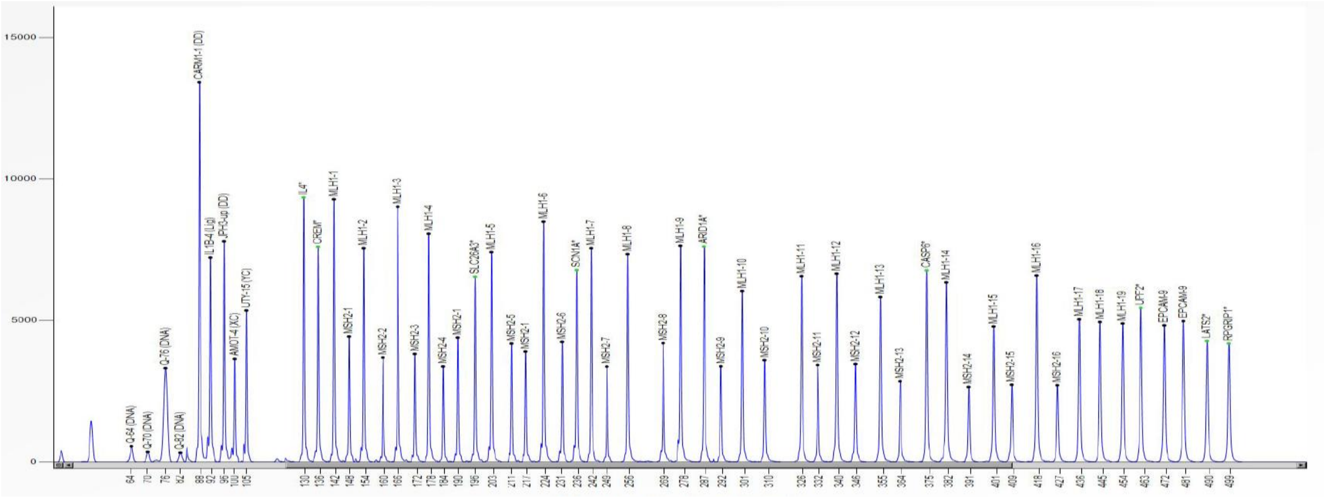

D [nt]	Gene-Exon	Chr.band	hg18 loc.	Height	Area	Ratio^H	Stdev	[REF]	[Sam]	Width	d[nt]
265	MSH2-intr07 (	02p21	02-047.523129	**0**	0	**0**	0	<<**	<<**	**0**	0.0
317	MSH2-intr07 (	02p21	02-047.522602	**0**	0	**0**	0	<<**	<<**	**0**	0.0
481	EPCAM-9	02p21	02-047.467274	4982	55256	*0.98*	0.07	=	=	76	0.0
472	EPCAM-9	02p21	02-047.467390	4828	52317	*0.96*	0.07	=	=	82	0.0
217	MSH2-1	02p21	02-047.483423	3907	30153	***0.52***	0.03	<<*	?	44	0.0
148	MSH2-1	02p21	02-047.483612	4434	31742	***0.53***	0.04	<<*	?	39	0.0
190	MSH2-1	02p21	02-047.484274	4396	32597	***0.55***	0.05	<<*	?	44	0.0
160	MSH2-2	02p21	02-047.489105	3692	26409	***0.54***	0.04	<<*	?	45	0.0
172	MSH2-3	02p21	02-047.490794	3821	28295	***0.57***	0.04	<<*	?	57	0.0
184	MSH2-4	02p21	02-047.493125	3377	23821	***0.55***	0.03	<<*	?	37	0.0
211	MSH2-5	02p21	02-047.494910	4189	31496	***0.53***	0.04	<<*	?	39	0.0
231	MSH2-6	02p21	02-047.496965	4248	34006	***0.53***	0.04	<<*	?	66	0.0
249	MSH2-7	02p21	02-047.510479	3374	24970	***0.53***	0.04	<<*	?	44	0.0
269	MSH2-8	02p21	02-047.526209	4204	36464	***0.55***	0.03	<<*	?	73	0.0
292	MSH2-9	02p21	02-047.543669	3382	27989	***0.52***	0.03	<<*	?	49	0.3
310	MSH2-10	02p21	02-047.547615	3600	31125	***0.54***	0.03	<<*	?	80	0.0
332	MSH2-11	02p21	02-047.551611	3429	29464	***0.52***	0.03	<<*	?	55	-0.1
346	MSH2-12	02p21	02-047.555737	3463	31844	***0.54***	0.03	<<*	?	69	-0.1
364	MSH2-13	02p21	02-047.557048	2855	27014	***0.52***	0.03	<<*	?	56	0.0
391	MSH2-14	02p21	02-047.558953	2650	25948	***0.49***	0.03	<<*	?	66	0.1
409	MSH2-15	02p21	02-047.561413	2731	28818	***0.52***	0.03	<<*	?	63	0.1
427	MSH2-16	02p21	02-047.563398	2712	28209	***0.52***	0.04	<<*	?	65	0.1
142	MLH1-1	03p22.2	03-037.010019	9284	65234	*1*	0.06	=	=	53	-0.1
154	MLH1-2	03p22.2	03-037.013155	7554	54966	*1.02*	0.08	=	=	57	0.0
166	MLH1-3	03p22.2	03-037.017477	9021	65672	*1.07*	0.09	=	=	54	-0.1
178	MLH1-4	03p22.2	03-037.020894	8068	57628	*1.08*	0.08	=	=	60	0.0
203	MLH1-5	03p22.2	03-037.023506	7418	57395	*1.08*	0.07	=	=	70	0.0
224	MLH1-6	03p22.2	03-037.025297	8494	66458	*1.06*	0.07	=	=	68	0.0
242	MLH1-7	03p22.2	03-037.028310	7554	56532	*1.06*	0.07	=	=	56	-0.1
256	MLH1-8	03p22.2	03-037.028508	7345	57530	*1.06*	0.07	=	=	74	0.0
278	MLH1-9	03p22.2	03-037.030942	7641	64384	*1.04*	0.07	=	=	87	0.0
301	MLH1-10	03p22.2	03-037.034002	6041	49812	*1.04*	0.06	=	=	68	-0.1
326	MLH1-11	03p22.2	03-037.036886	6566	56271	*1.04*	0.06	=	=	67	0.0
340	MLH1-12	03p22.2	03-037.042354	6656	60042	*1*	0.06	=	=	68	0.0
355	MLH1-13	03p22.2	03-037.045306	5831	53499	*1.01*	0.06	=	=	75	0.1
382	MLH1-14	03p22.2	03-037.056694	6348	63620	*1.05*	0.06	=	=	80	0.0
401	MLH1-15	03p22.2	03-037.058759	4789	47561	*1.02*	0.06	=	=	65	0.0
418	MLH1-16	03p22.2	03-037.064022	6589	64474	*1.01*	0.06	=	=	68	0.1
436	MLH1-17	03p22.2	03-037.065060	5045	52755	*0.94*	0.05	=	=	69	0.0
445	MLH1-18	03p22.2	03-037.065438	4950	51060	*1.01*	0.06	=	=	73	0.0
454	MLH1-19	03p22.2	03-037.067228	4896	51737	*0.96*	0.06	=	=	76	0.0
287	ARID1A*	01p36.11	01-026.972646	7612	62223	*1.03*	0.07	=	=	57	0.0
236	SCN1A*	02q24.3	02-166.713867	6782	54507	*1.02*	0.06	=	=	65	0.0
375	CASP6*	04q25	04-110.831524	6770	62824	*1*	0.05	=	=	67	0.0
130	IL4*	05q31.1	05-132.037609	9350	65380	*1.02*	0.1	=	=	59	0.0
196	SLC26A3*	07q31.1	07-107.195395	6545	47036	*1.08*	0.07	=	=	41	0.0
463	UPF2*	10p14	10-012.018627	5450	58104	*0.97*	0.08	=	=	79	0.0
136	CREM*	10p11.21	10-035.517226	7602	55683	*1.02*	0.1	=	=	60	0.0
490	LATS2*	13q12.11	13-020.447119	4280	46849	*0.97*	0.06	=	=	83	0.0
499	RPGRIP1*	14q11.2	14-020.859957	4191	49231	*0.93*	0.06	=	=	82	-0.1
**Median value all probe values:**				**4966**	**51398**	**0.98**	**0.06**			**65.5**	**-0.01**

Figure (xx) : MLPA results showing MSH2:c.(366+21_367-21)_(1076+21_1077-21)del

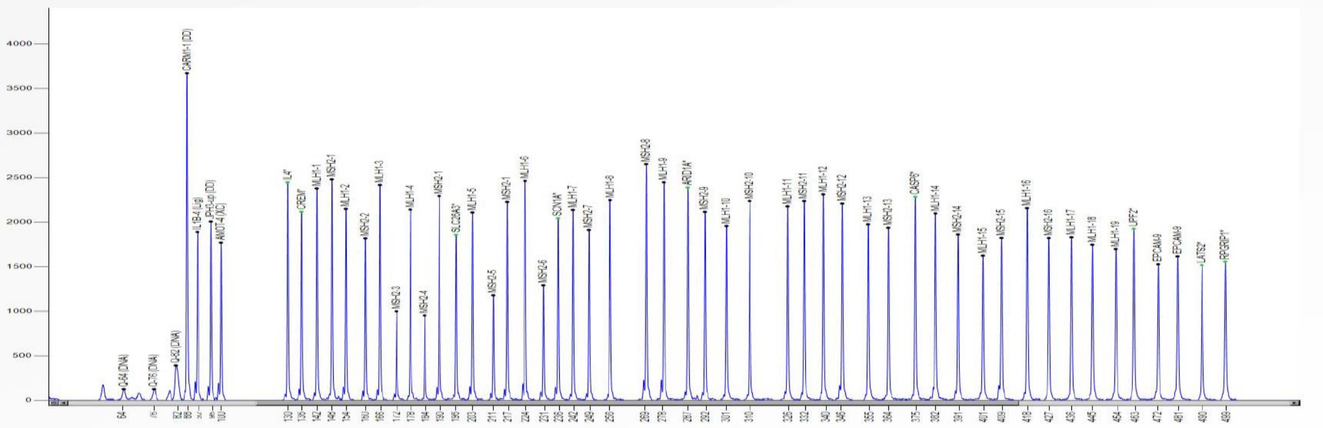

D [nt]	Gene-Exon	Chr.band	hg18 loc.	Height	Area	Ratio[H]	Stdev	[REF]	[Sam]	Width	d[nt]
265	MSH2-intr07 (	02p21	02-047.523129	**0**	0	**0**	0	**<<****	<<**	**0**	0.0
317	MSH2-intr07 (	02p21	02-047.522602	**0**	0	**0**	0	**<<****	<<**	**0**	0.0
481	EPCAM-9	02p21	02-047.467274	1616	14568	*0.95*	0.03	=	=	61	0.0
472	EPCAM-9	02p21	02-047.467390	1527	13150	*1*	0.04	=	=	52	0.0
217	MSH2-1	02p21	02-047.483423	2228	14629	*0.97*	0.04	=	=	44	0.0
148	MSH2-1	02p21	02-047.483612	2481	15873	*0.99*	0.05	=	=	35	0.0
190	MSH2-1	02p21	02-047.484274	2294	15134	*0.99*	0.03	=	=	47	0.0
160	MSH2-2	02p21	02-047.489105	1818	11895	*0.97*	0.03	=	=	36	-0.1
172	MSH2-3	02p21	02-047.490794	999	6539	***0.51***	0.01	<<*	?	38	0.0
184	MSH2-4	02p21	02-047.493125	952	5937	***0.52***	0.01	<<*	?	43	0.0
211	MSH2-5	02p21	02-047.494910	1179	7857	***0.49***	0.01	<<*	?	50	0.0
231	MSH2-6	02p21	02-047.496965	1291	8734	***0.53***	0.02	<<*	?	47	0.0
249	MSH2-7	02p21	02-047.510479	1913	12576	*0.98*	0.03	=	=	46	0.1
269	MSH2-8	02p21	02-047.526209	2652	19023	*1.04*	0.03	=	=	64	0.0
292	MSH2-9	02p21	02-047.543669	2116	14461	*0.99*	0.03	=	=	45	0.1
310	MSH2-10	02p21	02-047.547615	2238	15511	*1*	0.03	=	=	56	0.0
332	MSH2-11	02p21	02-047.551611	2238	16134	*0.99*	0.03	=	=	48	-0.1
346	MSH2-12	02p21	02-047.555737	2209	17239	*1*	0.03	=	=	54	0.0
364	MSH2-13	02p21	02-047.557048	1937	14598	*1*	0.03	=	=	53	0.0
391	MSH2-14	02p21	02-047.558953	1862	14635	*1.01*	0.02	=	=	60	0.0
409	MSH2-15	02p21	02-047.561413	1823	15035	*1*	0.03	=	=	47	0.0
427	MSH2-16	02p21	02-047.563398	1822	14692	*0.99*	0.03	=	=	48	0.1
142	MLH1-1	03p22.2	03-037.010019	2379	15342	*0.99*	0.04	=	=	42	0.0
154	MLH1-2	03p22.2	03-037.013155	2150	14003	*0.98*	0.02	=	=	38	0.0
166	MLH1-3	03p22.2	03-037.017477	2418	15614	*0.98*	0.03	=	=	53	0.0
178	MLH1-4	03p22.2	03-037.020894	2142	13233	*0.99*	0.03	=	=	39	0.0
203	MLH1-5	03p22.2	03-037.023506	2109	14118	*1.02*	0.02	=	=	44	0.0
224	MLH1-6	03p22.2	03-037.025297	2464	16596	*1.01*	0.03	=	=	44	0.0
242	MLH1-7	03p22.2	03-037.028310	2138	13821	*1.03*	0.03	=	=	49	0.0
256	MLH1-8	03p22.2	03-037.028508	2248	14827	*1.02*	0.03	=	=	56	0.0
278	MLH1-9	03p22.2	03-037.030942	2448	17469	*1*	0.04	=	=	52	0.0
301	MLH1-10	03p22.2	03-037.034002	1957	13967	*0.98*	0.02	=	=	56	-0.1
326	MLH1-11	03p22.2	03-037.036886	2178	15807	*0.95*	0.03	=	=	68	0.0
340	MLH1-12	03p22.2	03-037.042354	2313	17097	*1.03*	0.04	=	=	60	-0.1
355	MLH1-13	03p22.2	03-037.045306	1976	14969	*0.99*	0.03	=	=	53	0.0
382	MLH1-14	03p22.2	03-037.056694	2098	16971	*1.03*	0.04	=	=	61	0.0
401	MLH1-15	03p22.2	03-037.058759	1624	12800	*0.95*	0.03	=	=	43	0.0
418	MLH1-16	03p22.2	03-037.064022	2158	17419	*1*	0.02	=	=	55	0.0
436	MLH1-17	03p22.2	03-037.065060	1830	14706	*0.99*	0.02	=	=	52	0.0
445	MLH1-18	03p22.2	03-037.065438	1747	14352	*1*	0.03	=	=	55	0.1
454	MLH1-19	03p22.2	03-037.067228	1698	14611	*1*	0.02	=	=	59	0.1
287	ARID1A*	01p36.11	01-026.972646	2389	16612	*0.99*	0.04	=	=	42	0.1
236	SCN1A*	02q24.3	02-166.713867	2044	14008	*1*	0.03	=	=	63	0.0
375	CASP6*	04q25	04-110.831524	2284	17246	*1.01*	0.02	=	=	46	0.0
130	IL4*	05q31.1	05-132.037609	2448	15602	*1*	0.04	=	=	58	0.0
196	SLC26A3*	07q31.1	07-107.195395	1858	12053	*1.01*	0.02	=	=	46	0.0
463	UPF2*	10p14	10-012.018627	1926	16011	*0.98*	0.02	=	=	47	0.1
136	CREM*	10p11.21	10-035.517226	2117	13762	*1.01*	0.03	=	=	39	0.0
490	LATS2*	13q12.11	13-020.447119	1518	12848	*1.01*	0.02	=	=	43	0.0
499	RPGRIP1*	14q11.2	14-020.859957	1557	13880	*0.99*	0.03	=	=	48	0.0
**Median value all probe values:**				**2104**	**14632**	**0.99**	**0.03**			**48**	**0**

Figure (xxi): MLPA results showing (MSH6:c.(?_-21)_(*21_?)del

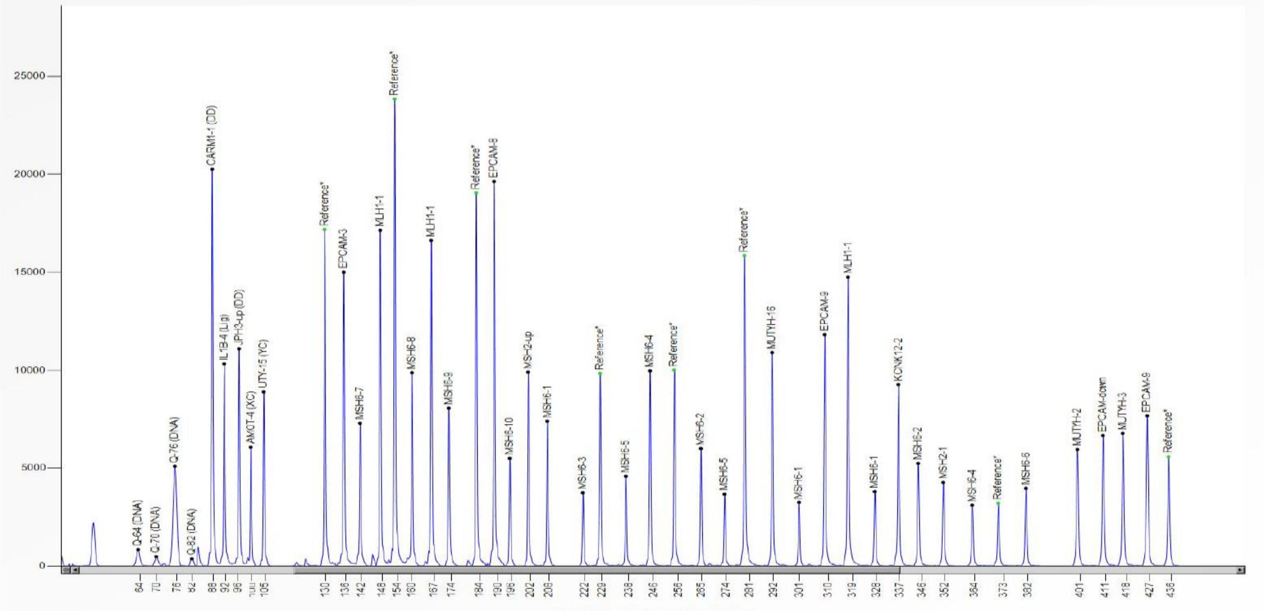

D [nt]	Gene-Exon	Chr.band	hg18 loc.	Height	Area	Ratio	Stdev	[REF]	[Sam]	Width	d[nt]
292	MUTYH-16	01p34.1	01-045.567618	10917	90173	*0.99*	0.05	=	=	58	0.0
418	MUTYH-3	01p34.1	01-045.571725	6771	69924	*1.06*	0.05	=	=	72	0.0
401	MUTYH-2	01p34.1	01-045.572632	5952	59318	*1.03*	0.07	=	=	65	0.0
136	EPCAM-3	02p21	02-047.454615	15016	114458	*0.91*	0.04	=	=	65	-0.1
190	EPCAM-8	02p21	02-047.465789	19644	150503	*0.93*	0.04	=	=	63	0.1
310	EPCAM-9	02p21	02-047.467274	11832	99506	*1.02*	0.04	=	=	79	0.0
427	EPCAM-9	02p21	02-047.467390	7693	78956	*1.08*	0.07	=	=	64	0.0
411	EPCAM-down	02p21	02-047.470732	6657	66643	*1.02*	0.06	=	=	61	0.1
202	MSH2-up	02p21	02-047.481005	9911	70736	***0.52***	0.02	<<*	?	55	0.0
352	MSH2-1	02p21	02-047.483612	4263	39404	***0.51***	0.03	<<*	?	54	0.0
337	KCNK12-2	02p21	02-047.601937	9289	80530	***0.58***	0.03	<<*	?	63	0.0
301	MSH6-1	02p16.3	02-047.863538	3246	26490	***0.54***	0.05	<<*	?	45	0.0
208	MSH6-1	02p16.3	02-047.863722	7410	54301	***0.52***	0.02	<<*	?	47	0.0
328	MSH6-1	02p16.3	02-047.863863	3808	33416	***0.52***	0.02	<<*	?	52	0.0
265	MSH6-2	02p16.3	02-047.871587	5991	44963	***0.54***	0.03	<<*	?	40	0.0
346	MSH6-2	02p16.3	02-047.871666	5241	48801	***0.53***	0.03	<<*	?	70	0.0
222	MSH6-3	02p16.3	02-047.876646	3737	27278	***0.51***	0.02	<<*	?	48	0.1
364	MSH6-4	02p16.3	02-047.879491	3105	29371	***0.55***	0.05	<<*	?	62	0.0
246	MSH6-4	02p16.3	02-047.881068	9984	77845	***0.52***	0.02	<<*	?	61	0.0
238	MSH6-5	02p16.3	02-047.884071	4581	34982	***0.52***	0.03	<<*	?	57	0.0
274	MSH6-5	02p16.3	02-047.884239	3671	28746	***0.49***	0.02	<<*	?	51	0.0
382	MSH6-6	02p16.3	02-047.885564	3971	37306	***0.54***	0.02	<<*	?	58	-0.1
142	MSH6-7	02p16.3	02-047.886280	7313	57882	***0.48***	0.02	<<*	?	60	-0.1
160	MSH6-8	02p16.3	02-047.886932	9883	78938	***0.49***	0.02	<<*	?	61	0.0
174	MSH6-9	02p16.3	02-047.887160	8075	62198	***0.48***	0.02	<<*	?	53	0.1
196	MSH6-10	02p16.3	02-047.887468	5492	40226	***0.49***	0.02	<<*	?	37	-0.1
319	MLH1-1	03p22.2	03-037.009357	14760	131808	*0.97*	0.05	=	=	78	0.0
148	MLH1-1	03p22.2	03-037.009502	17148	121281	*0.92*	0.04	=	=	40	-0.1
167	MLH1-1	03p22.2	03-037.010000	16629	125127	*0.92*	0.04	=	=	53	0.1
256	Reference*	01p13.3	01-107.492319	10020	79559	*1.01*	0.04	=	=	67	0.0
154	Reference*	08q22.2	08-100.184502	23851	180841	*0.91*	0.04	=	=	53	0.0
130	Reference*	11q13.1	11-065.489151	17185	123004	*0.93*	0.04	=	=	43	-0.1
281	Reference*	11q22.3	11-109.613470	15853	134907	*1*	0.04	=	=	91	0.1
229	Reference*	11q24.2	11-125.008325	9853	77304	*0.99*	0.04	=	=	78	0.1
373	Reference*	20p13	20-003.847397	3201	31673	*1.06*	0.04	=	=	83	0.0
436	Reference*	20p12.2	20-010.572998	5575	56969	*1.03*	0.04	=	=	62	0.0
184	Reference*	21q22.11	21-034.658244	19063	137969	*0.98*	0.05	=	=	56	0.0
**Median value all probe values:**				**7693**	**69924**	**0.91**	**0.04**			**60**	**0.01**

Figure (xxii): MLPA results showing MSH6: c.(?_-21)_(260+21_261-21)del

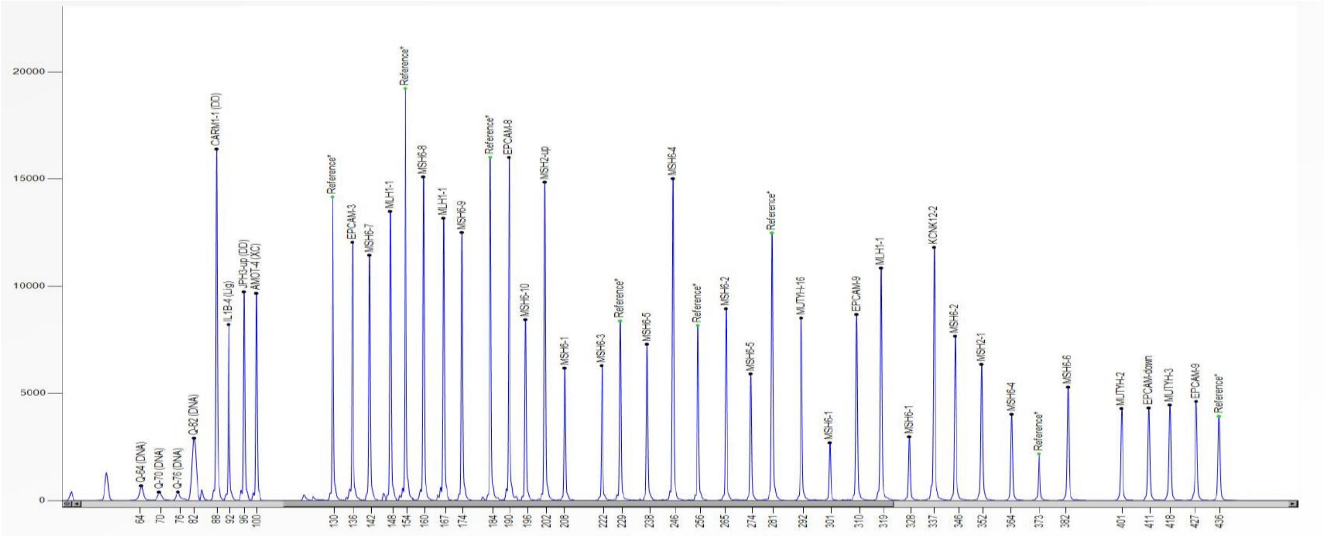

D [nt]	Gene-Exon	Chr.band	hg18 loc.	Height	Area	Ratio[H]	Stdev	[REF]	[Sam]	Width	d[nt]
292	MUTYH-16	01p34.1	01-045.567618	8517	62258	*0.9*	0.06	=	=	66	0.0
418	MUTYH-3	01p34.1	01-045.571725	4456	40848	*0.88*	0.06	=	=	50	0.1
401	MUTYH-2	01p34.1	01-045.572632	4291	38101	*0.88*	0.07	=	=	52	0.1
136	EPCAM-3	02p21	02-047.454615	12052	82832	*1.01*	0.06	=	=	59	0.0
190	EPCAM-8	02p21	02-047.465789	16010	107590	*1*	0.06	=	=	37	-0.1
310	EPCAM-9	02p21	02-047.467274	8682	64465	*0.99*	0.05	=	=	69	0.0
427	EPCAM-9	02p21	02-047.467390	4621	43469	*0.89*	0.05	=	=	68	0.1
411	EPCAM-down	02p21	02-047.470732	4315	38175	*0.93*	0.05	=	=	52	0.0
202	MSH2-up	02p21	02-047.481005	14859	97530	*0.99*	0.05	=	=	72	-0.1
352	MSH2-1	02p21	02-047.483612	6356	53393	*0.92*	0.04	=	=	73	0.1
337	KCNK12-2	02p21	02-047.601937	11813	96154	*0.96*	0.05	=	=	83	-0.1
301	MSH6-1	02p16.3	02-047.863538	2693	19580	***0.49***	0.03	<<*	=	46	0.1
208	MSH6-1	02p16.3	02-047.863722	6178	40860	***0.54***	0.03	<<*	=	52	0.0
328	MSH6-1	02p16.3	02-047.863863	2972	23496	***0.49***	0.03	<<*	=	64	0.0
265	MSH6-2	02p16.3	02-047.871587	8949	60910	*0.96*	0.05	=	=	54	0.0
346	MSH6-2	02p16.3	02-047.871666	7669	61038	*0.91*	0.06	=	=	71	0.0
222	MSH6-3	02p16.3	02-047.876646	6293	42196	*1.02*	0.05	=	=	61	0.1
364	MSH6-4	02p16.3	02-047.879491	4024	32688	*0.89*	0.06	=	=	54	-0.1
246	MSH6-4	02p16.3	02-047.881068	15021	102169	*1.01*	0.06	=	=	68	0.0
238	MSH6-5	02p16.3	02-047.884071	7295	50107	*0.93*	0.05	=	=	52	0.0
274	MSH6-5	02p16.3	02-047.884239	5910	41415	*0.97*	0.06	=	=	53	0.1
382	MSH6-6	02p16.3	02-047.885564	5290	44703	*0.88*	0.05	=	=	61	-0.1
142	MSH6-7	02p16.3	02-047.886280	11448	75022	*1.02*	0.05	=	=	48	0.0
160	MSH6-8	02p16.3	02-047.886932	15103	100455	*1.01*	0.06	=	=	60	0.0
174	MSH6-9	02p16.3	02-047.887160	12508	81816	*1.02*	0.06	=	=	64	-0.1
196	MSH6-10	02p16.3	02-047.887468	8441	55215	*1*	0.06	=	=	52	-0.1
319	MLH1-1	03p22.2	03-037.009357	10850	82832	*0.96*	0.06	=	=	67	0.0
148	MLH1-1	03p22.2	03-037.009502	13494	86898	*0.99*	0.06	=	=	42	0.1
167	MLH1-1	03p22.2	03-037.010000	13176	88308	*0.99*	0.05	=	=	46	0.0
256	Reference*	01p13.3	01-107.492319	8170	56227	*0.99*	0.05	=	=	52	0.0
154	Reference*	08q22.2	08-100.184502	19234	129066	*1.04*	0.06	=	=	62	-0.1
130	Reference*	11q13.1	11-065.489151	14173	91902	*1.06*	0.07	=	=	53	0.1
281	Reference*	11q22.3	11-109.613470	12489	91368	*1*	0.05	=	=	82	0.1
229	Reference*	11q24.2	11-125.008325	8380	57309	*1*	0.05	=	=	68	0.0
373	Reference*	20p13	20-003.847397	2173	18527	*0.88*	0.07	=	=	54	0.0
436	Reference*	20p12.2	20-010.572998	3931	36274	*0.89*	0.08	=	=	58	0.0
184	Reference*	21q22.11	21-034.658244	16016	102396	*1.02*	0.06	=	=	54	0.0
**Median value all probe values:**				**8441**	**60910**	**0.97**	**0.06**			**58**	**0.01**

Figure (xxiii) : MLPA results showing PALB2:c.(2586+21_2587-21)_(2748+21_2749-21)del

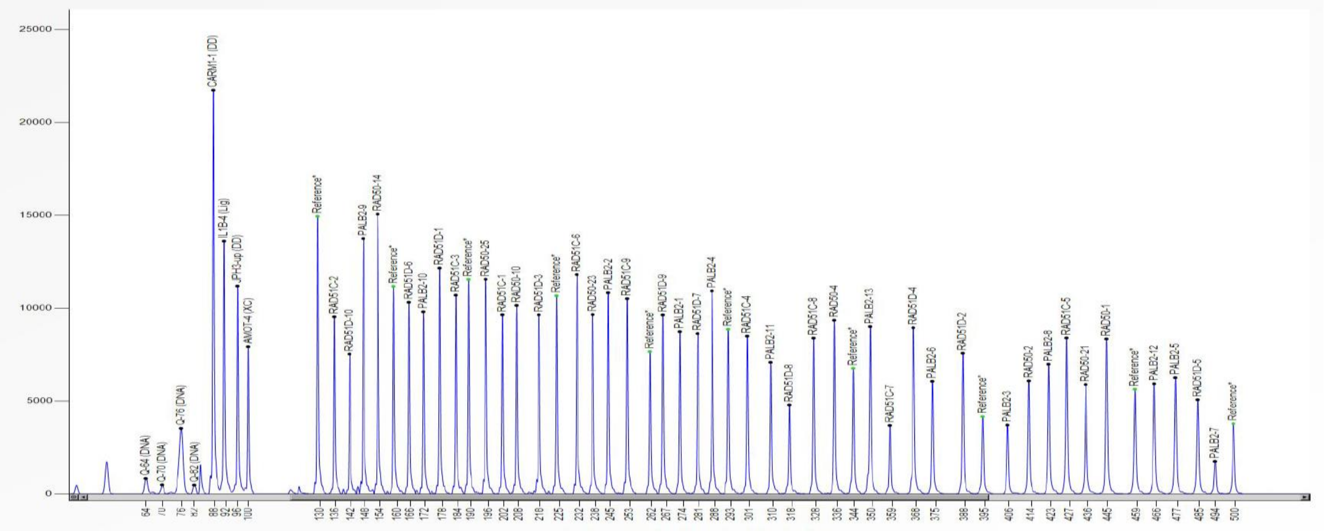

D [nt]	Gene-Exon	Chr.band	hg18 loc.	Height	Area	Ratio [H]	Stdev	[REF]	[Sam]	Width	d[nt]
445	RAD50-1	05q31.1	05-131.920939	8339	86072	*0.97*	0.09	=	=	76	0.1
414	RAD50-2	05q31.1	05-131.922869	6079	61441	*0.97*	0.07	=	=	75	-0.1
336	RAD50-4	05q31.1	05-131.942932	9343	82096	*0.98*	0.08	=	=	66	0.0
208	RAD50-10	05q31.1	05-131.954818	10143	78389	*1.05*	0.06	=	=	62	0.0
154	RAD50-14	05q31.1	05-131.967010	15060	112767	*1.02*	0.08	=	=	48	0.0
436	RAD50-21	05q31.1	05-131.981774	5883	61493	*0.98*	0.05	=	=	74	0.1
238	RAD50-23	05q31.1	05-132.001760	9649	77338	*1.03*	0.06	=	=	57	0.0
196	RAD50-25	05q31.1	05-132.005889	11549	90308	*0.98*	0.06	=	=	54	0.0
350	PALB2-13	16p12.2	16-023.522409	9004	89363	*0.98*	0.07	=	=	68	0.0
466	PALB2-12	16p12.2	16-023.526633	5921	64688	*0.98*	0.06	=	=	75	0.0
310	PALB2-11	16p12.2	16-023.532832	7079	60745	*1.09*	**0.11**	=	=	64	0.0
172	PALB2-10	16p12.2	16-023.540210	9798	73447	*1.06*	0.1	=	=	45	0.0
148	PALB2-9	16p12.2	16-023.541776	13741	104954	*0.95*	0.05	=	=	43	0.0
423	PALB2-8	16p12.2	16-023.542867	6979	70926	*0.92*	0.06	=	=	66	0.1
494	PALB2-7	16p12.2	16-023.545151	1746	19592	***0.46***	0.03	**<<***	<*	48	0.1
375	PALB2-6	16p12.2	16-023.548027	6055	57763	*0.94*	0.06	=	=	70	0.1
477	PALB2-5	16p12.2	16-023.549183	6254	68051	*0.99*	0.07	=	=	80	0.1
288	PALB2-4	16p12.2	16-023.554181	10923	93559	*0.95*	0.06	=	=	60	0.1
406	PALB2-3	16p12.2	16-023.556620	3702	36828	*0.98*	0.09	=	=	56	0.1
245	PALB2-2	16p12.2	16-023.556874	10830	86082	*1.02*	0.06	=	=	54	0.0
274	PALB2-1	16p12.2	16-023.559963	8731	70424	*1.12*	**0.11**	=	=	68	-0.1
142	RAD51D-10	17q12	17-030.452059	7525	54685	*1.07*	**0.15**	=	=	42	0.0
267	RAD51D-9	17q12	17-030.452394	9632	82388	*0.93*	0.07	=	=	62	0.0
318	RAD51D-8	17q12	17-030.454373	4782	45110	*1.09*	0.08	=	=	75	-0.1
281	RAD51D-7	17q12	17-030.454635	8627	74257	*0.95*	0.06	=	=	57	0.0
166	RAD51D-6	17q12	17-030.457527	10312	76866	*0.98*	0.07	=	=	42	0.0
485	RAD51D-5	17q12	17-030.458217	5064	55604	*0.91*	0.08	=	=	74	0.1
368	RAD51D-4	17q12	17-030.458511	8941	89041	*0.98*	0.06	=	=	81	0.1
218	RAD51D-3	17q12	17-030.469551	9636	80245	*1.05*	0.08	=	=	58	0.1
388	RAD51D-2	17q12	17-030.470218	7571	72675	*0.97*	0.06	=	=	73	0.1
178	RAD51D-1	17q12	17-030.470829	12151	94444	*1.03*	0.07	=	=	61	0.0
202	RAD51C-1	17q22	17-054.125073	9642	74670	*1.09*	0.08	=	=	52	0.0
136	RAD51C-2	17q22	17-054.127407	9531	70427	*1.09*	**0.13**	=	=	52	-0.1
184	RAD51C-3	17q22	17-054.129144	10698	81416	*1.08*	0.08	=	=	53	0.0
301	RAD51C-4	17q22	17-054.135618	8491	72857	*1.04*	0.06	=	=	73	0.0
427	RAD51C-5	17q22	17-054.142305	8393	84404	*1.03*	0.06	=	=	70	0.0
232	RAD51C-6	17q22	17-054.153266	11804	94406	*0.97*	0.06	=	=	60	0.0
359	RAD51C-7	17q22	17-054.156418	3682	33555	*1.04*	**0.14**	=	=	63	0.0
328	RAD51C-8	17q22	17-054.164845	8384	71272	*1.04*	0.07	=	=	57	0.0
253	RAD51C-9	17q22	17-054.166499	10508	86472	*0.95*	0.06	=	=	57	0.0
500	Reference*	02p13.2	02-071.750350	3778	42512	*0.88*	0.05	=	=	63	0.1
344	Reference*	02q12.3	02-108.971872	6756	63172	*1.02*	0.06	=	=	62	0.0
130	Reference*	04p13	04-042.278439	14942	111488	*1.02*	0.06	=	=	55	-0.1
395	Reference*	06p12.2	06-052.015764	4161	43193	*1.09*	**0.11**	=	=	70	0.1
459	Reference*	07p14.3	07-030.637652	5635	60655	*1.01*	0.06	=	=	74	0.0
190	Reference*	09p13.3	09-034.483216	11543	89671	*0.96*	0.06	=	=	53	0.0
225	Reference*	09q34.13	09-134.207927	10666	83485	*1.05*	0.06	=	=	58	0.0
293	Reference*	12q12	12-038.037306	8858	78270	*0.94*	0.06	=	=	61	0.0
160	Reference*	15q21.1	15-042.674827	11168	85522	*0.98*	0.06	=	=	62	0.0
262	Reference*	18q21.1	18-045.743292	7659	62896	*1.01*	0.1	=	=	51	0.0
**Median value all probe values:**				**8794**	**74464**	**0.99**	**0.06**			**61.5**	**0.01**

Figure (xxiv): MLPA results showing PMS2:c.(2006+21_2007-21)_(*21_?)dup

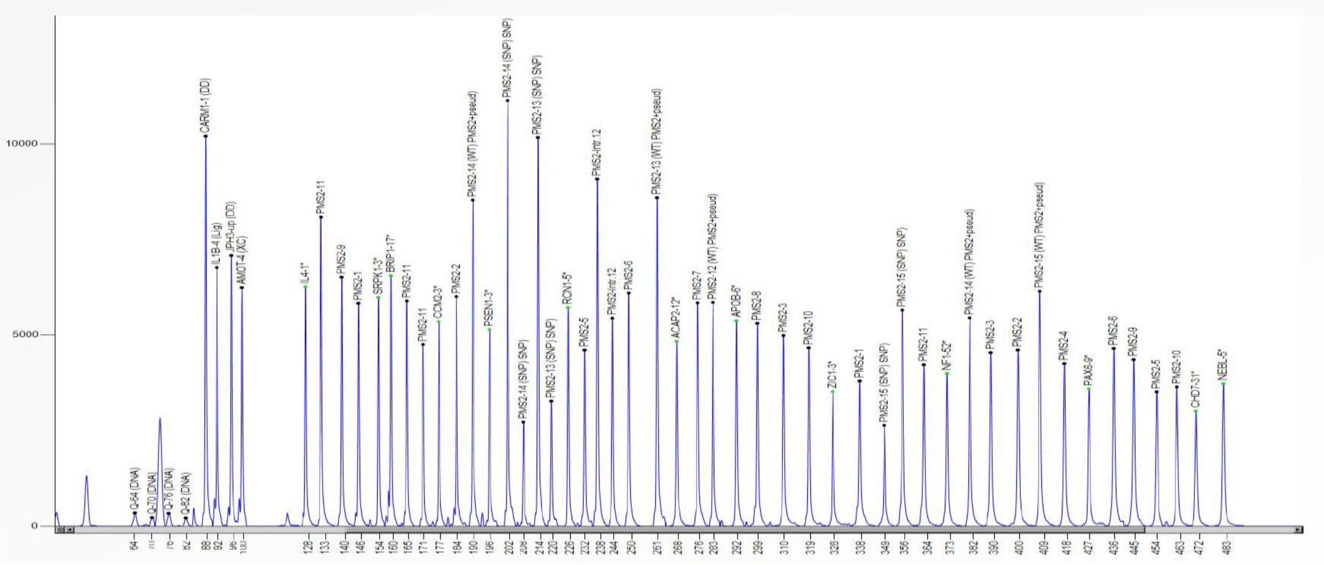

D [nt]	Gene-Exon	Chr.band	hg18 loc.	Height	Area	Ratio [H]	Stdev	[REF]	[Sam]	Width	d[nt]
356	PMS2-15 (SNP)	07p22.1	07-005.979414	5654	67545	1.41	0.06	>>*	?	83	0.1
349	PMS2-15 (SNP)	07p22.1	07-005.979414	2639	31966	0.96	0.04	=	=	76	0.0
409	PMS2-15 (WT)	07p22.1	07-005.979589	6147	79176	1.19	0.04	>>	=	97	0.1
190	PMS2-14 (WT)	07p22.1	07-005.983637	8533	75914	1.22	0.04	>>	=	55	0.0
382	PMS2-14 (WT)	07p22.1	07-005.983713	5449	66571	1.24	0.03	>>	=	87	0.1
208	PMS2-14 (SNP)	07p22.1	07-005.983818	2724	24414	0.48	0.01	<<*	?	40	0.0
202	PMS2-14 (SNP)	07p22.1	07-005.983818	11136	100048	2	0.09	>>*	>*	58	0.0
220	PMS2-13 (SNP)	07p22.1	07-005.984734	3273	30500	0.52	0.02	<<*	?	50	0.0
214	PMS2-13 (SNP)	07p22.1	07-005.984734	10169	91672	1.97	0.06	>>*	>*	64	0.1
261	PMS2-13 (WT)	07p22.1	07-005.984837	8594	82067	1.25	0.04	>>	=	47	0.1
244	PMS2-Intr.12	07p22.1	07-005.987848	5440	49980	0.92	0.03	=	=	53	0.1
238	PMS2-Intr.12	07p22.1	07-005.987848	9083	86211	1.46	0.05	>>*	>*	69	0.1
283	PMS2-12 (WT)	07p22.1	07-005.989043	5855	58913	1.24	0.03	>>	=	51	0.0
364	PMS2-11	07p22.1	07-005.992998	4225	52237	0.99	0.04	=	=	97	0.1
171	PMS2-11	07p22.1	07-005.993438	4754	40344	1.06	0.04	=	=	50	0.0
165	PMS2-11	07p22.1	07-005.993438	5894	51312	1.02	0.04	=	=	54	0.0
133	PMS2-11	07p22.1	07-005.993521	8087	72718	0.92	0.04	=	=	80	-0.1
319	PMS2-10	07p22.1	07-005.995963	4666	49783	0.98	0.03	=	=	67	0.0
463	PMS2-10	07p22.1	07-005.996040	3643	47961	0.96	0.04	=	=	83	0.0
140	PMS2-9	07p22.1	07-005.998150	6516	57472	1	0.03	=	=	77	0.0
445	PMS2-9	07p22.1	07-005.998212	4357	60785	1	0.03	=	=	103	0.1
299	PMS2-8	07p22.1	07-006.001701	5308	57645	0.99	0.02	=	=	78	0.0
276	PMS2-7	07p22.1	07-006.003491	5842	60975	0.93	0.03	=	=	75	0.1
250	PMS2-6	07p22.1	07-006.005302	6101	59855	0.98	0.03	=	=	76	0.1
436	PMS2-6	07p22.1	07-006.005361	4649	63358	1.04	0.03	=	=	63	0.1
454	PMS2-5	07p22.1	07-006.008620	3516	45529	1.01	0.02	=	=	70	-0.1
232	PMS2-5	07p22.1	07-006.008702	4608	42826	0.99	0.05	=	=	41	0.0
418	PMS2-4	07p22.1	07-006.009732	4254	55230	0.96	0.03	=	=	91	0.1
310	PMS2-3	07p22.1	07-006.010009	4989	54204	1.07	0.03	=	=	73	0.0
390	PMS2-3	07p22.1	07-006.010308	4542	58003	1.03	0.04	=	=	95	0.1
184	PMS2-2	07p22.1	07-006.012054	6008	54450	0.98	0.03	=	=	51	0.0
400	PMS2-2	07p22.1	07-006.012144	4609	63002	0.95	0.07	=	=	101	0.2
146	PMS2-1	07p22.1	07-006.015204	5832	52612	0.98	0.05	=	=	65	0.1
338	PMS2-1	07p22.1	07-006.015443	3799	48212	1.03	0.02	=	=	77	-0.1
292	APOB-6*	02p24.1	02-021.113531	5375	53707	1	0.02	=	=	65	0.1
328	ZIC1-3*	03q24	03-148.613909	3523	37782	1.02	0.04	=	=	70	0.0
268	ACAP2-12*	03q29	03-196.509258	4835	50033	0.97	0.03	=	=	80	0.1
128	IL4-1*	05q31.1	05-132.037610	6264	53717	1.01	0.04	=	=	73	0.0
154	SRPK1-3*	06p21.31	06-035.966706	5988	48811	1.02	0.03	=	=	43	0.0
177	CCM2-3*	07p13	07-045.044376	5350	45466	1.01	0.02	=	=	50	0.1
483	NEBL-5*	10p12.31	10-021.226273	3728	51317	1.01	0.03	=	=	90	0.0
427	PAX6-9*	11p13	11-031.772203	3591	45613	0.97	0.02	=	=	79	0.0
226	RCN1-5*	11p13	11-032.081884	5717	50965	0.99	0.02	=	=	50	0.0
196	PSEN1-3*	14q24.2	14-072.684492	5144	44747	0.95	0.04	=	=	42	0.0
373	NF1-52*	17q11.2	17-026.707635	3994	48549	1.02	0.02	=	=	92	0.0
160	BRIP1-17*	17q23.2	17-057.148115	6554	61901	0.99	0.03	=	=	59	0.0
472	CHD7-31*	08q12.2	08-061.928317	3011	39949	1	0.05	=	=	79	0.0
**Median value all probe values:**				**5308**	**53707**	**1**	**0.03**			**70**	**0.02**

Figure (xxv): MLPA results showing PMS2:c.(2445+21_2446-21)_(*21_?)del

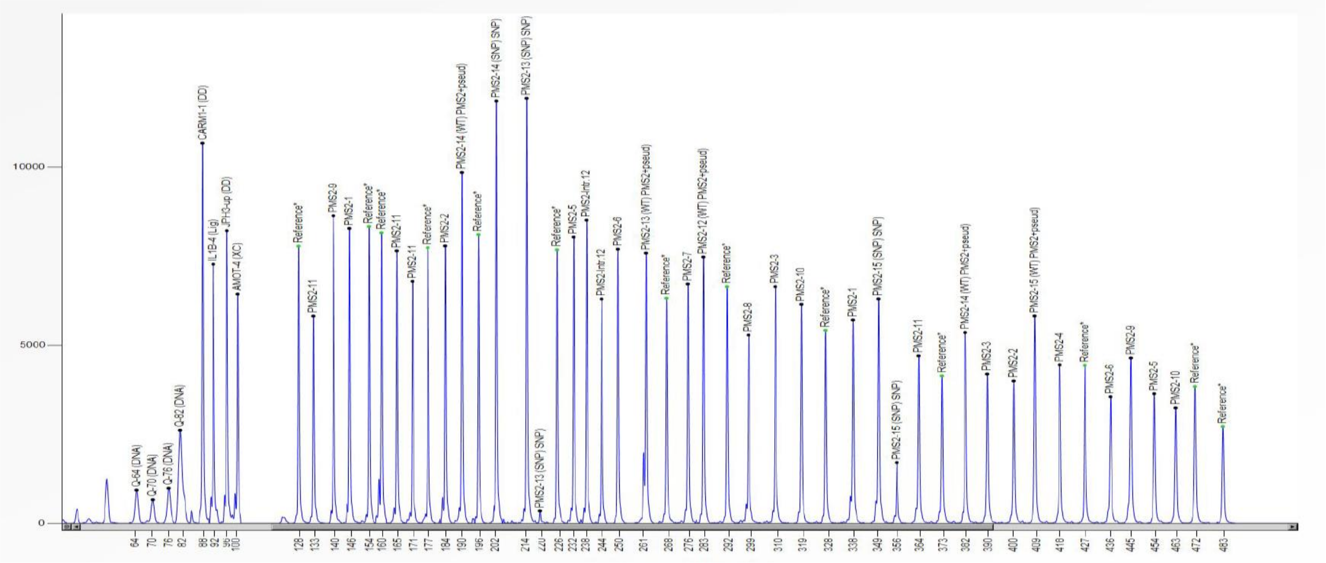

D [nt]	Gene-Exon	Chr.band	hg18 loc.	Height	Area	Ratio^{H}	Stdev	[REF]	[Sam]	Width	d[nt]
356	PMS2-15 (SNP)	07p22.1	07-005.979414	1704	16328	*0.61*	0.06	<<*	=	51	0.0
349	PMS2-15 (SNP)	07p22.1	07-005.979414	6297	58125	*1.55*	**0.14**	>>*	=	63	0.0
409	PMS2-15 (WT)	07p22.1	07-005.979589	5819	54644	*1.06*	0.1	=	=	75	0.1
190	PMS2-14 (WT)	07p22.1	07-005.983637	9847	72375	*0.94*	0.08	=	=	72	0.0
382	PMS2-14 (WT)	07p22.1	07-005.983713	5354	50048	*0.9*	0.09	=	=	64	0.1
208	PMS2-14 (SNP)	07p22.1	07-005.983818	**0**	0	**0**	0	<<**	<<**	**0**	0.0
202	PMS2-14 (SNP)	07p22.1	07-005.983818	11852	86204	*2.4*	**0.25**	>>*	=	59	0.0
220	PMS2-13 (SNP)	07p22.1	07-005.984734	349	2803	*0.05*	0	<<*	=	28	0.0
214	PMS2-13 (SNP)	07p22.1	07-005.984734	11928	87428	*2.02*	**0.18**	>>*	=	68	0.0
261	PMS2-13 (WT)	07p22.1	07-005.984837	7584	72619	*0.96*	0.09	=	=	84	0.0
244	PMS2-Intr.12	07p22.1	07-005.987848	6295	47815	*0.98*	**0.11**	=	=	45	-0.1
238	PMS2-Intr.12	07p22.1	07-005.987848	8509	65070	*1.01*	0.09	=	=	60	0.0
283	PMS2-12 (WT)	07p22.1	07-005.989043	7473	59499	*1.02*	0.09	=	=	47	0.0
364	PMS2-11	07p22.1	07-005.992998	4700	44781	*0.91*	0.08	=	=	76	0.0
171	PMS2-11	07p22.1	07-005.993438	6791	50323	*0.96*	0.09	=	=	66	0.0
165	PMS2-11	07p22.1	07-005.993438	7647	56407	*0.98*	0.09	=	=	66	0.0
133	PMS2-11	07p22.1	07-005.993521	5816	41573	*1.05*	**0.14**	=	=	52	-0.1
319	PMS2-10	07p22.1	07-005.995963	6146	51416	*0.98*	0.1	=	=	79	-0.1
463	PMS2-10	07p22.1	07-005.996040	3240	33292	*0.88*	0.1	=	=	74	0.0
140	PMS2-9	07p22.1	07-005.998150	8632	62269	*1.07*	**0.12**	=	=	57	-0.1
445	PMS2-9	07p22.1	07-005.998212	4640	49183	*1*	0.1	=	=	64	0.0
299	PMS2-8	07p22.1	07-006.001701	5284	46317	*1.1*	0.1	=	=	62	-0.1
276	PMS2-7	07p22.1	07-006.003491	6716	53879	*0.94*	0.08	=	=	47	0.0
250	PMS2-6	07p22.1	07-006.005302	7694	57796	*0.95*	0.08	=	=	56	0.0
436	PMS2-6	07p22.1	07-006.005361	3552	37412	**1.3**	**0.15**	>*	=	84	0.0
454	PMS2-5	07p22.1	07-006.008620	3638	36331	*1.08*	**0.13**	=	=	81	0.0
232	PMS2-5	07p22.1	07-006.008702	8034	61444	*1.04*	0.1	=	=	54	0.0
418	PMS2-4	07p22.1	07-006.009732	4447	43207	*0.99*	**0.11**	=	=	64	0.0
310	PMS2-3	07p22.1	07-006.010009	6641	55393	*1.04*	0.09	=	=	66	-0.1
390	PMS2-3	07p22.1	07-006.010308	4189	40355	*1.02*	0.1	=	=	68	0.0
184	PMS2-2	07p22.1	07-006.012054	7786	59745	*1*	0.09	=	=	46	0.1
400	PMS2-2	07p22.1	07-006.012144	3995	37064	*1*	0.09	=	=	64	0.0
146	PMS2-1	07p22.1	07-006.015204	8277	61361	*0.94*	0.09	=	=	74	0.0
338	PMS2-1	07p22.1	07-006.015443	5706	53997	*1.14*	**0.11**	=	=	65	0.0
292	Reference*	02p24.1	02-021.113531	6642	52516	*1.1*	0.1	=	=	50	0.0
328	Reference*	03q24	03-148.613909	5415	45565	*0.98*	0.1	=	=	66	0.0
268	Reference*	03q29	03-196.509258	6317	50493	*1.09*	0.1	=	=	62	0.0
128	Reference*	05q31.1	05-132.037607	7779	54646	*0.89*	**0.11**	=	=	64	-0.1
154	Reference*	06p21.31	06-035.966706	8331	57590	*0.99*	0.1	=	=	44	0.0
177	Reference*	07p13	07-045.044376	7735	56107	*0.9*	0.09	=	=	60	0.1
483	Reference*	10p12.31	10-021.226273	2715	28008	*0.97*	0.1	=	=	74	0.0
427	Reference*	11p13	11-031.772203	4435	42624	*0.94*	0.1	=	=	73	0.0
226	Reference*	11p13	11-032.081884	7677	55380	*1.17*	**0.13**	=	=	56	-0.1
196	Reference*	14q24.2	14-072.684492	8104	57239	*1.03*	0.1	=	=	44	0.1
373	Reference*	17q11.2	17-026.707635	4137	39695	*1.13*	0.1	=	=	70	0.0
160	Reference*	17q23.2	17-057.148115	8155	**63859**	*0.92*	0.1	=	=	44	0.1
472	Reference*	08q12.2	08-061.928317	3839	39834	*0.97*	**0.11**	=	=	70	0.0
**Median value all probe values:**				**6307**	**53198**	**0.99**	**0.1**			**64**	**-0.01**

Figure (xxvi) : MLPA results showing PMS2:c.(537+21_538-21)_(903+21_904-21)del

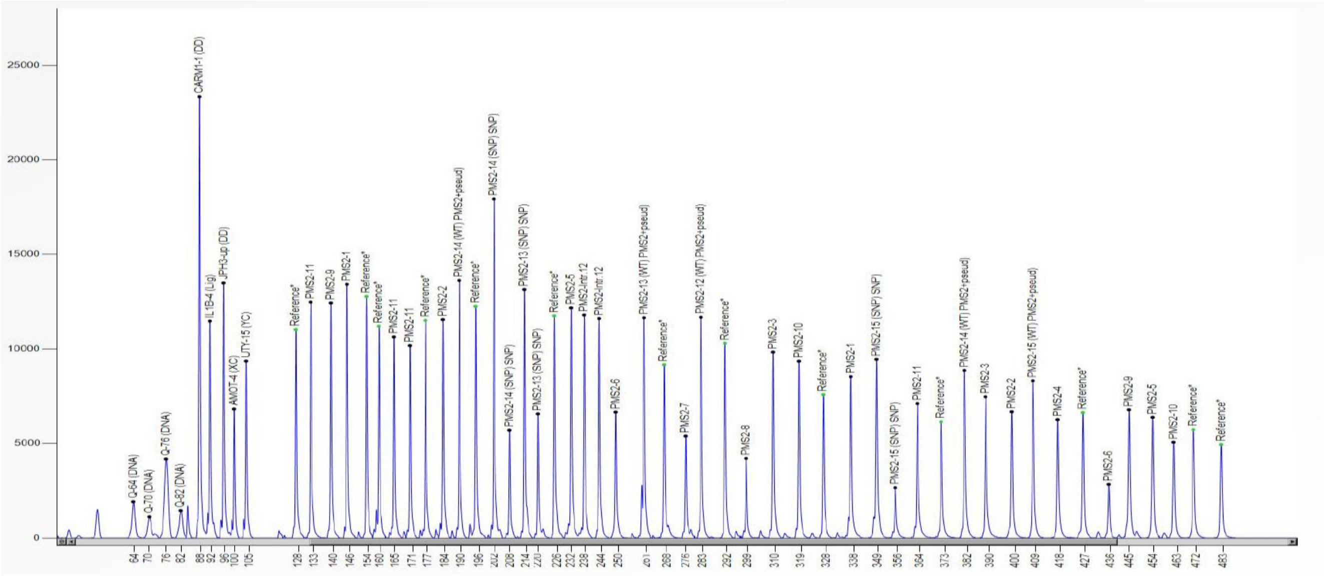

D [nt]	Gene-Exon	Chr.band	hg18 loc.	Height	Area	Ratio	Stdev	[REF]	[Sam]	Width	d[nt]
356	PMS2-15 (SNP)	07p22.1	07-005.979414	2664	34182	***0.5***	0.02	<<*	?	68	-0.1
349	PMS2-15 (SNP)	07p22.1	07-005.979414	9456	111221	***1.44***	0.07	>>*	?	97	0.0
409	PMS2-15 (WT)	07p22.1	07-005.979589	8317	95771	*1*	0.06	=	=	81	0.0
190	PMS2-14 (WT)	07p22.1	07-005.983637	13625	118403	*0.97*	0.04	=	=	54	0.0
382	PMS2-14 (WT)	07p22.1	07-005.983713	8865	102770	*1*	0.05	=	=	81	0.0
208	PMS2-14 (SNP)	07p22.1	07-005.983818	5704	50931	***0.49***	0.02	<<*	?	54	0.0
202	PMS2-14 (SNP)	07p22.1	07-005.983818	17937	153007	***1.49***	0.07	>>*	?	40	0.0
220	PMS2-13 (SNP)	07p22.1	07-005.984734	6569	58587	***0.53***	0.02	<<*	?	38	-0.1
214	PMS2-13 (SNP)	07p22.1	07-005.984734	13145	118303	***1.45***	0.07	>>*	?	59	0.0
261	PMS2-13 (WT)	07p22.1	07-005.984837	11651	**103808**	*1*	0.04	=	=	36	-0.1
244	PMS2-Intr.12	07p22.1	07-005.987848	11611	106028	*1.06*	0.05	=	=	69	0.0
238	PMS2-Intr.12	07p22.1	07-005.987848	11798	111818	*0.96*	0.04	=	=	70	-0.1
283	PMS2-12 (WT)	07p22.1	07-005.989043	11679	111194	*1.06*	0.05	=	=	58	0.0
364	PMS2-11	07p22.1	07-005.992998	7114	87341	*0.98*	0.04	=	=	87	-0.1
171	PMS2-11	07p22.1	07-005.993438	10182	88124	*0.95*	0.05	=	=	55	0.0
165	PMS2-11	07p22.1	07-005.993438	10638	94172	*0.96*	0.04	=	=	73	0.0
133	PMS2-11	07p22.1	07-005.993521	12479	110841	*1.03*	0.06	=	=	67	0.0
319	PMS2-10	07p22.1	07-005.995963	9356	94351	*1.01*	0.04	=	=	64	-0.1
463	PMS2-10	07p22.1	07-005.996040	5068	60571	*0.99*	0.05	=	=	72	0.1
140	PMS2-9	07p22.1	07-005.998150	12434	108519	*0.98*	0.05	=	=	63	0.0
445	PMS2-9	07p22.1	07-005.998212	6788	82683	*0.94*	0.05	=	=	56	0.1
299	PMS2-8	07p22.1	07-006.001701	4214	42078	***0.55***	0.02	<<*	<*	54	0.0
276	PMS2-7	07p22.1	07-006.003491	5396	51801	***0.51***	0.02	<<*	<*	37	-0.1
250	PMS2-6	07p22.1	07-006.005302	6662	68155	***0.55***	0.02	<<*	<*	76	-0.1
436	PMS2-6	07p22.1	07-006.005361	2838	32118	***0.46***	0.02	<<*	<*	57	0.1
454	PMS2-5	07p22.1	07-006.008620	6385	76857	*1.09*	0.06	=	=	74	0.0
232	PMS2-5	07p22.1	07-006.008702	12176	120972	*1.08*	0.05	=	=	64	0.0
418	PMS2-4	07p22.1	07-006.009732	6263	74031	*0.94*	0.04	=	=	89	0.0
310	PMS2-3	07p22.1	07-006.010009	9835	99316	*0.94*	0.05	=	=	63	-0.1
390	PMS2-3	07p22.1	07-006.010308	7471	88598	*0.97*	0.04	=	=	96	0.1
184	PMS2-2	07p22.1	07-006.012054	11555	104109	*1.02*	0.05	=	=	54	0.0
400	PMS2-2	07p22.1	07-006.012144	6681	79579	*0.98*	0.04	=	=	103	0.0
146	PMS2-1	07p22.1	07-006.015204	13425	115886	*1.05*	0.06	=	=	67	0.0
338	PMS2-1	07p22.1	07-006.015443	8537	98442	*1.04*	0.05	=	=	90	0.0
292	Reference*	02p24.1	02-021.113531	10307	96623	*1.15*	**0.17**	=	=	71	-0.1
328	Reference*	03q24	03-148.613909	7591	76117	*0.99*	0.04	=	=	66	0.0
268	Reference*	03q29	03-196.509258	9167	94377	*0.96*	0.04	=	=	89	-0.1
128	Reference*	05q31.1	05-132.037607	11026	96672	*0.97*	0.06	=	=	63	0.0
154	Reference*	06p21.31	06-035.966706	12781	107747	*1.07*	0.05	=	=	46	-0.1
177	Reference*	07p13	07-045.044376	11516	**101251**	*1*	0.04	=	=	58	-0.1
483	Reference*	10p12.31	10-021.226273	4952	61900	*0.93*	0.08	=	=	96	0.0
427	Reference*	11p13	11-031.772203	6638	76738	*1.03*	0.07	=	=	83	0.0
226	Reference*	11p13	11-032.081884	11754	108327	*0.95*	0.05	=	=	64	0.0
196	Reference*	14q24.2	14-072.684492	12255	110723	*1.02*	0.04	=	=	68	0.0
373	Reference*	17q11.2	17-026.707635	6150	71589	*1.03*	0.05	=	=	99	0.0
160	Reference*	17q23.2	17-057.148115	11205	**104266**	*0.95*	0.05	=	=	49	0.0
472	Reference*	08q12.2	08-061.928317	5736	70819	*1.01*	0.06	=	=	92	0.0
**Median value all probe values:**				**9356**	**95771**	**0.99**	**0.05**			**67**	**-0.02**

Figure (xxvii) : MLPA results showing RAD51C:c.572-?_1131+?del

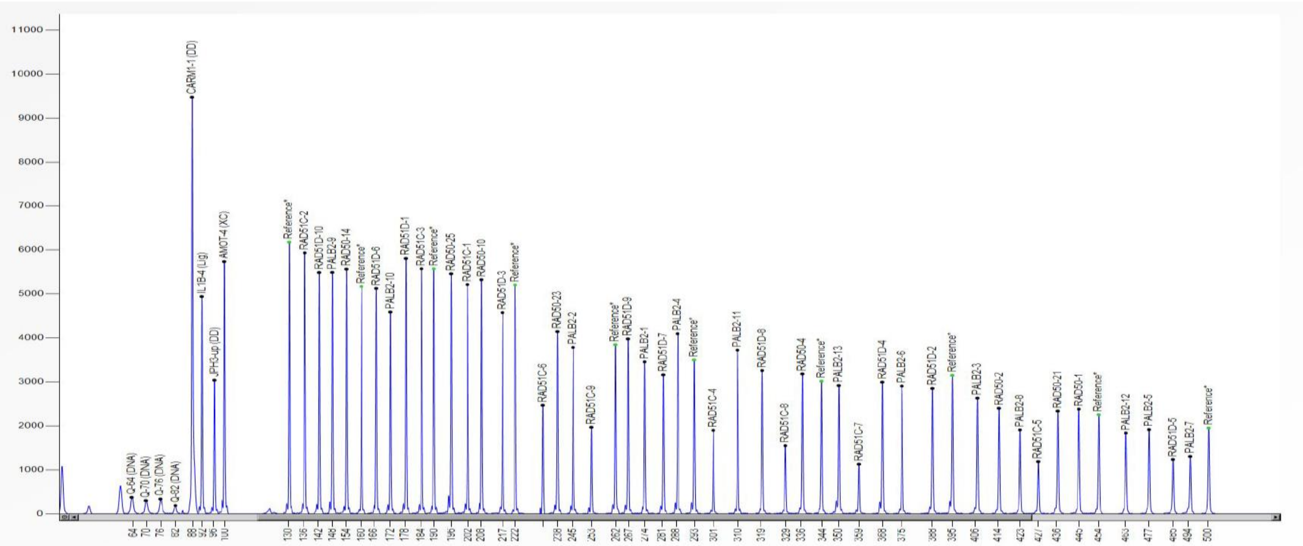

D [nt]	Gene-Exon	Chr.band	hg18 loc.	Height	Area	Ratio^{H}	Stdev	[REF]	[Sam]	Width	d[nt]
445	RAD50-1	05q31.1	05-131.920939	2381	19004	1	0.03	=	=	41	0.0
414	RAD50-2	05q31.1	05-131.922869	2400	18038	1.06	0.03	=	=	59	0.0
336	RAD50-4	05q31.1	05-131.942932	3182	22442	1.05	0.03	=	=	54	0.0
208	RAD50-10	05q31.1	05-131.954818	5324	32123	1	0.03	=	=	59	0.0
154	RAD50-14	05q31.1	05-131.967010	5565	33062	1.04	0.04	=	=	55	0.1
436	RAD50-21	05q31.1	05-131.981774	2336	18929	1.01	0.03	=	=	60	0.0
238	RAD50-23	05q31.1	05-132.001760	4144	26417	0.99	0.02	=	=	41	0.0
196	RAD50-25	05q31.1	05-132.005889	5460	34515	0.99	0.02	=	=	39	0.0
350	PALB2-13	16p12.2	16-023.522409	2916	22359	0.99	0.03	=	=	62	0.1
463	PALB2-12	16p12.1	16-023.526713	1836	15337	1	0.03	=	=	41	0.0
310	PALB2-11	16p12.2	16-023.532832	3722	24577	1.02	0.03	=	=	45	0.0
172	PALB2-10	16p12.2	16-023.540210	4589	27500	0.95	0.03	=	=	52	0.0
148	PALB2-9	16p12.2	16-023.541776	5490	33160	0.96	0.02	=	=	37	0.1
423	PALB2-8	16p12.2	16-023.542867	1907	14840	0.98	0.02	=	=	41	0.0
494	PALB2-7	16p12.2	16-023.545151	1303	11960	1.06	0.03	=	=	63	0.0
375	PALB2-6	16p12.2	16-023.548027	2905	20557	1.02	0.03	=	=	42	0.0
477	PALB2-5	16p12.2	16-023.549183	1914	15707	1.03	0.03	=	=	49	0.0
288	PALB2-4	16p12.2	16-023.554181	4096	27164	1.07	0.03	=	=	60	0.0
406	PALB2-3	16p12.2	16-023.556620	2631	20742	0.97	0.03	=	=	59	-0.1
245	PALB2-2	16p12.2	16-023.556874	3783	23891	0.99	0.03	=	=	51	0.0
274	PALB2-1	16p12.2	16-023.559963	3457	22663	0.98	0.02	=	=	38	0.0
142	RAD51D-10	17q12	17-030.452059	5488	33063	0.99	0.03	=	=	46	0.0
267	RAD51D-9	17q12	17-030.452394	3978	25628	1.03	0.03	=	=	48	0.0
319	RAD51D-8	17q12	17-030.454369	3258	22119	0.99	0.02	=	=	52	0.0
281	RAD51D-7	17q12	17-030.454635	3160	21052	0.98	0.03	=	=	48	0.0
166	RAD51D-6	17q12	17-030.457527	5127	31250	0.99	0.03	=	=	60	-0.1
485	RAD51D-5	17q12	17-030.458217	1234	10630	0.96	0.03	=	=	34	0.0
368	RAD51D-4	17q12	17-030.458511	2994	22597	0.99	0.03	=	=	57	0.0
217	RAD51D-3	17q12	17-030.469675	4575	27673	0.94	0.03	=	=	46	0.0
388	RAD51D-2	17q12	17-030.470218	2851	20836	0.98	0.03	=	=	37	0.0
178	RAD51D-1	17q12	17-030.470829	5808	34567	1	0.03	=	=	52	0.0
202	RAD51C-1	17q22	17-054.125073	5213	32459	0.97	0.03	=	=	44	0.0
136	RAD51C-2	17q22	17-054.127407	5935	35564	0.99	0.03	=	=	48	0.0
184	RAD51C-3	17q22	17-054.129144	5574	33216	0.98	0.03	=	=	52	0.0
301	RAD51C-4	17q22	17-054.135618	1898	12588	**0.53**	0.01	<<*	=	42	0.0
427	RAD51C-5	17q22	17-054.142305	1186	9306	**0.53**	0.01	<<*	=	37	0.0
233	RAD51C-6	17q22	17-054.153121	2469	14243	**0.48**	0.02	<<*	=	**14**	0.0
359	RAD51C-7	17q22	17-054.156418	1128	8483	**0.48**	0.02	<<*	=	44	0.0
329	RAD51C-8	17q22	17-054.164834	1549	11052	**0.5**	0.01	<<*	=	43	0.0
253	RAD51C-9	17q22	17-054.166499	1967	12623	**0.49**	0.01	<<*	=	41	0.0
500	Reference*	02p13.2	02-071.750350	1951	16773	0.96	0.04	=	=	64	-0.4
344	Reference*	02q13	02-108.971872	3017	21468	0.95	0.02	=	=	45	-0.1
222	Reference*	03q21.1	03-123.483712	5204	30788	1.02	0.03	=	=	42	0.0
130	Reference*	04p13	04-042.278439	6179	36932	1	0.02	=	=	46	0.0
395	Reference*	06p12.2	06-052.015764	3150	24567	1.04	0.05	=	=	55	0.0
454	Reference*	07q32.1	07-127.041158	2252	17801	1.02	0.02	=	=	40	0.0
190	Reference*	09p13.3	09-034.483216	5572	33110	1.01	0.03	=	=	42	-0.1
293	Reference*	12q12	12-038.037306	3499	23595	1.02	0.04	=	=	46	0.0
160	Reference*	15q21.1	15-042.674827	5171	29681	0.99	0.02	=	=	40	0.0
262	Reference*	18q21.1	18-045.743292	3846	25138	0.98	0.02	=	=	44	0.0
**Median value all probe values:**				**3220**	**22630**	**0.99**	**0.03**			**46**	**0**

Figure (xxviii) : MLPA results showing RAD51C:c.(837+21_838-21)_(965+21_966-21)dup

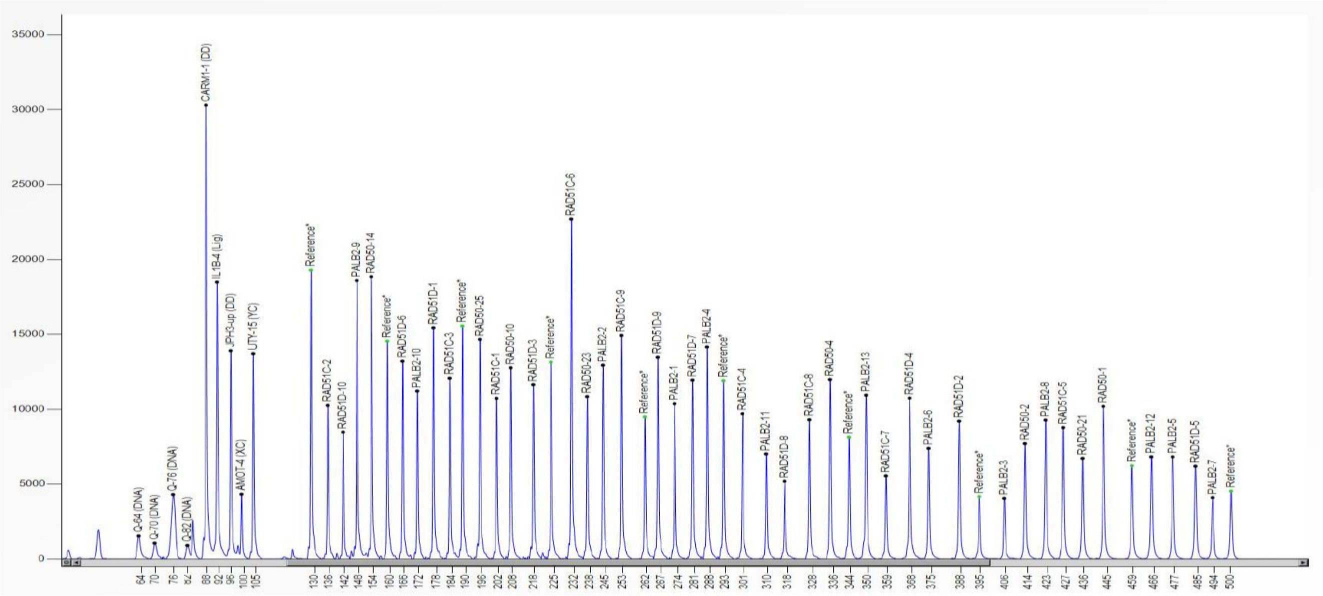

D [nt]	Gene-Exon	Chr.band	hg18 loc.	Height	Area	Ratio^H	Stdev	[REF]	[Sam]	Width	d[nt]
445	RAD50-1	05q31.1	05-131.920939	10187	104950	1.05	0.09	=	=	76	0.1
414	RAD50-2	05q31.1	05-131.922869	7704	73859	1.06	0.06	=	=	70	0.0
336	RAD50-4	05q31.1	05-131.942932	11975	104835	1.05	0.07	=	=	67	0.0
208	RAD50-10	05q31.1	05-131.954818	12766	101994	1.03	0.04	=	=	69	0.0
154	RAD50-14	05q31.1	05-131.967010	18844	145517	0.97	0.06	=	=	49	0.0
436	RAD50-21	05q31.1	05-131.981774	6706	69749	0.98	0.03	=	=	71	0.0
238	RAD50-23	05q31.1	05-132.001760	10834	89840	0.92	0.03	=	=	54	0.0
196	RAD50-25	05q31.1	05-132.005889	14659	120107	0.97	0.04	=	=	66	-0.1
350	PALB2-13	16p12.2	16-023.522409	10930	109022	1	0.06	=	=	70	0.0
466	PALB2-12	16p12.2	16-023.526633	6808	77317	1.01	0.04	=	=	72	0.1
310	PALB2-11	16p12.2	16-023.532832	7008	61786	0.89	0.08	=	=	70	0.0
172	PALB2-10	16p12.2	16-023.540210	11208	83196	0.93	0.08	=	=	43	0.0
148	PALB2-9	16p12.2	16-023.541776	18590	144103	0.97	0.04	=	=	43	0.0
423	PALB2-8	16p12.2	16-023.542867	9270	93288	1.06	0.05	=	=	64	0.0
494	PALB2-7	16p12.2	16-023.545151	4087	44800	0.98	0.06	=	=	61	-0.1
375	PALB2-6	16p12.2	16-023.548027	7383	71054	0.98	0.05	=	=	77	0.0
477	PALB2-5	16p12.2	16-023.549183	6808	72389	0.96	0.05	=	=	69	0.0
288	PALB2-4	16p12.2	16-023.554181	14152	121390	1	0.05	=	=	55	0.0
406	PALB2-3	16p12.2	16-023.556620	4041	39228	0.92	0.08	=	=	42	0.1
245	PALB2-2	16p12.2	16-023.556874	12937	104159	0.97	0.04	=	=	53	0.0
274	PALB2-1	16p12.2	16-023.559963	10364	85803	1.08	0.09	=	=	63	-0.1
142	RAD51D-10	17q12	17-030.452059	8463	60888	0.91	**0.12**	=	=	42	0.0
267	RAD51D-9	17q12	17-030.452394	13468	114564	1.05	0.07	=	=	57	-0.1
318	RAD51D-8	17q12	17-030.454373	5187	48853	0.98	0.06	=	=	72	-0.1
281	RAD51D-7	17q12	17-030.454635	11940	103255	1.07	0.04	=	=	67	0.0
166	RAD51D-6	17q12	17-030.457527	13203	99376	0.96	0.05	=	=	41	0.0
485	RAD51D-5	17q12	17-030.458217	6192	69491	1	0.07	=	=	69	0.0
368	RAD51D-4	17q12	17-030.458511	10734	108448	0.99	0.04	=	=	86	0.0
218	RAD51D-3	17q12	17-030.469551	11633	96074	1	0.06	=	=	60	0.1
388	RAD51D-2	17q12	17-030.470218	9198	88473	1.01	0.05	=	=	90	0.1
178	RAD51D-1	17q12	17-030.470829	15430	119962	1.01	0.05	=	=	59	0.0
202	RAD51C-1	17q22	17-054.125073	10718	82969	0.95	0.05	=	=	48	0.0
136	RAD51C-2	17q22	17-054.127407	10254	77382	0.88	0.1	=	=	52	-0.1
184	RAD51C-3	17q22	17-054.129144	12065	93341	0.94	0.06	=	=	53	0.0
301	RAD51C-4	17q22	17-054.135618	9695	83070	0.97	0.04	=	=	69	-0.1
427	RAD51C-5	17q22	17-054.142305	8766	90733	0.94	0.03	=	=	69	0.0
232	RAD51C-6	17q22	17-054.153266	22696	187574	***1.48***	0.06	>>*	>*	74	0.0
359	RAD51C-7	17q22	17-054.156418	5544	52880	**1.32**	**0.17**	?	?	73	0.0
328	RAD51C-8	17q22	17-054.164845	9294	81797	0.96	0.04	=	=	63	-0.1
253	RAD51C-9	17q22	17-054.166499	14922	121081	1.08	0.04	=	=	69	0.0
500	Reference*	02p13.2	02-071.750350	4526	49824	0.95	0.04	=	=	68	0.0
344	Reference*	02q12.3	02-108.971872	8129	75876	1.03	0.04	=	=	62	0.0
130	Reference*	04p13	04-042.278439	19288	147999	0.99	0.04	=	=	61	0.0
395	Reference*	06p12.2	06-052.015764	4161	42135	0.94	0.09	=	=	66	0.0
459	Reference*	07p14.3	07-030.637652	6232	67773	0.99	0.03	=	=	74	0.0
190	Reference*	09p13.3	09-034.483216	15551	120376	1.01	0.04	=	=	59	0.0
225	Reference*	09q34.13	09-134.207927	13145	104577	1.02	0.04	=	=	61	0.0
293	Reference*	12q12	12-038.037306	11900	103814	1.03	0.04	=	=	72	-0.1
160	Reference*	15q21.1	15-042.674827	14539	112165	0.97	0.04	=	=	53	0.0
262	Reference*	18q21.1	18-045.743292	9482	80337	1	0.09	=	=	60	0.0
**Median value all probe values:**				**10541**	**90286**	**0.99**	**0.05**			**65**	**0**

Figure (xxix): MLPA results showing RAD51D:c.(?_-21)_(*21_?)del

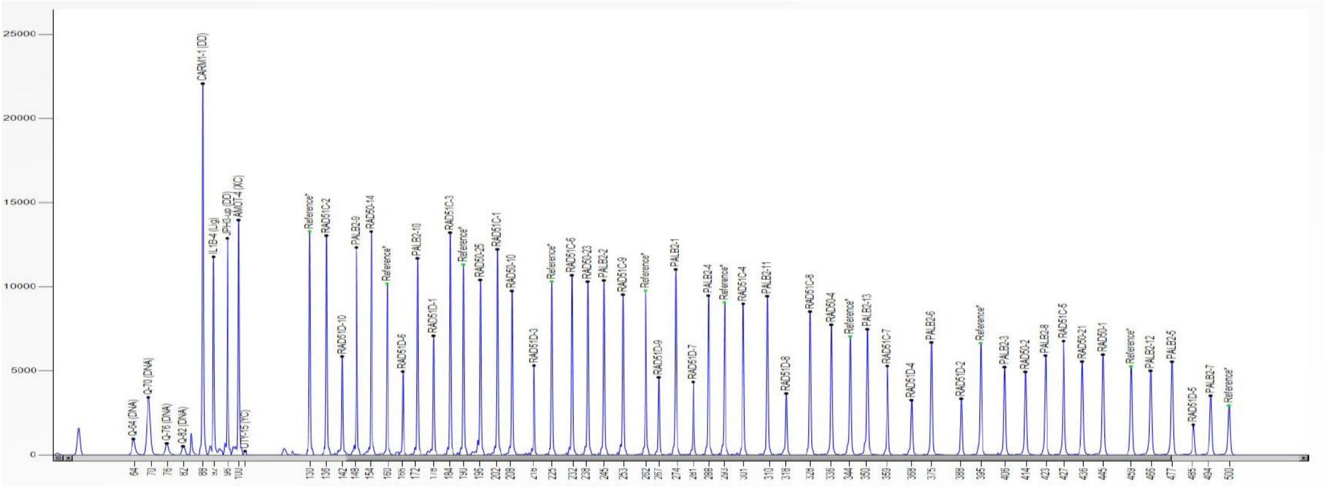

D [nt]	Gene-Exon	Chr.band	hg18 loc.	Height	Area	Ratio[H]	Stdev	[REF]	[Sam]	Width	d[nt]
445	RAD50-1	05q31.1	05-131.920939	5965	65328	1.1	0.09	=	=	46	0.0
414	RAD50-2	05q31.1	05-131.922869	4938	53056	1.1	0.08	=	=	55	0.1
336	RAD50-4	05q31.1	05-131.942932	7741	71575	1.02	0.08	=	=	46	0.0
208	RAD50-10	05q31.1	05-131.954818	9752	77104	1.02	0.06	=	=	67	0.0
154	RAD50-14	05q31.1	05-131.967010	13284	101352	1.06	**0.14**	=	=	72	0.0
436	RAD50-21	05q31.1	05-131.981774	5550	62696	1.06	**0.15**	=	=	60	0.0
238	RAD50-23	05q31.1	05-132.001760	10305	83464	0.98	0.05	=	=	63	0.1
196	RAD50-25	05q31.1	05-132.005889	10405	84873	0.96	0.05	=	=	48	0.0
350	PALB2-13	16p12.2	16-023.522409	7475	76893	0.98	0.08	=	=	63	0.0
466	PALB2-12	16p12.2	16-023.526633	5002	60915	1.04	0.08	=	=	65	0.1
310	PALB2-11	16p12.2	16-023.532832	9437	81123	1.02	0.05	=	=	58	0.0
172	PALB2-10	16p12.2	16-023.540210	11691	85158	0.98	0.06	=	=	54	0.0
148	PALB2-9	16p12.2	16-023.541776	12336	92804	1.05	0.08	=	=	48	0.0
423	PALB2-8	16p12.2	16-023.542867	5909	64207	1.05	0.09	=	=	58	0.1
494	PALB2-7	16p12.2	16-023.545151	3519	45093	1.05	0.09	=	=	76	0.1
375	PALB2-6	16p12.2	16-023.548027	6688	66704	1	0.05	=	=	42	-0.1
477	PALB2-5	16p12.2	16-023.549183	5541	66645	1.08	**0.11**	=	=	53	0.1
288	PALB2-4	16p12.2	16-023.554181	9473	82984	0.94	0.04	=	=	69	0.0
406	PALB2-3	16p12.2	16-023.556620	5223	53851	0.99	**0.11**	=	=	45	0.0
245	PALB2-2	16p12.2	16-023.556874	10378	82321	1.04	0.07	=	=	38	0.0
274	PALB2-1	16p12.2	16-023.559963	11031	91803	1.05	0.08	=	=	53	0.0
142	RAD51D-10	17q12	17-030.452059	5857	47840	**0.52**	0.04	<<*	=	61	0.0
267	RAD51D-9	17q12	17-030.452394	4612	37075	**0.51**	0.04	<<*	=	42	0.0
318	RAD51D-8	17q12	17-030.454373	3652	34304	**0.52**	0.04	<<*	=	42	-0.1
281	RAD51D-7	17q12	17-030.454635	4336	36623	**0.5**	0.04	<<*	=	36	0.0
166	RAD51D-6	17q12	17-030.457527	4959	36518	**0.51**	0.02	<<*	=	41	0.0
485	RAD51D-5	17q12	17-030.458217	1792	21726	**0.52**	0.06	<<*	=	43	0.1
368	RAD51D-4	17q12	17-030.458511	3254	35346	**0.49**	0.03	<<*	=	45	0.0
218	RAD51D-3	17q12	17-030.469551	5313	40291	**0.5**	0.03	<<*	=	34	0.0
388	RAD51D-2	17q12	17-030.470218	3341	32355	**0.54**	0.05	<<*	=	43	-0.1
178	RAD51D-1	17q12	17-030.470829	7083	53083	**0.58**	0.04	<<*	=	34	0.0
202	RAD51C-1	17q22	17-054.125073	12227	93386	1	0.06	=	=	54	0.0
136	RAD51C-2	17q22	17-054.127407	13041	97334	1.07	**0.13**	=	=	51	-0.1
184	RAD51C-3	17q22	17-054.129144	13218	97857	1.07	**0.15**	=	=	53	0.0
301	RAD51C-4	17q22	17-054.135618	8988	76342	1	0.06	=	=	40	0.0
427	RAD51C-5	17q22	17-054.142305	6769	75733	1.06	0.09	=	=	67	0.0
232	RAD51C-6	17q22	17-054.153266	10684	82830	1.05	0.07	=	=	46	0.1
359	RAD51C-7	17q22	17-054.156418	5288	51430	1	0.08	=	=	48	0.0
328	RAD51C-8	17q22	17-054.164845	8524	77092	0.97	0.04	=	=	46	-0.1
253	RAD51C-9	17q22	17-054.166499	9532	76900	0.97	0.04	=	=	47	0.0
500	Reference*	02p13.2	02-071.750350	2939	37695	1.06	**0.11**	=	=	51	0.1
344	Reference*	02q12.3	02-108.971872	7048	70369	1.03	0.06	=	=	65	0.0
130	Reference*	04p13	04-042.278439	13301	96211	1.07	**0.14**	=	=	41	-0.1
395	Reference*	06p12.2	06-052.015764	6652	69711	0.98	0.06	=	=	59	0.0
459	Reference*	07p14.3	07-030.637652	5275	61266	0.99	0.06	=	=	45	0.0
190	Reference*	09p13.3	09-034.483216	11317	84282	0.98	0.04	=	=	36	0.0
225	Reference*	09q34.13	09-134.207927	10315	78831	1.04	0.07	=	=	42	0.0
293	Reference*	12q12	12-038.037306	9072	77316	1.02	0.04	=	=	37	0.0
160	Reference*	15q21.1	15-042.674827	10185	73851	1.04	**0.11**	=	=	45	0.0
262	Reference*	18q21.1	18-045.743292	9769	80889	0.99	0.04	=	=	44	0.0
**Median value all probe values:**				**7279**	**72713**	**1**	**0.06**			**47.5**	**0.01**

Figure (xxx) : LR-PCR (Long Range PCR) and sequencing results confirming POLE:c.5959_5960ins198,p.(Leu1986_Pro1987ins66)

a) Breakpoint detection of the POLE gene insertion by pileup analysis

3' breakpoint Pile up

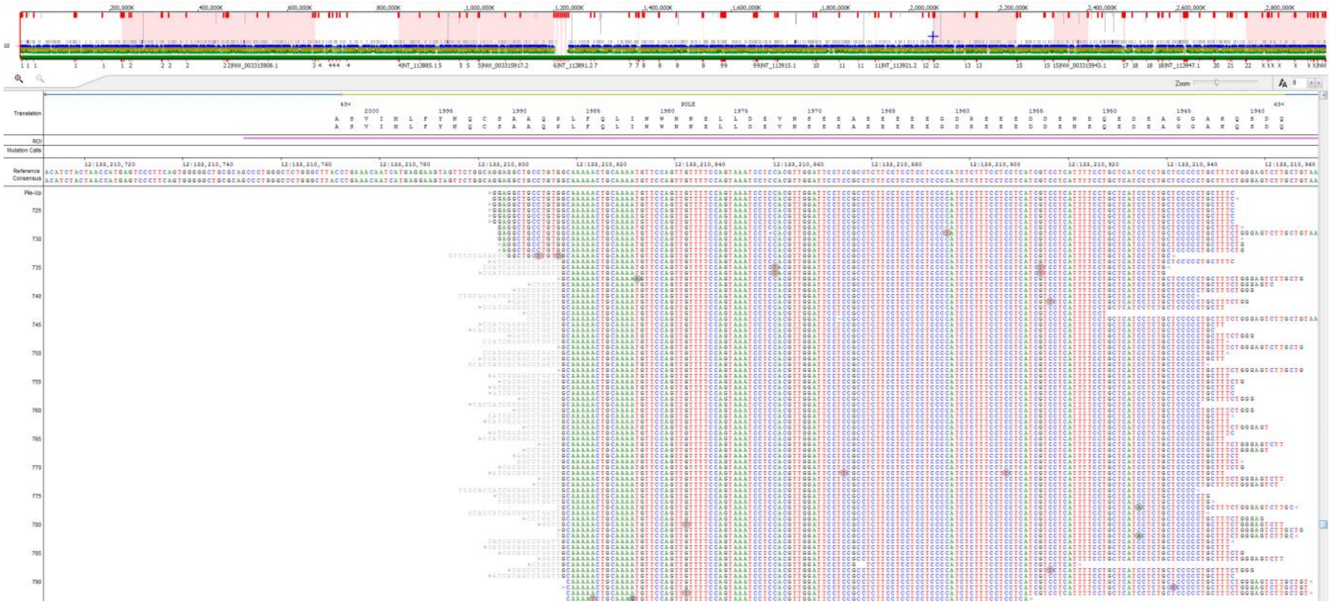

5' breakpoint Pile up

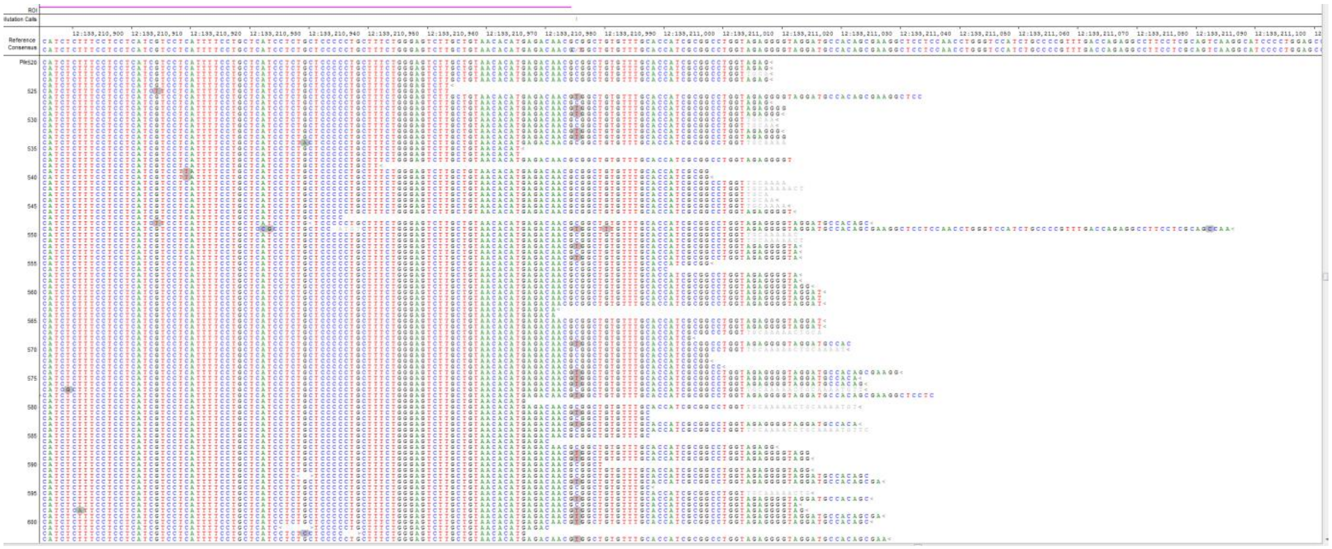

b) Agarose gel image showing Long range PCR (LRPCR) (A0: Ladder ; A1: Patient sample; B1 : Blank)

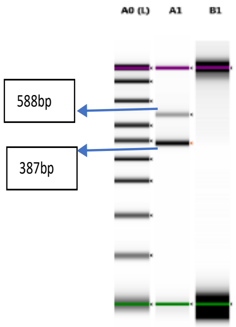

c) Normalized CNV plot showing POLE gene insertion

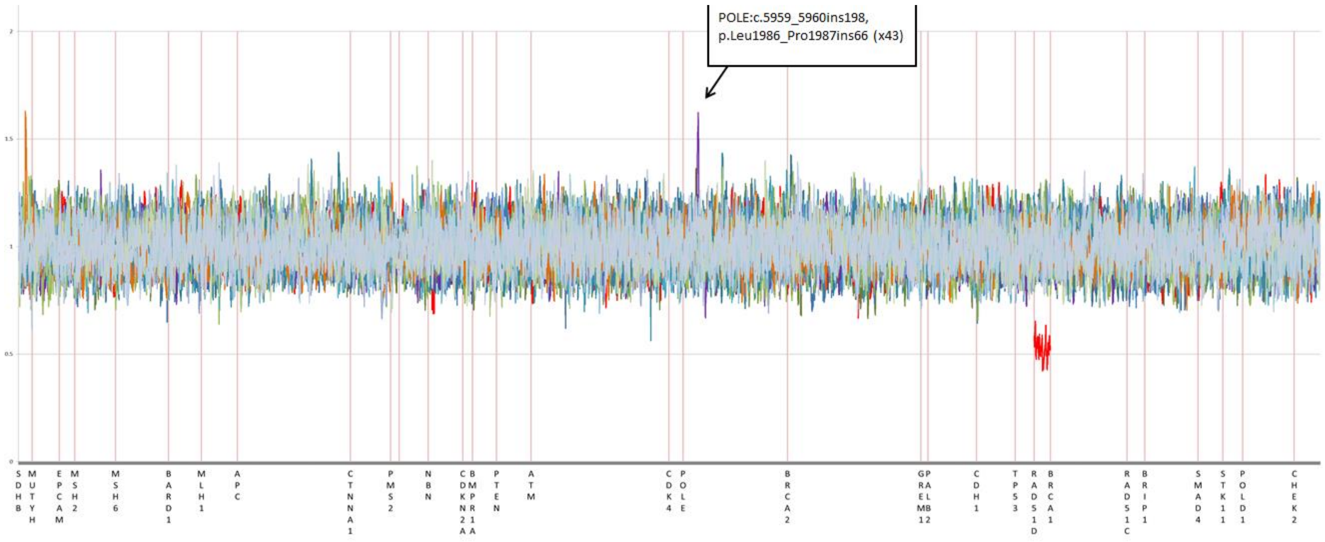

d) Sanger sequencing electropherogram analysis in the forward direction

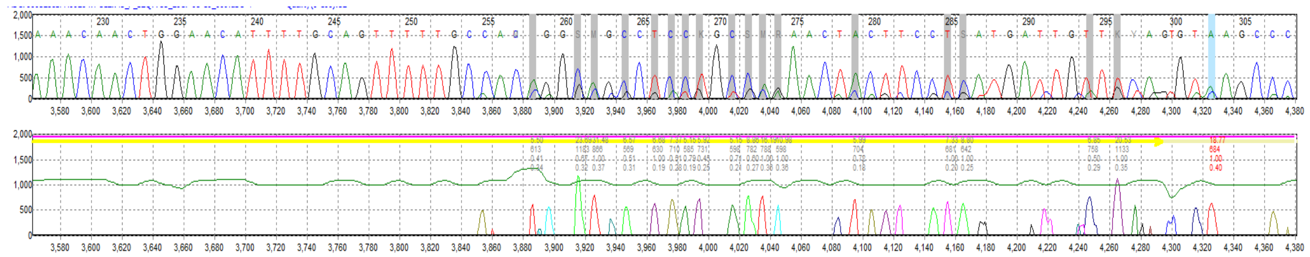

e) Sequence analysis showing inserted sequence

c.5959_5960ins198, p.Leu1986_Pro1987ins66

(Inserted region includes c.5812-49_5959dup region with an additional 5' A)

POLEx43 Sequence

GGCAGATGGACCCAGGTTGGAGGAGCCTTCGCTGTGGCA**TCCTACCCCTCT**ACCAGGCCGCGATGGTGCAAACACAGCC
GCGTTGTCTCATGTGTTACAG**CAAGACTCCCAGAAAGCAGGGGGAGCAGAGGATGAGCAGGAAAATGAGGACGATGA**
GGAGGAAAGAGATGGGGAGGAGGAGGAAGAGGCGGAGGAATCCAACGTGGAGGATTTACTGGAAAACAACTGGAA
CATTTTGCAGTTTTTGCCACAGGCAGCCTCCTGCCAGAACTACTTCCTCATGATTGTTTCAGGTAAGCCCAGAGCCCAGG
GCTGCGCAGCCCCCACTGAAGGGACTCATGGTTAGTAGATGTGGCTCCTGTGCCATGGTATCGCAAGAAGGGAGACAGG
GAA

NGS Pileup Fragment (5')

GAACATTTTGCAGTTTTTGCAACCAGGCCGCGATGGTGCAAACACAGCCG

NGS Pileup Fragment (3')

AAAACAACTGGAACATTTTGCAGTTTTTGCAACCAGGCCGCGATGGTGCAA

POLEx43 Sequence with insertion

GGCAGATGGACCCAGGTTGGAGGAGCCTTCGCTGTGGCATCCTACCCCTCTACCAGGCCGCGATGGTGCAAACACAGCC
GCGTTGTCTCATGTGTTACAG**CAAGACTCCCAGAAAGCAGGGGGAGCAGAGGATGAGCAGGAAAATGAGGACGATG**
AGGAGGAAAGAGATGGGGAGGAGGAGGAAGAGGCGGAGGAATCCAACGTGGAGGATTTACTGGAAAACAACTGG
AACATTTTGCAGTTTTTGCAACCAGGCCGCGATGGTGCAAACACAGCCGCGTTGTCTCATGTGTTACAG**CAAGACTCCC**
AGAAAGCAGGGGGAGCAGAGGATGAGCAGGAAAATGAGGACGATGAGGAGGAAAGAGATGGGGAGGAGGAGGA
AGAGGCGGAGGAATCCAACGTGGAGGATTTACTGGAAAACAACTGGAACATTTTGCAGTTTTTGCCACAGGCAGCCT
CCTGCCAGAACTACTTCCTCATGATTGTTTCAGGTAAGCCCAGAGCCCAGGGCTGCGCAGCCCCCACTGAAGGGACTCA
TGGTTAGTAGATGTGGCTCCTGTGCCATGGTATCGCAAGAAGGGAGACAGGGAA

Figure (XXXi) PTEN:c.80-958_80-60del *

a) Breakpoint detection of the PTEN gene deletion by pileup analysis

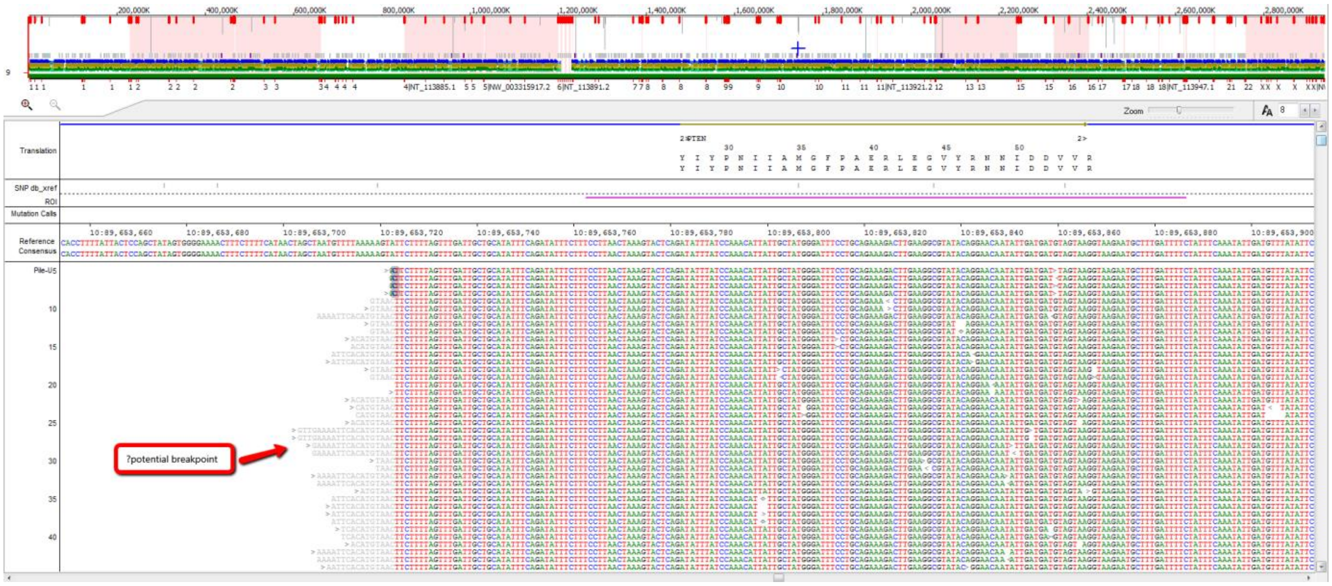

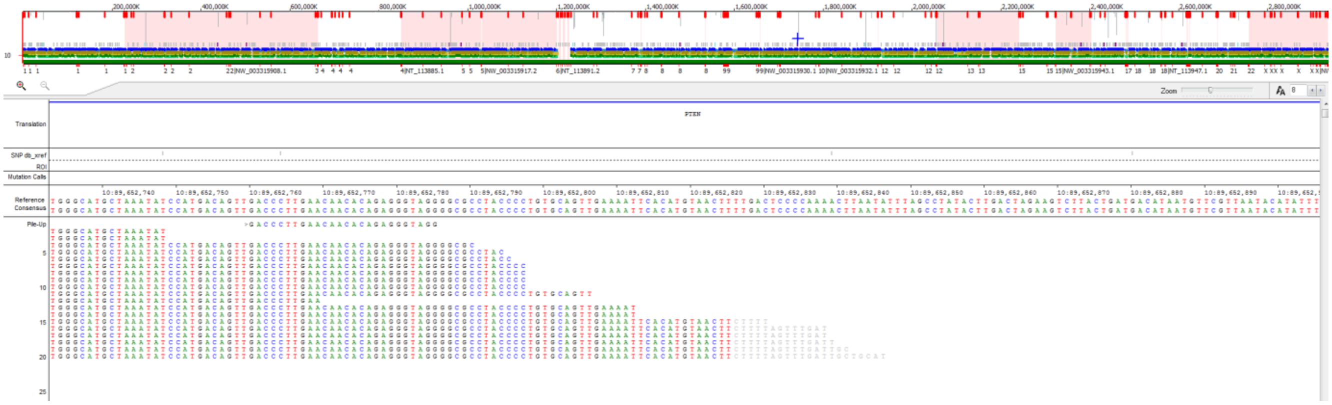

b) Agarose gel image showing Long range PCR (LRPCR) for PTEN gene (upstream promoter region: A0: Ladder ; B1: Normal controls; C1: Patient sample; B1 : Blank)

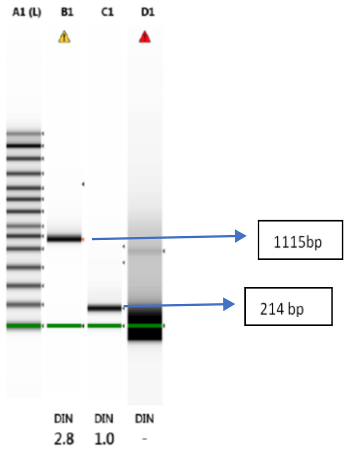

c) Normalized CNV plot showing PTEN gene deletion

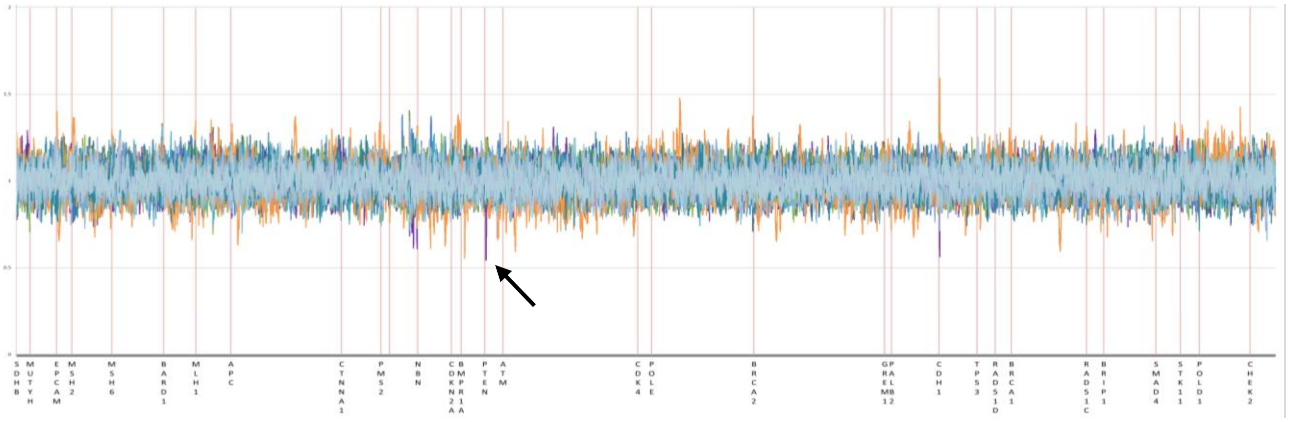

c) Sanger sequencing electropherogram of PTEN gene (upstream promoter region) showing deletion point

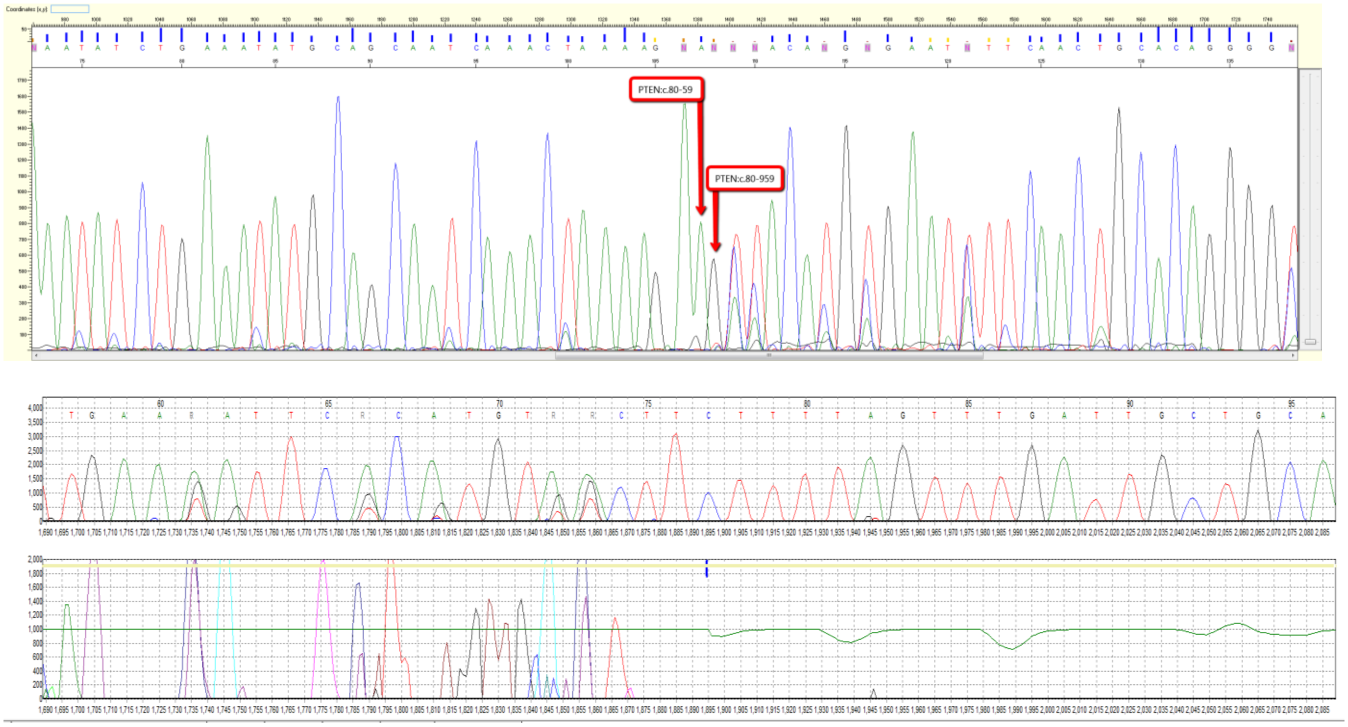

Supplement: Supplementary Table 1 — Guidelines for referral for hereditary cancer predisposition genetic testing. [file Data_Sheet_1.zip › Supplementary files/Supplementary Table S6.pdf]
