# Supplementary material for: Analysis of Sequence and Copy Number Variants in Canadian Patient Cohort With Familial Cancer Syndromes Using a Unique Next Generation Sequencing Based Approach
Source: Front Genet. 2021 Jul 13;12:698595. doi: 10.3389/fgene.2021.698595 (PMC8314385; doi:10.3389/fgene.2021.698595)
Supplement: Supplementary Table 1 — Guidelines for referral for hereditary cancer predisposition genetic testing. [file Data_Sheet_1.zip › Supplementary files/Supplementary Table S7.docx]

**Supplementary Table S7**: Number of patients with each cancer type positive for a pathogenic variant in genes across all sub-panels

| Cancer Type | APC | ATM | BARD1 | BRCA1 | BRCA2 | BRIP1 | CHEK2 | MLH1 | MSH2 | MSH6 | PALB2 | PMS2 | RAD51C | RAD51D | TP53 |
| --- | --- | --- | --- | --- | --- | --- | --- | --- | --- | --- | --- | --- | --- | --- | --- |
| Breast | 5 | 21 | 3 | 33 | 27 | 6 | 36 | 0 | 3 | 1 | 13 | 3 | 2 | 4 | 7 |
| Ovarian | 0 | 5 | 0 | 25 | 18 | 2 | 3 | 0 | 4 | 1 | 2 | 1 | 7 | 5 | 4 |
| Colorectum | 2 | 1 | 1 | 0 | 0 | 1 | 0 | 6 | 3 | 5 | 0 | 2 | 0 | 0 | 1 |
| Polyps | 4 | 2 | 0 | 0 | 1 | 0 | 1 | 2 | 0 | 1 | 0 | 2 | 0 | 0 | 1 |
| Gastric | 0 | 1 | 0 | 0 | 3 | 0 | 0 | 0 | 1 | 0 | 0 | 1 | 0 | 0 | 2 |
| Uterine | 0 | 1 | 0 | 0 | 3 | 0 | 1 | 2 | 1 | 1 | 0 | 1 | 1 | 0 | 0 |
| Pancreas | 0 | 0 | 0 | 0 | 0 | 0 | 0 | 0 | 0 | 0 | 2 | 0 | 0 | 0 | 0 |
| Prostate | 0 | 0 | 0 | 0 | 0 | 0 | 2 | 0 | 0 | 0 | 0 | 0 | 0 | 0 | 0 |
| Others | 1 | 1 | 0 | 0 | 2 | 0 | 2 | 1 | 1 | 0 | 1 | 0 | 1 | 0 | 0 |
| Not specified | 3 | 3 | 0 | 5 | 6 | 0 | 5 | 1 | 0 | 1 | 3 | 2 | 0 | 2 | 0 |
| No cancer | 1 | 1 | 0 | 4 | 4 | 0 | 4 | 0 | 0 | 0 | 1 | 1 | 0 | 0 | 3 |
| Total | 16 | 36 | 4 | 67 | 64 | 9 | 54 | 12 | 13 | 10 | 22 | 13 | 11 | 11 | 18 |
